# The risks of adverse events with venlafaxine for adults with major depressive disorder: a systematic review of randomised clinical trials with meta-analysis and Trial Sequential Analysis

**DOI:** 10.1017/S2045796024000520

**Published:** 2024-10-23

**Authors:** C. B. Kamp, J. J. Petersen, P. Faltermeier, S. Juul, F. Siddiqui, J. Moncrieff, M. A. Horowitz, M. P. Hengartner, I. Kirsch, C. Gluud, J. C. Jakobsen

**Affiliations:** 1 Copenhagen Trial Unit, Centre for Clinical Intervention Research, The Capital Region, Copenhagen University Hospital – Rigshospitalet, Copenhagen Ø, Denmark; 2 Department of Regional Health Research, Faculty of Health Sciences, University of Southern Denmark, Odense, Denmark; 3 MSH Medical School Hamburg, University of Applied Sciences and Medical University, Hamburg, Germany; 4 Stolpegaard Psychotherapy Centre, Mental Health Services in the Capital Region of Denmark, Gentofte, Denmark; 5 Department of Psychology, University of Copenhagen, Copenhagen, Denmark; 6 Division of Psychiatry, University College London, London, UK*; 7 Research and Development Department, North East London NHS Foundation Trust (NELFT), London, UK; 8 Department of Applied Psychology, Zurich University of Applied Sciences Zurich, Switzerland; 9 Program in Placebo Studies, Harvard Medical School, Boston, MA, USA

**Keywords:** adverse effects, antidepressants, depression, systematic reviews

## Abstract

**Aims:**

Venlafaxine is used to treat depression worldwide. Previous reviews have demonstrated that venlafaxine lowers scores on depression rating scales, producing statistically significant results but the relevance to patients remains uncertain. Knowledge of the incidence of the adverse effects associated with venlafaxine has previously been based on the results of non-randomised studies. Our primary objective was to assess the risks of adverse events with venlafaxine in the treatment of adults with major depressive disorder in randomised trials.

**Methods:**

We searched relevant databases and other sources from inception to 7 March 2024 for randomised clinical trials comparing venlafaxine versus placebo or no intervention in adults with major depressive disorder. Data were synthesised using meta-analysis and Trial Sequential Analysis. The primary outcomes were suicides or suicide attempts, serious adverse events and non-serious adverse events.

**Results:**

We included 28 trials randomising 6,253 participants to venlafaxine versus placebo. All results were at high risk of bias, and the certainty of the evidence was very low. All trials assessed outcomes at a maximum of 12 weeks after randomisation. Meta-analysis and Trial Sequential Analysis showed insufficient information to assess the effects of venlafaxine on the risks of suicides or suicide attempts. Meta-analysis showed evidence of harm of venlafaxine versus placebo on serious adverse events (risk ratio: 2.66; 95% confidence interval: 1.67–4.25; *p* < 0.01; 22 trials), mainly due to a higher risk of sexual dysfunction and anorexia. Meta-analysis showed that venlafaxine also increased the risk of several non-serious adverse events: nausea, dry mouth, dizziness, sweating, somnolence, constipation, nervousness, insomnia, asthenia, tremor and decreased appetite.

**Conclusions:**

Short-term results show that venlafaxine has uncertain effects on the risks of suicides but increases the risks of serious adverse events (especially sexual dysfunction and anorexia) and many non-serious adverse events. The long-term effects of venlafaxine for major depressive disorder are unknown. It is a particular cause for concern that there are no data on the long-term adverse effects of venlafaxine given that so many people use these drugs for several years.

## Introduction

Major depressive disorder is a psychiatric condition characterised by depressed mood, feelings of hopelessness and lack of interest or motivation (American Psychiatric Association, 2013). Major depressive disorder is associated with an increased risk of suicidal behaviour, reduced quality of life and impaired cognition (Chen and Dilsaver, 1996; Ishak *et al.*, 2015; Kessler *et al.*, 1999; Pan *et al.*, 2019; Qin, 2011; Saragoussi *et al.*, 2018). Major depressive disorder affects approximately 280 million people globally, causing a severe burden on patients and societies (World Health Organization, 2023). The serotonin-norepinephrine reuptake inhibitor, venlafaxine, is approved for the treatment of major depressive disorder in several countries, including the United States, EU countries and the United Kingdom (European Medicines Agency, 2008; National Health Service, 2018; Singh and Saadabadi, 2021). Venlafaxine is often recommended as a second-line treatment for major depressive disorder (Malone, 2007; National Health Service, 2018; NHS East and North Hertfordshire Clinical Commissioning Group and NHS Herts Valleys Clinical Commissioning Group, 2018; Singh and Saadabadi, 2021).

Previous reviews have demonstrated that venlafaxine lowers scores on depression rating scales, producing statistically significant results but the relevance to patients remains uncertain. (Cipriani *et al.*, 2018; Hengartner and Ploderl, 2022; Jakobsen *et al.*, 2020; Schueler *et al.*, 2011). Previously, understanding of the adverse effects of venlafaxine has primarily been based on results of non-randomised studies, which may under- or overestimate the occurrence of adverse effects due to confounding and biased reporting (Vandenbroucke, 2006). Previous reviews have not systematically assessed the risks of all adverse events using data from randomised clinical trials (Cipriani *et al.*, 2018; Schueler *et al.*, 2011).

Our primary objective was to assess the risks of adverse events with venlafaxine versus placebo or ‘active placebo’ in the treatment of adults with major depressive disorder.

## Methods

We report this systematic review based on the Preferred Reporting Items for Systematic Reviews and Meta-Analysis (PRISMA) guidelines (**Supplementary File 1**) (Liberati *et al.*, 2009; Moher *et al.*, 2015). The methodology was predefined in our protocol based on the recommendations of the Cochrane Handbook of Systematic Reviews of Interventions (Higgins *et al.*, 2022; Jorgensen *et al.*, 2023). Our protocol was also preregistered in the PROSPERO database (ID: CRD42022315395). This systematic review is a part of a larger project investigating the beneficial and harmful effects of all antidepressants for major depressive disorder (Jorgensen *et al.*, 2021; Juul *et al.*, 2021b; Kamp *et al.*, 2024; Siddiqui *et al.*, 2021).

### Search strategy

An information specialist searched the Cochrane Central Register of Controlled Trials (CENTRAL), Medical Literature Analysis and Retrieval System Online (MEDLINE), Excerpta Medica Database (Embase), Latin American and Caribbean Health Sciences Literature (LILACS), PsycINFO, Science Citation Index Expanded (SCI-EXPANDED), Social Sciences Citation Index (SSCI) and Conference Proceedings Citation Index–Social Science & Humanities (CPCI-SSH) to identify relevant trials. We searched all databases from their inception to 7 March 2024. For a detailed search strategy for all electronic databases, see **Supplementary File 2**. We searched clinical trial registers and websites of pharmaceutical companies to identify unpublished data. We requested clinical study reports from the U.S. Food and Drug Administration, European Medicines Agency and national medicines agencies (Sharma *et al.*, 2016).

### Selection criteria

Our eligibility criteria included randomised clinical trials of adults with a primary diagnosis of major depressive disorder as defined by standardised diagnostic criteria, such as the Diagnostic and Statistical Manual of Mental Disorders (American Psychiatric Association, 2013) or the International Classification of Diseases (World Health Organization, 2021). Major depressive disorder had to be the primary diagnosis, and we did, therefore, not include trials randomising participants with a non-psychiatric primary diagnosis and comorbid major depressive disorder. If a trial reported data where only a subset of participants was eligible (e.g. a combination of adolescents and adults), we only included those that fulfilled our inclusion criteria, so it required that data could be obtained for that specific group. The experimental intervention was venlafaxine, and the control interventions were placebo, ‘active placebo’ or no intervention.

### Data extraction and risk of bias assessment

Three review authors (CBK, SJ and FS) screened abstracts and articles using Covidence to identify relevant trials (Covidence, 2024). Three review authors (JJP, PF and CBK) extracted data and assessed risk of bias based on the Cochrane Risk of Bias tool, version 2 (RoB 2) (Higgins *et al.*, 2022; Sterne *et al.*, 2019). Screening, data extraction and risk of bias assessments were performed independently by at least two review authors. Any discrepancies were resolved through internal discussion or, if required, through discussion with the last author (JCJ). We emailed all corresponding authors with available contact information to confirm data and risk of bias assessments.

### Outcomes and subgroup analyses

The primary outcomes were suicides or suicide attempts, serious adverse events (International Council on Harmonisation of Technical Requirements for Registration of Pharmaceuticals for Human Use, 2015) and non-serious adverse events (Jorgensen *et al.*, 2023). Exploratory outcomes were depressive symptoms measured on the 17-item Hamilton Depression Rating Scale (HDRS-17), quality of life, all adverse events, suicidal ideation, level of functioning, depressive symptoms measured on the Montgomery–Asberg Depression Rating Scale (MADRS) (Montgomery and Åsberg, 1979), the Beck’s Depression Inventory (BDI) (Beck *et al.*, 1996) or HDRS-6 (López-Pina *et al.*, 2009; Timmerby *et al.*, 2017), withdrawal symptoms and proportion of participants that guessed their treatment allocation (Jorgensen *et al.*, 2023). Short-term follow-up was defined as the assessment closest to 3 months after randomisation, and long-term follow-up was defined as 6 months or more after randomisation. We also planned several subgroup analyses (Jorgensen *et al.*, 2023).

### Assessment of statistical and clinical significance

We performed meta-analyses according to the recommendations of the Cochrane Handbook for Systematic Reviews of Interventions (Higgins *et al.*, 2022), Keus *et al.* (2010) and the eight-step procedure by Jakobsen *et al.* (2014). We adjusted the threshold for statistical significance by the number of primary outcomes and therefore used a *p*-value of 0.025 or less as threshold (Jakobsen *et al.*, 2014). We analysed data using the software Stata version 17 (Statacorp, 2019). We used both random-effects (Hartung–Knapp–Sidik–Jonkman) (Inthout *et al.*, 2014) and fixed-effect model meta-analyses (Mantel–Haenszel for dichotomous outcomes and inverse variance for continuous outcomes) to assess intervention effects (DeMets, 1987; Higgins *et al.*, 2022). We primarily reported the most conservative result (highest *p*-value), and considered the less conservative result a sensitivity analysis (Jakobsen *et al.*, 2014). Trial Sequential Analysis was used to control for random errors by estimating the diversity-adjusted required information size (Brok *et al.*, 2008a, 2008b; Copenhagen Trial Unit – Centre for Clinical Intervention Research, 2023; Imberger *et al.*, 2016; Thorlund *et al.*, 2010, 2008, 2017; Wetterslev *et al.*, 2008, 2009). We estimated the required information size based on the observed proportion of participants with an outcome in the control group, a relative risk reduction or a relative risk increase of 20%, an alpha of 2.5%, a beta of 10% and the observed diversity. The 20% relative risk reduction or increase was chosen as it is a common effect size when an intervention is beneficial and may be important to patients. We calculated Bayes factor for all primary outcomes. We used Grading Recommendations Assessment Development Evaluation (GRADE) to assess the certainty of evidence for the primary outcomes (Guyatt *et al.*, 2011, 2008; Schünemann *et al.*, 2003). Please see the published protocol for a detailed description of the statistical analysis plan (Jorgensen *et al.*, 2023).

## Results

**Figure 1.**
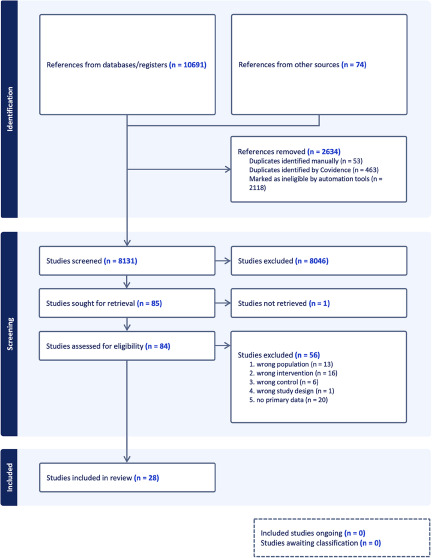
PRISMA flow diagram.

A total of 28 trials randomising 6,253 participants were included (Alvarez *et al.*, 2012; Cunningham, 1997; Cunningham *et al.*, 1994; Guelfi *et al.*, 1995; Hewett *et al.*, 2009, 2010; Higuchi *et al.*, 2016; Hopkins *et al.*, 2013; Hunter *et al.*, 2010; Khan *et al.*, 1991, 1998; Learned *et al.*, 2012; Lieberman *et al.*, 2008; Luthringer *et al.*, 1996; Mendels *et al.*, 1993; Nemeroff *et al.*, 2007; Rudolph *et al.*, 1998; Rudolph and Feiger, 1999; Schatzberg and Roose, 2006; Schweizer *et al.*, 1994, 1991; Sharma *et al.*, 2016; Sheehan *et al.*, 2009; Silverstone and Ravindran, 1999; Thase, 1997; Wyeth-Ayerst, 1991a, 1991b, 1992, 1991c; Wyeth-Ayerst Research, 1996) (Fig. 1). We identified unpublished data through clinical study reports for 11 trials (Alvarez *et al.*, 2012; Eudract 2004-000562-13, 2016; Eudract Number 2007-007025-51, 2010; Hewett *et al.*, 2009; Learned *et al.*, 2012; Sharma *et al.*, 2016; Wyeth-Ayerst, 1991a, 1991b, 1992, 1991c; Wyeth-Ayerst Research, 1996). Most trials included both men and women aged 18 years or older with a primary diagnosis of major depressive disorder (**Supplementary Table 1**). One trial only included participants aged 65 years or older (Schatzberg and Roose, 2006). The mean HDRS entry scores ranged from 22.4 to 30.6 (**Supplementary Table 1**). All trials involved an inert placebo control, i.e. we did not identify any trials using an ‘active placebo’ or no intervention as control interventions. All trials assessed outcomes at a maximum of 12 weeks after randomisation. Most trials did not adequately report the proportion of participants with missing data at follow-up, and it was, therefore, not possible to perform ‘best-worst/worst-best’ sensitivity analyses. All trials were assessed at overall high risk of bias, particularly due to lack of information, missing data, lack of information on blinding, risk of unblinding due to adverse events or other noticeable changes, inappropriate analysis methods and poor reporting (**Supplementary Fig. 1**). Three trials did not report any relevant data (Hopkins *et al.*, 2013; Hunter *et al.*, 2010).

### Primary outcomes

#### Suicides or suicide attempts

**Figure 2.**
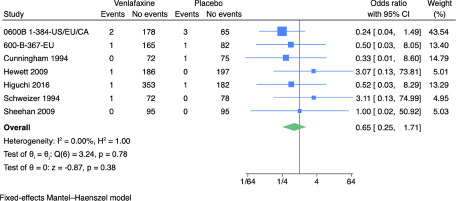
Meta-analysis of venlafaxine versus placebo on suicides or suicide attempts.

Only 7 of 28 trials reported results on suicides or suicide attempts (Hewett *et al.*, 2009; Higuchi *et al.*, 2016; Sharma *et al.*, 2016; Sheehan *et al.*, 2009; Wyeth-Ayerst, 1991b, 1992; Wyeth-Ayerst Research, 1996). All trials only assessed outcomes at the end of the treatment period, i.e. 6–8 weeks after randomisation. A total of 6/1,127 (0.5%) experimental participants attempted or committed suicide compared with 6/780 (0.8%) control participants. Meta-analysis showed no evidence of a difference between venlafaxine versus placebo on suicides or suicide attempts (odds ratio: 0.65; 95% confidence interval (CI): 0.25–1.71; *p* = 0.38; 7 trials; Bayes factor: 0.74) (Fig. 2). Visual inspection of the forest plot and statistical tests (*I*^2^ = 0.0%) indicated no clear signs of heterogeneity. Trial Sequential Analysis showed that we did not have enough information to confirm or reject the hypothesis that venlafaxine influenced the risk of suicides or suicide attempts with a relative risk reduction of 20% (no graph produced). This outcome result was assessed as overall high risk of bias, and the certainty of the evidence was very low (**Supplementary Tables 2–3**).

Tests of interaction comparing the effects of using a placebo washout period prior to randomisation showed no evidence of a difference (*p* = 1.00) (**Supplementary Fig. 2**). The remaining predefined subgroup analyses could not be performed due to a lack of relevant data.

#### Serious adverse events

**Figure 3.**
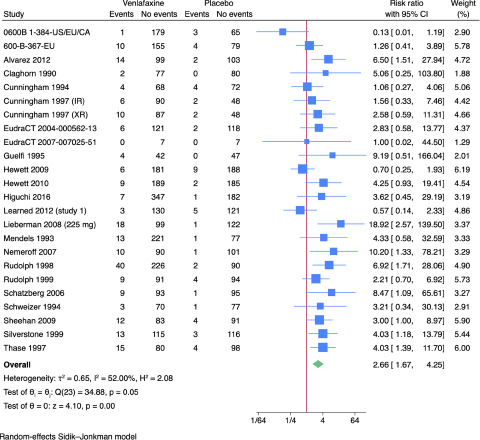
Meta-analysis of venlafaxine versus placebo on serious adverse events.

Twenty-two trials reported results on serious adverse events (Alvarez *et al.*, 2012; Cunningham, 1997; Eudract 2004-000562-13, 2016; Eudract Number 2007-007025-51, 2010; Hewett *et al.*, 2009, 2010; Higuchi *et al.*, 2016; Learned *et al.*, 2012; Lieberman *et al.*, 2008; Mendels *et al.*, 1993; Nemeroff *et al.*, 2007; Rudolph *et al.*, 1998; Rudolph and Feiger, 1999; Schatzberg and Roose, 2006; Sharma *et al.*, 2016; Sheehan *et al.*, 2009; Silverstone and Ravindran, 1999; Thase, 1997; Wyeth-Ayerst, 1991a, 1991b, 1992, 1991c; Wyeth-Ayerst Research, 1996). All trials only assessed outcomes at the end of the treatment period, i.e. 4–12 weeks after randomisation. A total of 224/3,164 (7.1%) experimental participants had one or more serious adverse event compared with 58/2,362 (2.5%) control participants. Meta-analysis showed evidence of a harmful effect of venlafaxine versus placebo on serious adverse events (risk ratio (RR): 2.66; 95% CI: 1.67–4.25; *p* < 0.01; 22 trials; Bayes factor: 0.06) (Fig. 3). Visual inspection of the forest plot and statistical tests (*I*^2^ = 52.0%) indicated heterogeneity that could not be resolved. Trial Sequential Analysis showed that we did not have enough information to confirm or reject the hypothesis that venlafaxine influenced the risk of serious adverse events with a relative risk reduction or increase of 20% (**Supplementary Fig. 3**). This outcome result was assessed as overall high risk of bias, and the certainty of the evidence was very low (**Supplementary Tables 2–3**).

Tests of interaction comparing the effects of using a placebo washout period prior to randomisation showed no evidence of a difference (*p* = 0.87) (**Supplementary Fig. 4**). The remaining predefined subgroup analyses could not be performed due to a lack of relevant data.

When each specific serious adverse event was analysed separately, 2/11 meta-analyses showed evidence of a harmful effect of venlafaxine versus placebo: sexual dysfunction (RR: 6.49; 95% CI: 3.02–13.93; *p* < 0.01; *I*^2^ = 1.9%; 8 trials; number needed to harm (NNH): 12) (**Supplementary Fig. 5**) and anorexia (RR: 3.23; 95% CI: 1.75–5.97; *p* < 0.01; *I*^2^ = 44.7%; 9 trials; NNH: 14) (**Supplementary Fig. 6**). The remaining meta-analyses showed no evidence of differences (**Supplementary Tables 4–5, Supplementary Fig. 7–15**).

#### Non-serious adverse events

**Figure 4.**
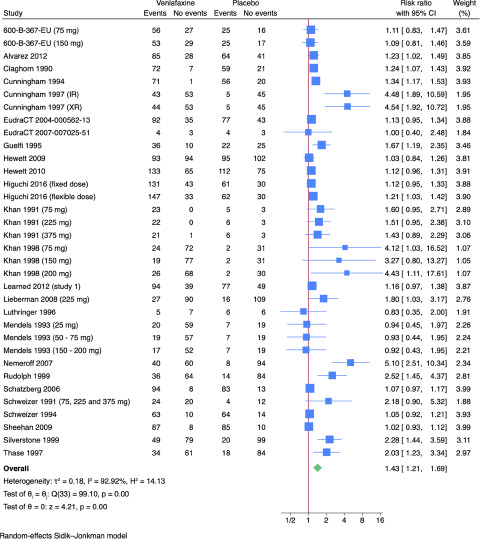
Meta-analysis of venlafaxine versus placebo on non-serious adverse events.

Twenty-four trials reported results on non-serious adverse events (Alvarez *et al.*, 2012; Cunningham, 1997; Eudract 2004-000562-13, 2016; Eudract Number 2007-007025-51, 2010; Guelfi *et al.*, 1995; Hewett *et al.*, 2009, 2010; Higuchi *et al.*, 2016; Khan *et al.*, 1991, 1998; Learned *et al.*, 2012; Lieberman *et al.*, 2008; Nemeroff *et al.*, 2007; Rudolph *et al.*, 1998; Rudolph and Feiger, 1999; Schatzberg and Roose, 2006; Schweizer *et al.*, 1994, 1991; Sheehan *et al.*, 2009; Silverstone and Ravindran, 1999; Thase, 1997; Wyeth-Ayerst, 1991b, 1992, 1991c; Wyeth-Ayerst Research, 1996). All trials only assessed outcomes at the end of the treatment period, i.e. 4–12 weeks after randomisation. A total of 1,804/3,127 (57.7%) experimental participants had one or more non-serious adverse events compared with 1,111/2,356 (47.2%) control participants. Meta-analysis showed evidence of a harmful effect of venlafaxine versus placebo on non-serious adverse events (RR: 1.43; 95% CI: 1.21–1.69; *p* < 0.01; 24 trials; Bayes factor: 0.001) (Fig. 4). Visual inspection of the forest plot and statistical tests (*I*^2^ = 92.9%) indicated heterogeneity that could not be resolved. Trial Sequential Analysis showed that we did not have enough information to confirm or reject the hypothesis that venlafaxine influenced the risk of non-serious adverse events with a relative risk reduction or increase of 20% (**Supplementary Fig. 16**). This outcome result was assessed as overall high risk of bias, and the certainty of the evidence was very low (**Supplementary Tables 2–3**).

Tests of interaction comparing the effects of using a placebo washout period prior to randomisation showed evidence of a difference (*p* < 0.01) (**Supplementary Fig. 17**). When the subgroup of trials with a placebo washout period was analysed separately, meta-analysis showed evidence of a harmful effect of venlafaxine (RR: 1.63; 95% CI: 1.30–2.05; *p* < 0.01; 15 trials). When the subgroup of trials without a placebo washout period was analysed separately, meta-analysis showed evidence of a harmful effect (RR: 1.13; 95% CI: 1.04–1.23; *p* < 0.01; 9 trials). The remaining predefined subgroup analyses could not be performed due to a lack of relevant data.

When each specific non-serious adverse event was analysed separately, 11/47 meta-analyses showed evidence of a harmful effect of venlafaxine versus placebo: nausea (RR: 2.72; 95% CI: 2.26–3.28; *p* < 0.01; *I*^2^ = 46.4%; 23 trials; NNH: 5) (**Supplementary Fig. 18**), dry mouth (RR: 2.16; 95% CI: 1.71–2.74; *p* < 0.01; *I*^2^ = 40.7%; 21 trials; NNH: 10) (**Supplementary Fig. 19**), dizziness (RR: 2.49; 95% CI: 1.90–3.26; *p* < 0.01; *I*^2^ = 37.9%; 20 trials; NNH: 11) (**Supplementary Fig. 20**), sweating (RR: 3.99; 95% CI: 2.88–5.54; *p* < 0.01; *I*^2^ = 20.5%; 20 trials; NNH: 11) (**Supplementary Fig. 21**), somnolence (RR: 2.23; 95% CI: 1.78–2.78; *p* < 0.01; I^2^ = 16.9%; 18 trials; NNH: 11) (**Supplementary Fig. 22**), constipation (RR: 2.24; 95% CI: 1.64–3.04; *p* < 0.01; *I*^2^ = 38.3%; 18 trials; NNH: 14) (**Supplementary Fig. 23**), nervousness (RR: 2.20; 95% CI: 1.43–3.40; *p* < 0.01; *I*^2^ = 33.4%; 11 trials; NNH: 15) (**Supplementary Fig. 24**), insomnia (RR: 1.73; 95% CI: 1.37–2.19; *p* < 0.01; *I*^2^ = 26.9%; 19 trials; NNH: 19) (**Supplementary Fig. 25**), asthenia (RR: 1.78; 95% CI: 1.30–2.43; *p* < 0.01; *I*^2^ = 19.7%; 16 trials; NNH: 27) (**Supplementary Fig. 26**), tremor (RR: 2.30; 95% CI: 1.22–4.32; *p* = 0.01; *I*^2^ = 37.0%; 11 trials; NNH: 29) (**Supplementary Fig. 27**) and decreased appetite (RR: 2.52; 95% CI: 1.04–6.09; *p* < 0.01; *I*^2^ = 1.0%; 3 trials; NNH: 47) (**Supplementary Fig. 28**). The remaining meta-analyses showed no evidence of differences (**Supplementary Table 6, Supplementary Fig. 29–64**).

### Exploratory outcomes and sensitivity analyses

#### HDRS-17

Only two trials reported results on HDRS-17 (Higuchi *et al.*, 2016; Learned *et al.*, 2012). Both trials only assessed outcomes at the end of the treatment period, i.e. 8–10 weeks after randomisation. Meta-analysis showed evidence of a beneficial effect of venlafaxine (mean difference (MD): −1.50 points; 95% CI: −2.48 to −0.53; *p* < 0.01; 2 trials) (**Supplementary Fig. 65**), however, the effect size was below proposed minimal important differences (Jakobsen *et al.*, 2020). Visual inspection of the forest plot and statistical tests (*I*^2^ = 2.5%) indicated no clear signs of heterogeneity. This outcome result was assessed as overall high risk of bias.

#### Suicidal ideation

Four trials reported results on suicidal ideation (Hewett *et al.*, 2010; Higuchi *et al.*, 2016; Sheehan *et al.*, 2009; Wyeth-Ayerst Research, 1996). All trials only assessed outcomes at the end of the treatment period, i.e. 6–8 weeks after randomisation. A total of 91/812 (11.2%) experimental participants had suicidal ideation compared with 42/548 (7.7%) control participants. Meta-analysis showed no evidence of a difference between venlafaxine and placebo (RR: 1.13; 95% CI: 0.74–1.73; *p* = 0.58; 4 trials) (**Supplementary Fig. 66**). Visual inspection of the forest plot and statistical tests (*I*^2^ = 15.8%) indicated no clear signs of heterogeneity. This outcome result was assessed as overall high risk of bias.

#### MADRS, BDI and HDRS-6

Nine trials reported results on MADRS (Alvarez *et al.*, 2012; Guelfi *et al.*, 1995; Hewett *et al.*, 2010; Higuchi *et al.*, 2016; Khan *et al.*, 1991; Mendels *et al.*, 1993; Schweizer *et al.*, 1994; Sheehan *et al.*, 2009; Thase, 1997; Wyeth-Ayerst Research, 1996). No trials reported results on BDI or HDRS-6. All trials only assessed outcomes at the end of the treatment period, i.e. 4–8 weeks after randomisation. Meta-analysis showed evidence of a beneficial effect of venlafaxine versus placebo (MD: −4.03 MADRS points; 95% CI: −5.30 to −2.75; *p* < 0.01; 9 trials) (**Supplementary Fig. 67**), however, the effect size was below proposed minimal important differences (Leucht *et al.*, 2017). Visual inspection of the forest plot and statistical tests (*I*^2^ = 60.0%) indicated heterogeneity. This outcome result was assessed as overall high risk of bias.

#### Remaining results

It was not possible to analyse the remaining exploratory outcomes due to a lack of relevant data. We performed all meta-analyses as both fixed-effect and random-effects meta-analyses and reported the most conservative results as the main results. For the less conservative results, please see **Supplementary Fig. 68–131**.

## Discussion

We conducted a systematic review assessing the risks of adverse events with venlafaxine for adults with major depressive disorder. We included 28 trials randomising 6,253 participants to venlafaxine versus placebo. All results were at high risk of bias, and the certainty of the evidence was very low. Data were limited to a maximum of 12 weeks after randomisation. Meta-analysis and Trial Sequential Analysis showed insufficient information to assess the effects of venlafaxine on risks of suicides or suicide attempts. Meta-analysis showed evidence of a harmful effect of venlafaxine on serious adverse events, mainly due to higher risks of sexual dysfunction and anorexia. Meta-analysis showed that venlafaxine increased the risk of several non-serious adverse events: nausea, dry mouth, dizziness, sweating, somnolence, constipation, nervousness, insomnia, asthenia, tremor and decreased appetite. Our results contribute important information on the risks of adverse events, since previous reviews have not systematically assessed all adverse effects (Cipriani *et al.*, 2018; Schueler *et al.*, 2011). We confirmed the previously shown statistically significant effects of antidepressants on depressive symptom rating scales (Cipriani *et al.*, 2018; Hengartner and Ploderl, 2022; Jakobsen *et al.*, 2020; Kamp *et al.*, 2024; Schueler *et al.*, 2011), but with effect sizes below proposed minimal important differences (3 HDRS points and 7–9 MADRS points) (Jakobsen *et al.*, 2020; Leucht *et al.*, 2017).

Our systematic review has several strengths. Previously, understanding of the adverse effects of venlafaxine has primarily been based on results of non-randomised studies. Accordingly, this is the first systematic review to assess all adverse effects of venlafaxine in adults with major depressive disorder. Data on adverse effects are essential for enabling patients and clinicians to make informed decisions about the use of any treatment. Our predefined methodology was based on the Cochrane Handbook for Systematic Reviews of Interventions (Higgins *et al.*, 2022), PRISMA (Liberati *et al.*, 2009), Trial Sequential Analysis (Copenhagen Trial Unit – Centre for Clinical Intervention Research, 2023; Thorlund *et al.*, 2017), the eight-step procedure by Jakobsen *et al.* (2014b), the GRADE approach (Guyatt *et al.*, 2011), and risks of systematic and random errors, external validity, publication bias and heterogeneity were taken into account. Furthermore, unpublished data were included in the analyses to increase the validity of our results (Golder *et al.*, 2016; Higgins *et al.*, 2022; Maund *et al.*, 2014; Tang *et al.*, 2015).

Our systematic review also has limitations. First, the included trials only reported results at the end of treatment at a maximum of 12 weeks, so the long-term effects of venlafaxine are unknown. There is a need for trials with long-term follow-up to assess the benefits and harms since, for example, half of patients on antidepressants in the United Kingdom and 70% of patients in the USA have used them for more than 2 years (Johnson *et al.*, 2012; Mojtabai and Olfson, 2014). This is particularly pertinent for medications that are associated with tolerance and withdrawal effects, which tend to show diminishing beneficial effects over time (Kinrys *et al.*, 2019). Second, all included trials were assessed at overall high risk of bias mainly driven by risks of bias due to missing data, lack of information on blinding (Juul *et al.*, 2021a), risk of unblinding due to adverse events, inappropriate analysis methods and poor reporting. The reporting and assessment of adverse events were especially inadequate, and a major limitation is that data on adverse events are rarely collected systematically, even in randomised trials. It usually depends on spontaneous reporting, and our analyses are likely to underestimate serious adverse event incidences. Third, the certainty of the evidence was very low for all outcome results. It is, therefore, likely that trials conducted with higher methodological quality will show different results and possibly reveal more severe adverse events. Fourth, only six of the included trials reported on suicides or suicide attempts, and there was not enough information to confirm or reject the effects of venlafaxine on suicides or suicide attempts. This is particularly problematic since major depressive disorder is associated with increased risks of suicidal behaviour (Chen and Dilsaver, 1996; Kessler *et al.*, 1999; Qin, 2011), and because antidepressants have been linked with an increased risk of suicidal behaviour, especially in young people (Sharma *et al.*, 2016). There is a need for larger trials at low risk of bias to assess the risks of suicides and suicide attempts. Fifth, only eight trials had publicly available protocols or trial registrations, making it difficult to assess risks of bias, particularly whether selective reporting had occurred. Sixth, we included three primary outcomes, which increased the risk of type I errors. To control the risks of random errors, we adjusted our threshold for significance according to the number of primary outcomes, but we did not adjust the thresholds for significance according to the total number of comparisons, including exploratory outcomes, subgroup analyses and sensitivity analyses. Seventh, trials comparing antidepressants with ‘active placebo’ indicate that the beneficial effects may be inflated due to the unblinding effects of the drug when compared with an inert placebo (Moncrieff *et al.*, 2004). We did not identify any trials comparing venlafaxine to ‘active placebo’, which leaves open the possibility that efficacy results are impacted by unblinding caused by adverse effects or other noticeable changes produced by medication, as shown in trials of other antidepressants (Jureidini *et al.*, 2024). Lastly, withdrawal effects of venlafaxine were only assessed in two of the included trials (Wyeth-Ayerst, 1991b, 1991c), emphasising the lack of data on a harm of treatment that has increasingly been highlighted (Davies and Read, 2019) and is particularly relevant to venlafaxine, given its short half-life (Campagne, 2005; Gastaldon *et al.*, 2022). These limitations need to be considered when interpreting our results.

## Conclusions

Short-term results show that venlafaxine has uncertain effects on the risks of suicides but increases the risks of serious adverse events (especially sexual dysfunction and anorexia) and many non-serious adverse events. The long-term effects of venlafaxine for major depressive disorder are unknown. More information on adverse effects is critical if patients and clinicians should make informed decisions about the costs and benefits of using venlafaxine. It is a particular cause for concern that there are no data on the long-term adverse effects of venlafaxine given that so many people use these drugs for several years.

## Differences between the protocol and the review

There were no differences between the protocol and the review.

## Supporting information

Kamp et al. supplementary material Kamp et al. supplementary material

## Data Availability

All data generated or analysed during this study are included in this article and its supplementary material files.

## Supplementary material

The supplementary material for this article can be found at https://doi.org/10.1017/S2045796024000520.
