# Supplementary material for: The risks of adverse events with venlafaxine for adults with major depressive disorder: a systematic review of randomised clinical trials with meta-analysis and Trial Sequential Analysis
Source: Epidemiol Psychiatr Sci. 2024 Oct 23;33:e51. doi: 10.1017/S2045796024000520 (PMC11561525; doi:10.1017/S2045796024000520)
Supplement: Kamp et al. supplementary material [file S2045796024000520sup001.pdf]

Supplementary material for:

The risks of adverse events with venlafaxine for adults with major depressive disorder: a systematic review of randomised clinical trials with meta-analysis and Trial Sequential Analysis

CB Kamp, JJ Petersen, P Faltermeier, S Juul, F Siddiqui, J Moncrieff, MA Horowitz, MP Hengartner, I Kirsch, C Gluud, JC Jakobsen

Table of Contents

Supplementary Figure 1: Risk of Bias 2 assessments 7

Supplementary Figure 2: Subgroup analysis of placebo washout on suicides or suicide attempts 8

Supplementary Figure 3: Trial Sequential Analysis of venlafaxine versus placebo on serious adverse events 9

Supplementary Figure 4: Subgroup analysis of placebo washout on serious adverse events 10

Supplementary Figure 5: Meta-analysis of venlafaxine versus placebo on sexual dysfunction 11

Supplementary Figure 6: Meta-analysis of venlafaxine versus placebo on anorexia 12

Supplementary Figure 7: Meta-analysis of venlafaxine versus placebo on anxiety 13

Supplementary Figure 8: Meta-analysis of venlafaxine versus placebo on worsening of depression 14

Supplementary Figure 9: Meta-analysis of venlafaxine versus placebo on hypertension 15

Supplementary Figure 10: Meta-analysis of venlafaxine versus placebo on hypotension 16

Supplementary Figure 11: Meta-analysis of venlafaxine versus placebo on discontinuation symptoms 17

Supplementary Figure 12: Meta-analysis of venlafaxine versus placebo on fall 18

Supplementary Figure 13: Meta-analysis of venlafaxine versus placebo on intentional overdose 19

Supplementary Figure 14: Meta-analysis of venlafaxine versus placebo on QTc 20

Supplementary Figure 15: Meta-analysis of venlafaxine versus placebo on syncope 21

Supplementary Figure 16: Trial Sequential Analysis of venlafaxine versus placebo on non-serious adverse events 22

Supplementary Figure 17: Subgroup analysis of placebo washout on non-serious adverse events 23

Supplementary Figure 18: Meta-analysis of venlafaxine versus placebo on nausea 24

Supplementary Figure 19: Meta-analysis of venlafaxine versus placebo on dry mouth 25

Supplementary Figure 20: Meta-analysis of venlafaxine versus placebo on dizziness 26

Supplementary Figure 21: Meta-analysis of venlafaxine versus placebo on sweating 27

Supplementary Figure 22: Meta-analysis of venlafaxine versus placebo on somnolence 28

Supplementary Figure 23: Meta-analysis of venlafaxine versus placebo on constipation 29

Supplementary Figure 24: Meta-analysis of venlafaxine versus placebo on nervousness 30

Supplementary Figure 25: Meta-analysis of venlafaxine versus placebo on insomnia 31

Supplementary Figure 26: Meta-analysis of venlafaxine versus placebo on asthenia 32

Supplementary Figure 27: Meta-analysis of venlafaxine versus placebo on tremor 33

Supplementary Figure 28: Meta-analysis of venlafaxine versus placebo on decreased appetite 34

Supplementary Figure 29: Meta-analysis of venlafaxine versus placebo on abdominal pain 35

Supplementary Figure 30: Meta-analysis of venlafaxine versus placebo on abnormal dreams 36

Supplementary Figure 31: Meta-analysis of venlafaxine versus placebo on abnormal vision 37

Supplementary Figure 32: Meta-analysis of venlafaxine versus placebo on agitation 38

Supplementary Figure 33: Meta-analysis of venlafaxine versus placebo on back pain 39

Supplementary Figure 34: Meta-analysis of venlafaxine versus placebo on increased blood pressure 40

Supplementary Figure 35: Meta-analysis of venlafaxine versus placebo on coughing 41

Supplementary Figure 36: Meta-analysis of venlafaxine versus placebo on diarrhoea 42
Supplementary Figure 37: Meta-analysis of venlafaxine versus placebo on dyspepsia 43
Supplementary Figure 38: Meta-analysis of venlafaxine versus placebo on flatulence 44
Supplementary Figure 39: Meta-analysis of venlafaxine versus placebo on headache 45
Supplementary Figure 40: Meta-analysis of venlafaxine versus placebo on infection 46
Supplementary Figure 41: Meta-analysis of venlafaxine versus placebo on influenza 47
Supplementary Figure 42: Meta-analysis of venlafaxine versus placebo on malaise 48
Supplementary Figure 43: Meta-analysis of venlafaxine versus placebo on nasopharyngitis 49
Supplementary Figure 44: Meta-analysis of venlafaxine versus placebo on palpitations 50
Supplementary Figure 45: Meta-analysis of venlafaxine versus placebo on rhinitis 51
Supplementary Figure 46: Meta-analysis of venlafaxine versus placebo on tachycardia 52
Supplementary Figure 47: Meta-analysis of venlafaxine versus placebo on urinary frequency 53
Supplementary Figure 48: Meta-analysis of venlafaxine versus placebo on vertigo 54
Supplementary Figure 49: Meta-analysis of venlafaxine versus placebo on vomiting 55
Supplementary Figure 50: Meta-analysis of venlafaxine versus placebo on weight loss 56
Supplementary Figure 51: Meta-analysis of venlafaxine versus placebo on abnormality of accommodation 57
Supplementary Figure 52: Meta-analysis of venlafaxine versus placebo on pruritis 58
Supplementary Figure 53: Meta-analysis of venlafaxine versus placebo on vasodilation 59
Supplementary Figure 54: Meta-analysis of venlafaxine versus placebo on neck pain 60
Supplementary Figure 55: Meta-analysis of venlafaxine versus placebo on pain 61
Supplementary Figure 56: Meta-analysis of venlafaxine versus placebo on increased salivation 62
Supplementary Figure 57: Meta-analysis of venlafaxine versus placebo on tongue discolouration 63
Supplementary Figure 58: Meta-analysis of venlafaxine versus placebo on hypochromic anaemia 64
Supplementary Figure 59: Meta-analysis of venlafaxine versus placebo on hypercholesterolemia 65
Supplementary Figure 60: Meta-analysis of venlafaxine versus placebo on bronchitis 66
Supplementary Figure 61: Meta-analysis of venlafaxine versus placebo on pharyngitis 67
Supplementary Figure 62: Meta-analysis of venlafaxine versus placebo on urinary tract infection 68
Supplementary Figure 63: Meta-analysis of venlafaxine versus placebo on urine abnormality 69
Supplementary Figure 64: Meta-analysis of venlafaxine versus placebo on taste alteration 70
Supplementary Figure 65: Meta-analysis of venlafaxine versus placebo on HDRS-17 71
Supplementary Figure 66: Meta-analysis of venlafaxine versus placebo on suicidal ideation 72
Supplementary Figure 67: Meta-analysis of venlafaxine versus placebo on MADRS 73
Supplementary Figure 68: Meta-analysis of venlafaxine versus placebo on suicides or suicide attempts (sensitivity analysis) 74
Supplementary Figure 69: Meta-analysis of venlafaxine versus placebo on serious adverse events (sensitivity analysis) 75
Supplementary Figure 70: Meta-analysis of venlafaxine versus placebo on sexual dysfunction (sensitivity analysis). 76

Supplementary Figure 71: Meta-analysis of venlafaxine versus placebo on anorexia (sensitivity analysis)................77

Supplementary Figure 72: Meta-analysis of venlafaxine versus placebo on anxiety (sensitivity analysis) 78

Supplementary Figure 73: Meta-analysis of venlafaxine versus placebo on worsening of depression (sensitivity analysis) 79

Supplementary Figure 74: Meta-analysis of venlafaxine versus placebo on hypertension (sensitivity analysis) 80

Supplementary Figure 75: Meta-analysis of venlafaxine versus placebo on hypotension (sensitivity analysis)...........81

Supplementary Figure 76: Meta-analysis of venlafaxine versus placebo on discontinuation symptoms (sensitivity analysis) 82

Supplementary Figure 77: Meta-analysis of venlafaxine versus placebo on fall (sensitivity analysis) 83

Supplementary Figure 78: Meta-analysis of venlafaxine versus placebo on intentional overdose (sensitivity analysis) 84

Supplementary Figure 79: Meta-analysis of venlafaxine versus placebo on QTc (sensitivity analysis) 85

Supplementary Figure 80: Meta-analysis of venlafaxine versus placebo on syncope (sensitivity analysis) 86

Supplementary Figure 81: Meta-analysis of venlafaxine versus placebo on non-serious adverse events (sensitivity analysis) 87

Supplementary Figure 82: Meta-analysis of venlafaxine versus placebo on nausea (sensitivity analysis)..................88

Supplementary Figure 83: Meta-analysis of venlafaxine versus placebo on dry mouth (sensitivity analysis) 89

Supplementary Figure 84: Meta-analysis of venlafaxine versus placebo on dizziness (sensitivity analysis)................90

Supplementary Figure 85: Meta-analysis of venlafaxine versus placebo on sweating (sensitivity analysis)................91

Supplementary Figure 86: Meta-analysis of venlafaxine versus placebo on somnolence (sensitivity analysis)...........92

Supplementary Figure 87: Meta-analysis of venlafaxine versus placebo on constipation (sensitivity analysis) 93

Supplementary Figure 88: Meta-analysis of venlafaxine versus placebo on nervousness (sensitivity analysis) 94

Supplementary Figure 89: Meta-analysis of venlafaxine versus placebo on insomnia (sensitivity analysis) 95

Supplementary Figure 90: Meta-analysis of venlafaxine versus placebo on asthenia (sensitivity analysis) 96

Supplementary Figure 91: Meta-analysis of venlafaxine versus placebo on tremor (sensitivity analysis)..................97

Supplementary Figure 92: Meta-analysis of venlafaxine versus placebo on decreased appetite (sensitivity analysis)98

Supplementary Figure 93: Meta-analysis of venlafaxine versus placebo on abdominal pain (sensitivity analysis).....99

Supplementary Figure 94: Meta-analysis of venlafaxine versus placebo on abnormal dreams (sensitivity analysis)100

Supplementary Figure 95: Meta-analysis of venlafaxine versus placebo on abnormal vision (sensitivity analysis) . 101

Supplementary Figure 96: Meta-analysis of venlafaxine versus placebo on agitation (sensitivity analysis)............ 102

Supplementary Figure 97: Meta-analysis of venlafaxine versus placebo on back pain (sensitivity analysis)............ 103

Supplementary Figure 98: Meta-analysis of venlafaxine versus placebo on increased blood pressure (sensitivity analysis) 104

Supplementary Figure 99: Meta-analysis of venlafaxine versus placebo on coughing (sensitivity analysis) 105

Supplementary Figure 100: Meta-analysis of venlafaxine versus placebo on diarrhoea (sensitivity analysis) 106

Supplementary Figure 101: Meta-analysis of venlafaxine versus placebo on dyspepsia (sensitivity analysis) 107

Supplementary Figure 102: Meta-analysis of venlafaxine versus placebo on flatulence (sensitivity analysis) 108

Supplementary Figure 103: Meta-analysis of venlafaxine versus placebo on headache (sensitivity analysis) 109

Supplementary Figure 104: Meta-analysis of venlafaxine versus placebo on infection (sensitivity analysis) 110

Supplementary Figure 105: Meta-analysis of venlafaxine versus placebo on influenza (sensitivity analysis)........... 111

Supplementary Figure 106: Meta-analysis of venlafaxine versus placebo on malaise (sensitivity analysis)............ 112

Supplementary Figure 107: Meta-analysis of venlafaxine versus placebo on nasopharyngitis (sensitivity analysis) 113

Supplementary Figure 108: Meta-analysis of venlafaxine versus placebo on palpitations (sensitivity analysis)...... 114

Supplementary Figure 109: Meta-analysis of venlafaxine versus placebo on rhinitis (sensitivity analysis) 115

Supplementary Figure 110: Meta-analysis of venlafaxine versus placebo on tachycardia (sensitivity analysis) 116

Supplementary Figure 111: Meta-analysis of venlafaxine versus placebo on urinary frequency (sensitivity analysis) 117

Supplementary Figure 112: Meta-analysis of venlafaxine versus placebo on vertigo (sensitivity analysis)............. 118

Supplementary Figure 113: Meta-analysis of venlafaxine versus placebo on vomiting (sensitivity analysis)........... 119

Supplementary Figure 114: Meta-analysis of venlafaxine versus placebo on weight loss (sensitivity analysis)....... 120

Supplementary Figure 115: Meta-analysis of venlafaxine versus placebo on abnormality of accommodation (sensitivity analysis) 121

Supplementary Figure 116: Meta-analysis of venlafaxine versus placebo on pruritis (sensitivity analysis)............. 122

Supplementary Figure 117: Meta-analysis of venlafaxine versus placebo on vasodilation (sensitivity analysis)...... 123

Supplementary Figure 118: Meta-analysis of venlafaxine versus placebo on neck pain (sensitivity analysis).......... 124

Supplementary Figure 119: Meta-analysis of venlafaxine versus placebo on pain (sensitivity analysis) 125

Supplementary Figure 120: Meta-analysis of venlafaxine versus placebo on increased salivation (sensitivity analysis) 126

Supplementary Figure 121: Meta-analysis of venlafaxine versus placebo on tongue discolouration (sensitivity analysis) 127

Supplementary Figure 122: Meta-analysis of venlafaxine versus placebo on hypochromic anaemia (sensitivity analysis) 128

Supplementary Figure 123: Meta-analysis of venlafaxine versus placebo on hypercholesterolemia (sensitivity analysis) 129

Supplementary Figure 124: Meta-analysis of venlafaxine versus placebo on bronchitis (sensitivity analysis) 130

Supplementary Figure 125: Meta-analysis of venlafaxine versus placebo on pharyngitis (sensitivity analysis)....... 131

Supplementary Figure 126: Meta-analysis of venlafaxine versus placebo on urinary tract infection (sensitivity analysis) 132

Supplementary Figure 127: Meta-analysis of venlafaxine versus placebo on urine abnormality (sensitivity analysis) 133

Supplementary Figure 128: Meta-analysis of venlafaxine versus placebo on taste alteration (sensitivity analysis) 134

Supplementary Figure 129: Meta-analysis of venlafaxine versus placebo on HDRS-17 (sensitivity analysis)........... 135

*Supplementary Figure 130: Meta-analysis of venlafaxine versus placebo on suicidal ideation (sensitivity analysis)*136

Supplementary Figure 131: Meta-analysis of venlafaxine versus placebo on MADRS (sensitivity analysis)............ 137

Supplementary Table 1: PRISMA checklist.. 138

Supplementary Table 2: Search strategies 141

Supplementary Table 3: Characteristics of the included trials.. 144

Supplementary Table 4: Summary of Findings *147*
Supplementary Table 5: RoB2 table with explanations *148*
Supplementary Table 6: Individual serious adverse events *152*
Supplementary Table 7: Serious adverse events in the included trials *153*
Supplementary Table 8: Individual non-serious adverse events *154*

Supplementary Figure 1: Risk of Bias 2 assessments

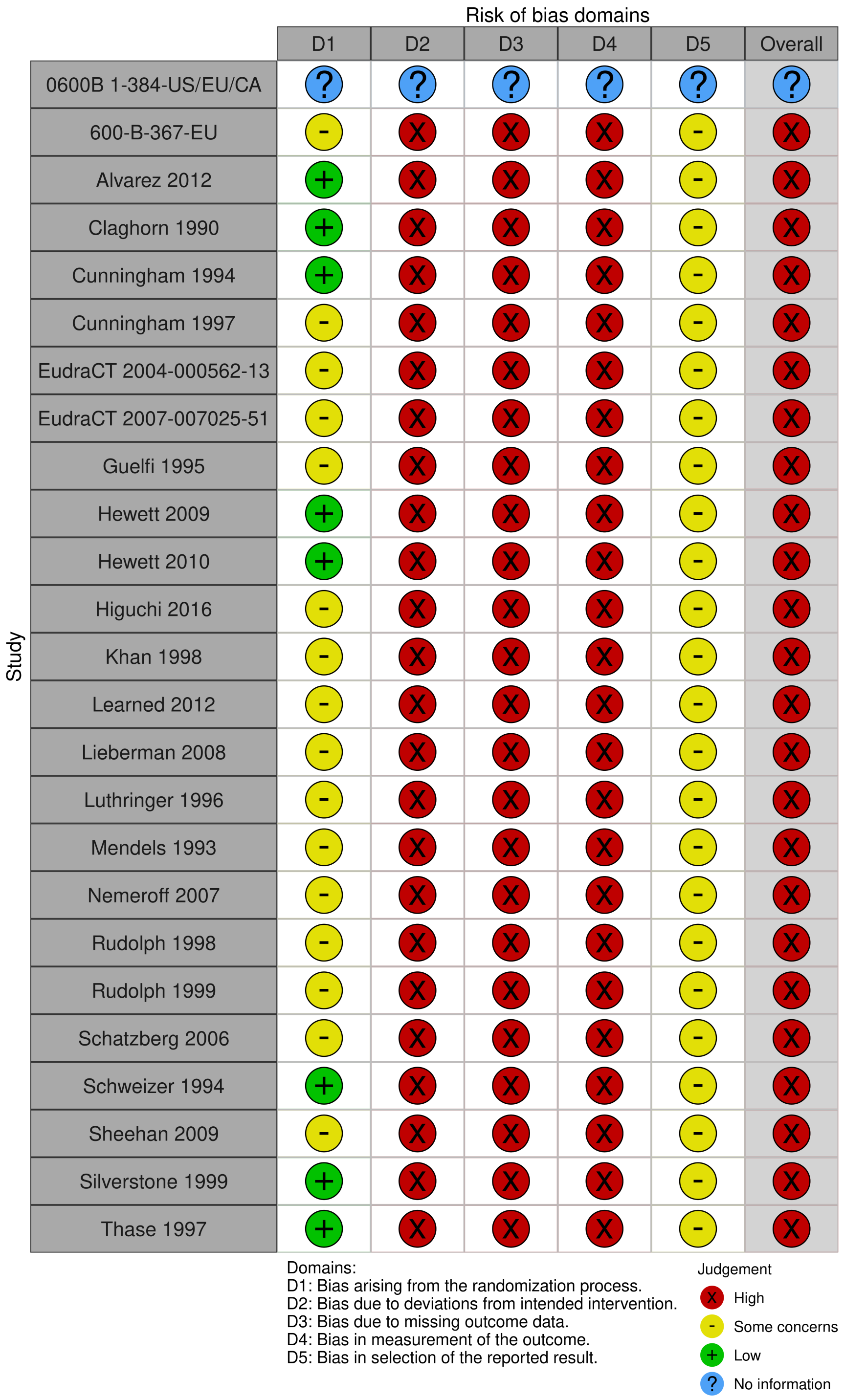

Based on assessments of the primary outcomes.

Supplementary Figure 2: Subgroup analysis of placebo washout on suicides or suicide attempts

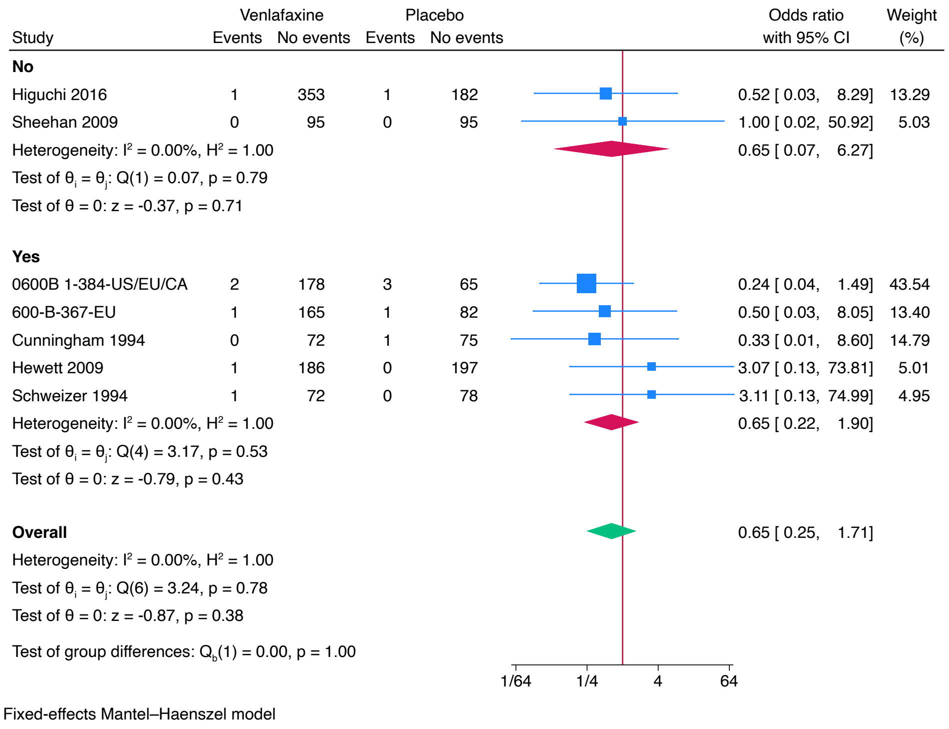

Supplementary Figure 3: Trial Sequential Analysis of venlafaxine versus placebo on serious adverse events

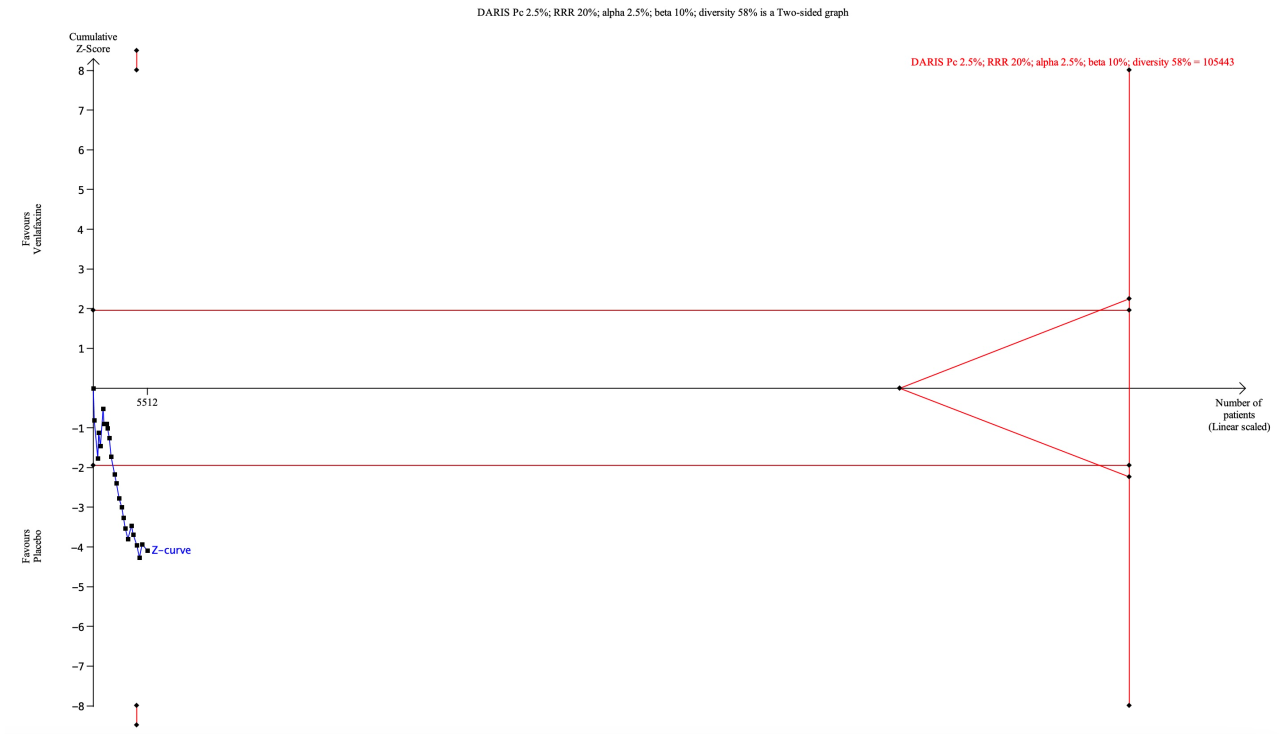

Supplementary Figure 4: Subgroup analysis of placebo washout on serious adverse events

Study	Venlafaxine Events	Venlafaxine No events	Placebo Events	Placebo No events	Risk ratio with 95% CI	Weight (%)
**No**						
Alvarez 2012	14	99	2	103	6.50 [1.51, 27.94]	4.72
EudraCT 2004-000562-13	6	121	2	118	2.83 [0.58, 13.77]	4.37
EudraCT 2007-007025-51	0	7	0	7	1.00 [0.02, 44.50]	1.29
Hewett 2009	6	181	9	188	0.70 [0.25, 1.93]	6.19
Hewett 2010	9	189	2	185	4.25 [0.93, 19.41]	4.54
Higuchi 2016	7	347	1	182	3.62 [0.45, 29.19]	3.19
Learned 2012 (study 1)	3	130	5	121	0.57 [0.14, 2.33]	4.86
Lieberman 2008 (225 mg)	18	99	1	122	18.92 [2.57, 139.50]	3.37
Sheehan 2009	12	83	4	91	3.00 [1.00, 8.97]	5.90
Heterogeneity: $\tau^2 = 0.75$, $I^2 = 56.12\%$, $H^2 = 2.28$					2.54 [1.16, 5.57]	
Test of $\theta_i = \theta_j$: Q(8) = 16.51, p = 0.04						
Test of $\theta = 0$: z = 2.33, p = 0.02						
**Yes**						
0600B 1-384-US/EU/CA	1	179	3	65	0.13 [0.01, 1.19]	2.90
600-B-367-EU	10	155	4	79	1.26 [0.41, 3.89]	5.78
Claghorn 1990	2	77	0	80	5.06 [0.25, 103.80]	1.88
Cunningham 1994	4	68	4	72	1.06 [0.27, 4.06]	5.06
Cunningham 1997 (IR)	6	90	2	48	1.56 [0.33, 7.46]	4.42
Cunningham 1997 (XR)	10	87	2	48	2.58 [0.59, 11.31]	4.66
Guelfi 1995	4	42	0	47	9.19 [0.51, 166.04]	2.01
Mendels 1993	13	221	1	77	4.33 [0.58, 32.59]	3.33
Nemeroff 2007	10	90	1	101	10.20 [1.33, 78.21]	3.29
Rudolph 1998	40	226	2	90	6.92 [1.71, 28.06]	4.90
Rudolph 1999	9	91	4	94	2.21 [0.70, 6.92]	5.73
Schatzberg 2006	9	93	1	95	8.47 [1.09, 65.61]	3.27
Schweizer 1994	3	70	1	77	3.21 [0.34, 30.13]	2.91
Silverstone 1999	13	115	3	116	4.03 [1.18, 13.79]	5.44
Thase 1997	15	80	4	98	4.03 [1.39, 11.70]	6.00
Heterogeneity: $\tau^2 = 0.64$, $I^2 = 50.47\%$, $H^2 = 2.02$					2.75 [1.52, 4.98]	
Test of $\theta_i = \theta_j$: Q(14) = 17.97, p = 0.21						
Test of $\theta = 0$: z = 3.34, p = 0.00						
**Overall**					2.66 [1.67, 4.25]	
Heterogeneity: $\tau^2 = 0.65$, $I^2 = 52.00\%$, $H^2 = 2.08$						
Test of $\theta_i = \theta_j$: Q(23) = 34.88, p = 0.05						
Test of $\theta = 0$: z = 4.10, p = 0.00						
Test of group differences: $Q_b(1) = 0.03$, p = 0.87						

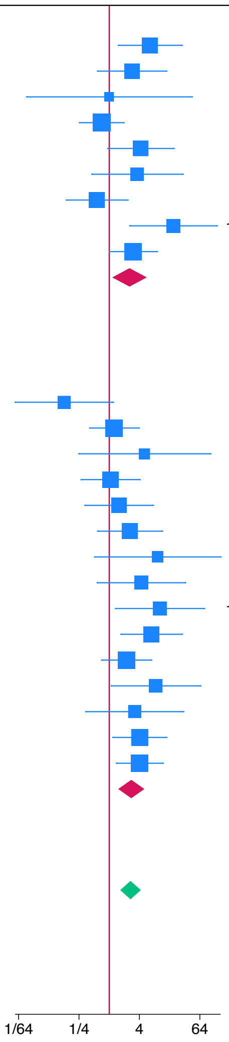

Random-effects Sidik–Jonkman model

Supplementary Figure 5: Meta-analysis of venlafaxine versus placebo on sexual dysfunction

Study	Venlafaxine Events	Venlafaxine No events	Placebo Events	Placebo No events	Risk ratio with 95% CI	Weight (%)
600-B-367-EU	2	52	0	26	2.45 [0.12, 49.36]	6.41
Alvarez 2012	14	99	2	103	6.50 [1.51, 27.94]	26.20
Cunningham 1997 (IR)	2	29	0	20	3.28 [0.17, 64.99]	6.48
Cunningham 1997 (XR)	10	27	0	21	12.16 [0.75, 197.55]	7.42
EudraCT 2004-000562-13	2	33	0	40	5.69 [0.28, 114.74]	6.40
Lieberman 2008 (225 mg)	4	32	1	44	5.00 [0.58, 42.80]	12.39
Mendels 1993	11	223	0	78	7.73 [0.46, 129.71]	7.25
Schatzberg 2006	9	93	1	95	8.47 [1.09, 65.61]	13.60
Thase 1997	8	27	1	40	9.37 [1.23, 71.31]	13.84
**Overall**					6.49 [3.02, 13.93]	

Heterogeneity: $\tau^2 = 0.03$, $I^2 = 1.94\%$, $H^2 = 1.02$

Test of $\theta_i = \theta_j$: Q(8) = 1.07, p = 1.00

Test of $\theta = 0$: z = 4.79, p = 0.00

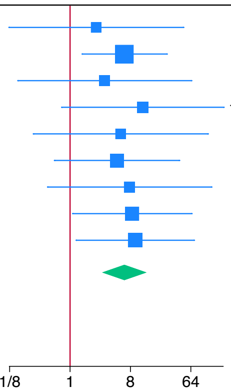

Random-effects Sidik–Jonkman model

Supplementary Figure 6: Meta-analysis of venlafaxine versus placebo on anorexia

Study	Venlafaxine Events	Venlafaxine No events	Placebo Events	Placebo No events	Risk ratio with 95% CI	Weight (%)
600-B-367-EU	2	163	2	81	0.50 [0.07, 3.51]	6.95
Cunningham 1997 (IR)	6	90	2	48	1.56 [0.33, 7.46]	9.21
Cunningham 1997 (XR)	10	87	2	48	2.58 [0.59, 11.31]	9.83
EudraCT 2004-000562-13	6	121	2	118	2.83 [0.58, 13.77]	9.09
Hewett 2010	9	189	2	185	4.25 [0.93, 19.41]	9.53
Lieberman 2008 (225 mg)	18	99	1	122	18.92 [2.57, 139.50]	6.68
Rudolph 1998	40	226	2	90	6.92 [1.71, 28.06]	10.45
Rudolph 1999	9	91	4	94	2.21 [0.70, 6.92]	12.76
Silverstone 1999	13	115	3	116	4.03 [1.18, 13.79]	11.94
Thase 1997	15	80	4	98	4.03 [1.39, 11.70]	13.55
**Overall**					3.23 [1.75, 5.97]	

Heterogeneity: $\tau^2 = 0.43$, $I^2 = 44.71\%$, $H^2 = 1.81$

Test of $\theta_i = \theta_j$: Q(9) = 9.45, p = 0.40

Test of $\theta = 0$: z = 3.75, p = 0.00

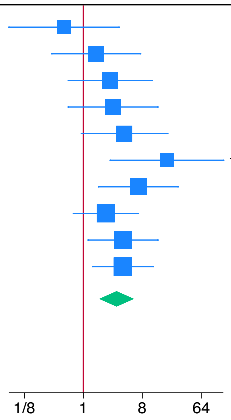

Random-effects Sidik–Jonkman model

Supplementary Figure 7: Meta-analysis of venlafaxine versus placebo on anxiety

Study	Venlafaxine Events	Venlafaxine No events	Placebo Events	Placebo No events	Risk ratio with 95% CI	Weight (%)
600-B-367-EU	2	163	2	81	0.50 [0.07, 3.51]	10.42
EudraCT 2004-000562-13	0	127	1	119	0.32 [0.01, 7.66]	5.72
Hewett 2009	6	181	9	188	0.70 [0.25, 1.93]	16.08
Learned 2012	3	130	5	121	0.57 [0.14, 2.33]	13.51
Mendels 1993	13	221	1	77	4.33 [0.58, 32.59]	10.04
Nemeroff 2007	10	90	1	101	10.20 [1.33, 78.21]	9.94
Rudolph 1998	10	57	0	26	8.34 [0.51, 137.36]	6.85
Schatzberg 2006	2	100	4	92	0.47 [0.09, 2.51]	11.90
Sheehan 2009	12	83	4	91	3.00 [1.00, 8.97]	15.55
**Overall**					1.40 [0.57, 3.44]	

Heterogeneity: $\tau^2 = 1.05$, $I^2 = 60.44\%$, $H^2 = 2.53$

Test of $\theta_i = \theta_j$: $Q(8) = 15.05$, $p = 0.06$

Test of $\theta = 0$: $z = 0.72$, $p = 0.47$

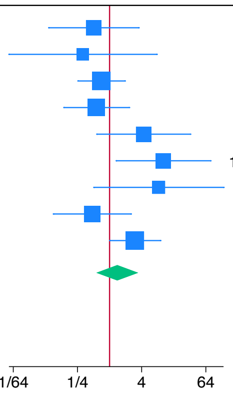

Random-effects Sidik–Jonkman model

Supplementary Figure 8: Meta-analysis of venlafaxine versus placebo on worsening of depression

Study	Venlafaxine Events	Venlafaxine No events	Placebo Events	Placebo No events	Risk ratio with 95% CI	Weight (%)
600-B-367-EU	4	161	0	83	4.55 [0.25, 83.60]	18.97
Cunningham 1994	0	72	1	75	0.35 [0.01, 8.49]	16.42
Hewett 2009	0	187	3	194	0.15 [0.01, 2.89]	18.50
Hewett 2010	1	197	1	186	0.94 [0.06, 14.99]	20.54
Sheehan 2009	1	94	2	93	0.50 [0.05, 5.42]	25.58
**Overall**					0.65 [0.16, 2.73]	

Heterogeneity: $\tau^2 = 0.60$, $I^2 = 22.54\%$, $H^2 = 1.29$

Test of $\theta_i = \theta_j$: $Q(4) = 2.92$, $p = 0.57$

Test of $\theta = 0$: $z = -0.58$, $p = 0.56$

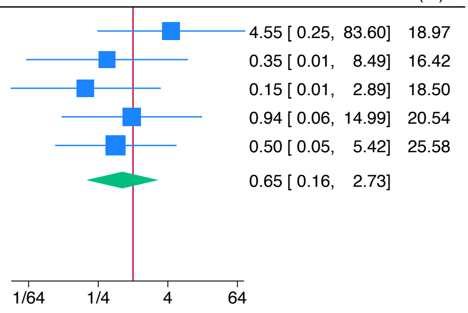

Random-effects Sidik–Jonkman model

Supplementary Figure 9: Meta-analysis of venlafaxine versus placebo on hypertension

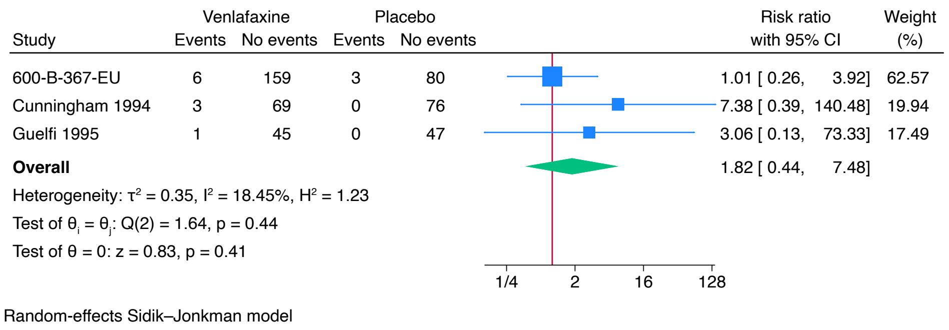

Supplementary Figure 10: Meta-analysis of venlafaxine versus placebo on hypotension

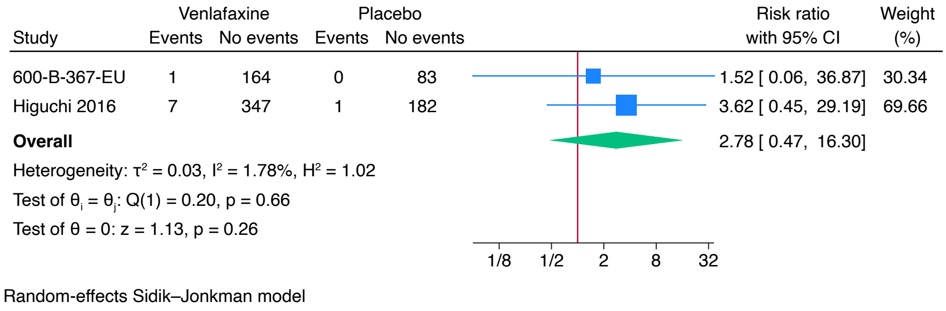

Supplementary Figure 11: Meta-analysis of venlafaxine versus placebo on discontinuation symptoms

Study	Venlafaxine Events	Venlafaxine No events	Placebo Events	Placebo No events	Risk ratio with 95% CI	Weight (%)
Claghorn 1990	1	78	0	80	3.04 [0.13, 73.46]	49.99
Schweizer 1994	1	72	0	78	3.20 [0.13, 77.39]	50.01
**Overall**					3.12 [0.33, 29.66]	

Heterogeneity: $\tau^2 = 0.00$, $I^2 = 0.00\%$, $H^2 = 1.00$

Test of $\theta_i = \theta_j$: Q(1) = 0.00, p = 0.98

Test of $\theta = 0$: z = 0.99, p = 0.32

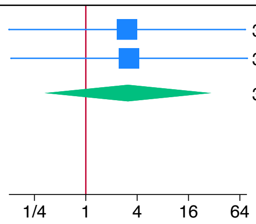

Random-effects Sidik–Jonkman model

Supplementary Figure 12: Meta-analysis of venlafaxine versus placebo on fall

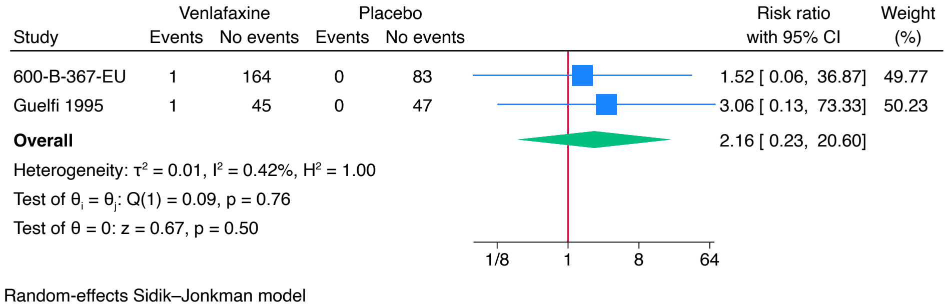

Supplementary Figure 13: Meta-analysis of venlafaxine versus placebo on intentional overdose

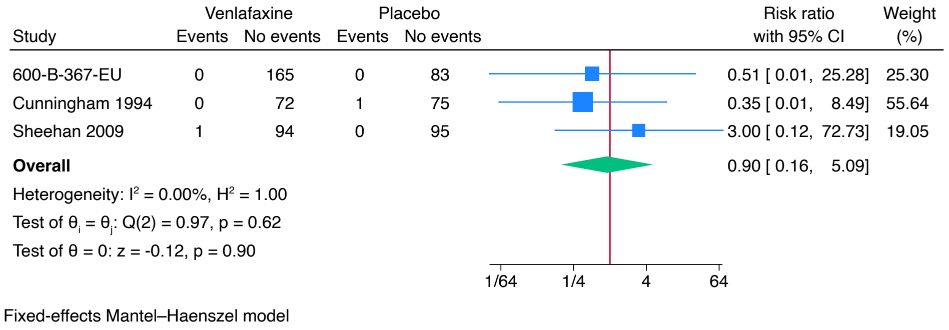

Supplementary Figure 14: Meta-analysis of venlafaxine versus placebo on QTc

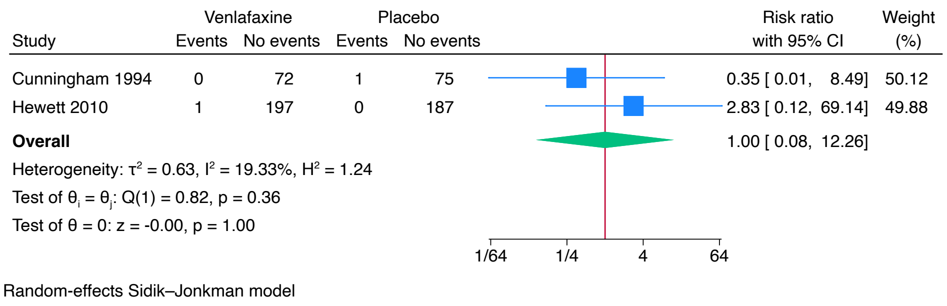

Supplementary Figure 15: Meta-analysis of venlafaxine versus placebo on syncope

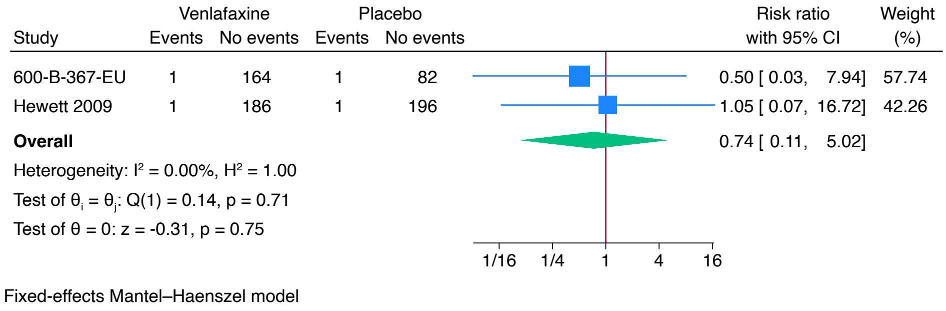

Supplementary Figure 16: Trial Sequential Analysis of venlafaxine versus placebo on non-serious adverse events

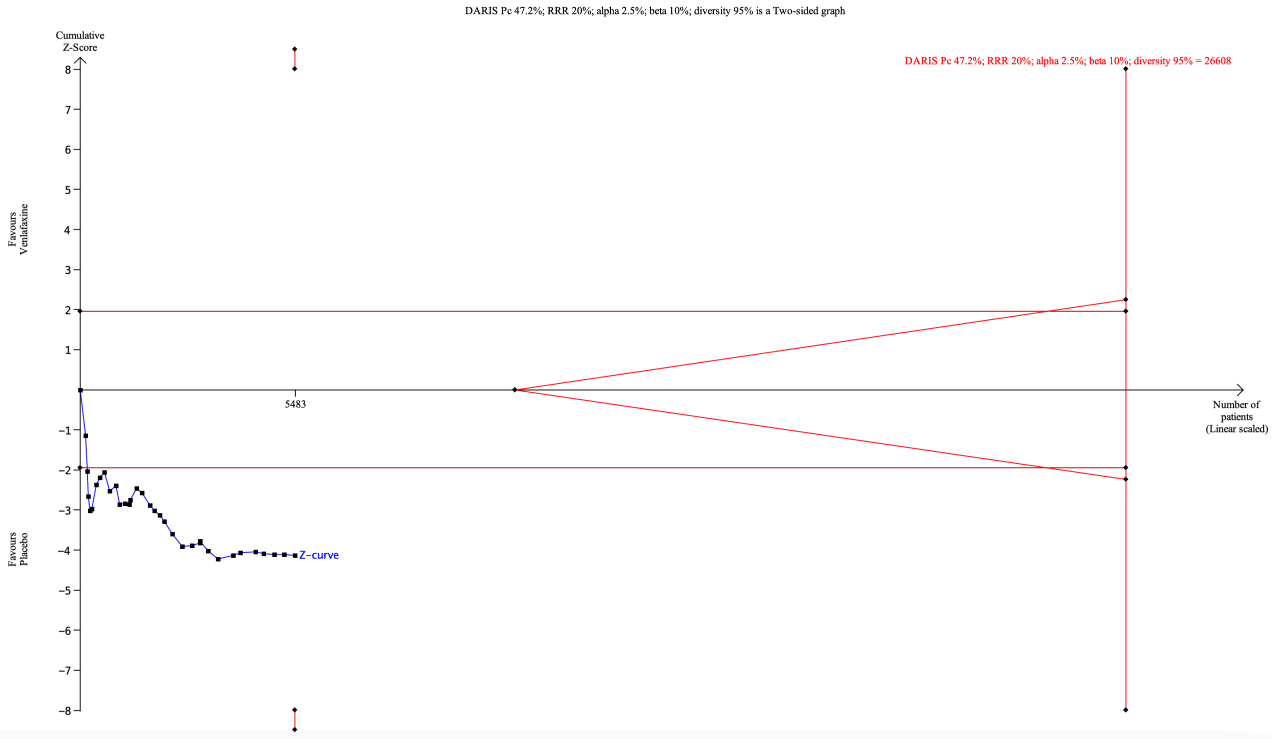

Supplementary Figure 17: Subgroup analysis of placebo washout on non-serious adverse events

Study	Venlafaxine Events	Venlafaxine No events	Placebo Events	Placebo No events	Risk ratio with 95% CI	Weight (%)
**No**						
Alvarez 2012	85	28	64	41	1.23 [1.02, 1.49]	3.85
EudraCT 2004-000562-13	92	35	77	43	1.13 [0.95, 1.34]	3.88
EudraCT 2007-007025-51	4	3	4	3	1.00 [0.40, 2.48]	1.84
Hewett 2009	93	94	95	102	1.03 [0.84, 1.26]	3.81
Hewett 2010	133	65	112	75	1.12 [0.96, 1.31]	3.91
Higuchi 2016 (fixed dose)	131	43	61	30	1.12 [0.95, 1.33]	3.88
Higuchi 2016 (flexible dose)	147	33	62	30	1.21 [1.03, 1.42]	3.90
Learned 2012 (study 1)	94	39	77	49	1.16 [0.97, 1.38]	3.87
Lieberman 2008 (225 mg)	27	90	16	109	1.80 [1.03, 3.17]	2.76
Sheehan 2009	87	8	85	10	1.02 [0.93, 1.12]	3.99
Heterogeneity: $\tau^2 = 0.01$, $I^2 = 52.13\%$, $H^2 = 2.09$					1.13 [1.04, 1.23]	
Test of $\theta_i = \theta_j$: Q(9) = 9.07, p = 0.43						
Test of $\theta = 0$: z = 2.86, p = 0.00						
**Yes**						
600-B-367-EU (75 mg)	56	27	25	16	1.11 [0.83, 1.47]	3.61
600-B-367-EU (150 mg)	53	29	25	17	1.09 [0.81, 1.46]	3.59
Claghorn 1990	72	7	59	21	1.24 [1.07, 1.43]	3.92
Cunningham 1994	71	1	56	20	1.34 [1.17, 1.53]	3.93
Cunningham 1997 (IR)	43	53	5	45	4.48 [1.89, 10.59]	1.95
Cunningham 1997 (XR)	44	53	5	45	4.54 [1.92, 10.72]	1.95
Guelfi 1995	36	10	22	25	1.67 [1.19, 2.35]	3.46
Khan 1991 (75 mg)	23	0	5	3	1.60 [0.95, 2.71]	2.89
Khan 1991 (225 mg)	22	0	6	3	1.51 [0.95, 2.38]	3.10
Khan 1991 (375 mg)	21	1	6	3	1.43 [0.89, 2.29]	3.06
Khan 1998 (75 mg)	24	72	2	31	4.12 [1.03, 16.52]	1.07
Khan 1998 (150 mg)	19	77	2	31	3.27 [0.80, 13.27]	1.05
Khan 1998 (200 mg)	26	68	2	30	4.43 [1.11, 17.61]	1.07
Luthringer 1996	5	7	6	6	0.83 [0.35, 2.00]	1.91
Mendels 1993 (25 mg)	20	59	7	19	0.94 [0.45, 1.97]	2.26
Mendels 1993 (50 - 75 mg)	19	57	7	19	0.93 [0.44, 1.95]	2.24
Mendels 1993 (150 - 200 mg)	17	52	7	19	0.92 [0.43, 1.95]	2.21
Nemeroff 2007	40	60	8	94	5.10 [2.51, 10.34]	2.34
Rudolph 1999	36	64	14	84	2.52 [1.45, 4.37]	2.81
Schatzberg 2006	94	8	83	13	1.07 [0.97, 1.17]	3.99
Schweizer 1991 (75, 225 and 375 mg)	24	20	4	12	2.18 [0.90, 5.32]	1.88
Schweizer 1994	63	10	64	14	1.05 [0.92, 1.21]	3.93
Silverstone 1999	49	79	20	99	2.28 [1.44, 3.59]	3.11
Thase 1997	34	61	18	84	2.03 [1.23, 3.34]	2.97
Heterogeneity: $\tau^2 = 0.23$, $I^2 = 91.19\%$, $H^2 = 11.36$					1.63 [1.30, 2.05]	
Test of $\theta_i = \theta_j$: Q(23) = 83.61, p = 0.00						
Test of $\theta = 0$: z = 4.18, p = 0.00						
**Overall**					1.43 [1.21, 1.69]	
Heterogeneity: $\tau^2 = 0.18$, $I^2 = 92.92\%$, $H^2 = 14.13$						
Test of $\theta_i = \theta_j$: Q(33) = 99.10, p = 0.00						
Test of $\theta = 0$: z = 4.21, p = 0.00						
Test of group differences: $Q_b(1) = 8.58$, p = 0.00						

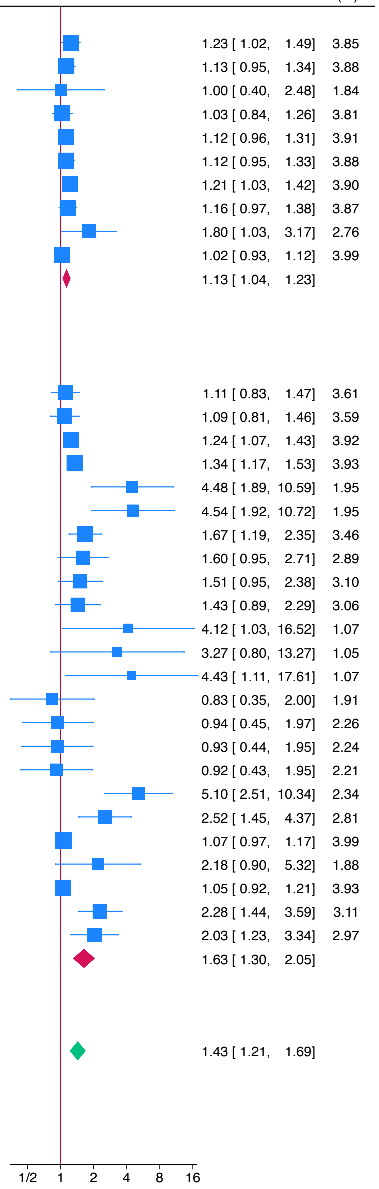

Random-effects Sidik–Jonkman model

Supplementary Figure 18: Meta-analysis of venlafaxine versus placebo on nausea

Graph 17/03/2024, 18.33

Study	Venlafaxine Events	Venlafaxine No events	Placebo Events	Placebo No events	Risk ratio with 95% CI	Weight (%)
600-B-367-EU (75 mg)	15	68	3	38	2.47 [0.76, 8.05]	1.88
600-B-367-EU (150 mg)	22	60	2	40	5.63 [1.39, 22.82]	1.44
Alvarez 2012	38	75	10	95	3.53 [1.85, 6.72]	4.05
Claghorn 1990	35	44	7	73	5.06 [2.39, 10.71]	3.45
Cunningham 1994	32	40	4	72	8.44 [3.14, 22.68]	2.44
Cunningham 1997 (IR)	43	53	5	45	4.48 [1.89, 10.59]	2.93
Cunningham 1997 (XR)	44	53	5	45	4.54 [1.92, 10.72]	2.94
EudraCT 2004-000562-13	27	100	14	106	1.82 [1.00, 3.30]	4.36
EudraCT 2007-007025-51	0	7	0	7	1.00 [0.02, 44.50]	0.23
Guelfi 1995	8	38	1	46	8.17 [1.06, 62.78]	0.75
Hewett 2009	36	151	21	176	1.81 [1.10, 2.98]	5.02
Hewett 2010	53	145	16	171	3.13 [1.86, 5.27]	4.86
Higuchi 2016 (fixed dose)	39	135	12	80	1.72 [0.95, 3.12]	4.35
Higuchi 2016 (flexible dose)	53	127	12	79	2.23 [1.26, 3.96]	4.50
Khan 1998 (75 mg)	8	88	1	32	2.75 [0.36, 21.17]	0.75
Khan 1998 (150 mg)	7	89	0	33	5.26 [0.31, 89.63]	0.41
Khan 1998 (200 mg)	16	78	0	32	11.46 [0.71, 185.79]	0.42
Learned 2012	29	104	14	112	1.96 [1.09, 3.54]	4.40
Lieberman 2008 (225 mg)	34	83	16	109	2.27 [1.33, 3.89]	4.74
Mendels 1993 (25 mg)	22	57	3	23	2.41 [0.79, 7.41]	2.04
Mendels 1993 (50 - 75 mg)	19	57	2	24	3.25 [0.81, 13.01]	1.46
Mendels 1993 (150 - 200 mg)	23	56	2	24	3.78 [0.96, 14.97]	1.48
Nemeroff 2007	40	60	8	94	5.10 [2.51, 10.34]	3.68
Rudolph 1998 (75 mg)	29	60	4	27	2.53 [0.96, 6.61]	2.53
Rudolph 1998 (225 mg)	34	55	4	27	2.96 [1.14, 7.67]	2.57
Rudolph 1998 (375 mg)	51	37	5	25	3.48 [1.53, 7.89]	3.11
Rudolph 1999	36	64	14	84	2.52 [1.45, 4.37]	4.66
Schatzberg 2006	46	56	13	83	3.33 [1.92, 5.77]	4.67
Schweizer 1994	20	53	11	67	1.94 [1.00, 3.77]	3.94
Sheehan 2009	36	59	15	80	2.40 [1.41, 4.08]	4.79
Silverstone 1999	52	76	33	86	1.46 [1.02, 2.10]	6.09
Thase 1997	34	61	18	84	2.03 [1.23, 3.34]	5.03
**Overall**					2.72 [2.26, 3.28]	

Heterogeneity: $\tau^2 = 0.11$, $I^2 = 46.43\%$, $H^2 = 1.87$

Test of $\theta_i = \theta_j$: Q(31) = 40.06, p = 0.13

Test of $\theta = 0$: z = 10.54, p = 0.00

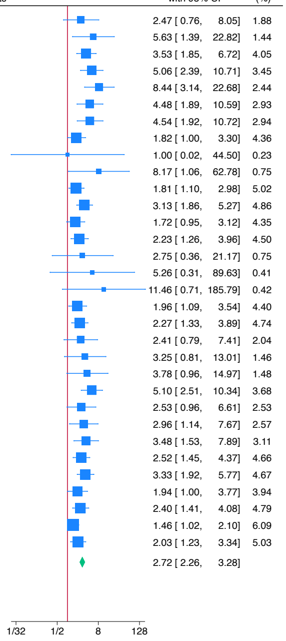

Random-effects Sidik–Jonkman model

Supplementary Figure 19: Meta-analysis of venlafaxine versus placebo on dry mouth

Graph 17/03/2024, 17.53

Study	Venlafaxine Events	Venlafaxine No events	Placebo Events	Placebo No events	Risk ratio with 95% CI	Weight (%)
600-B-367-EU (75 mg)	5	78	0	41	5.50 [0.31, 97.13]	0.63
600-B-367-EU (150 mg)	8	74	0	42	8.81 [0.52, 148.99]	0.65
Alvarez 2012	19	94	7	98	2.52 [1.11, 5.75]	4.46
Claghorn 1990	19	60	12	68	1.60 [0.84, 3.08]	5.62
Cunningham 1994	21	51	5	71	4.43 [1.77, 11.13]	3.93
Cunningham 1997 (IR)	21	75	4	46	2.73 [0.99, 7.53]	3.49
Cunningham 1997 (XR)	16	81	4	46	2.06 [0.73, 5.84]	3.37
EudraCT 2004-000562-13	17	110	1	119	16.06 [2.17, 118.84]	1.21
EudraCT 2007-007025-51	1	6	0	7	3.00 [0.14, 63.15]	0.56
Guelfi 1995	3	43	3	44	1.02 [0.22, 4.80]	1.87
Hewett 2009	13	174	11	186	1.25 [0.57, 2.71]	4.75
Hewett 2010	35	163	13	174	2.54 [1.39, 4.65]	5.99
Higuchi 2016 (fixed dose)	5	169	1	90	2.61 [0.31, 22.05]	1.08
Higuchi 2016 (flexible dose)	7	173	0	92	7.71 [0.45, 133.48]	0.64
Learned 2012	12	121	5	121	2.27 [0.82, 6.27]	3.48
Lieberman 2008 (225 mg)	30	87	9	116	3.56 [1.77, 7.18]	5.27
Mendels 1993 (25 mg)	14	65	4	22	1.15 [0.42, 3.19]	3.46
Mendels 1993 (50 - 75 mg)	16	60	3	23	1.82 [0.58, 5.76]	2.94
Mendels 1993 (150 - 200 mg)	23	56	3	23	2.52 [0.82, 7.72]	3.06
Nemeroff 2007	24	76	15	87	1.63 [0.91, 2.92]	6.15
Rudolph 1998 (75 mg)	16	73	4	27	1.39 [0.50, 3.85]	3.47
Rudolph 1998 (225 mg)	19	70	4	27	1.65 [0.61, 4.49]	3.56
Rudolph 1998 (375 mg)	17	71	4	26	1.45 [0.53, 3.97]	3.52
Schatzberg 2006	23	79	14	82	1.55 [0.85, 2.83]	6.00
Schweizer 1994	14	59	7	71	2.14 [0.91, 5.00]	4.32
Sheehan 2009	36	59	9	86	4.00 [2.04, 7.84]	5.47
Silverstone 1999	30	98	14	105	1.99 [1.11, 3.57]	6.15
Thase 1997	17	78	9	93	2.03 [0.95, 4.33]	4.88
**Overall**					2.16 [1.71, 2.74]	

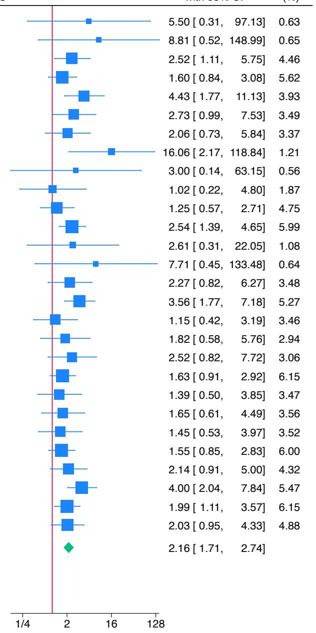

Heterogeneity: $\tau^2 = 0.15$, $I^2 = 40.74\%$, $H^2 = 1.69$

Test of $\theta_i = \theta_j$: $Q(27) = 23.19$, $p = 0.67$

Test of $\theta = 0$: $z = 6.43$, $p = 0.00$

Random-effects Sidik–Jonkman model

Supplementary Figure 20: Meta-analysis of venlafaxine versus placebo on dizziness

Graph 17/03/2024, 18.34

Study	Venlafaxine Events	Venlafaxine No events	Placebo Events	Placebo No events	Risk ratio with 95% CI	Weight (%)
600-B-367-EU (75 mg)	15	68	2	39	3.70 [0.89, 15.44]	2.65
600-B-367-EU (150 mg)	8	74	2	40	2.05 [0.46, 9.22]	2.45
Alvarez 2012	14	99	8	97	1.63 [0.71, 3.72]	5.25
Claghorn 1990	15	64	5	75	3.04 [1.16, 7.96]	4.46
Cunningham 1994	12	60	4	72	3.17 [1.07, 9.37]	3.87
Cunningham 1997 (IR)	34	62	3	47	5.90 [1.91, 18.27]	3.67
Cunningham 1997 (XR)	28	69	3	47	4.81 [1.54, 15.05]	3.63
EudraCT 2004-000562-13	6	121	7	113	0.81 [0.28, 2.34]	3.97
Guelfi 1995	4	42	1	46	4.09 [0.47, 35.20]	1.36
Hewett 2009	9	178	14	183	0.68 [0.30, 1.53]	5.34
Hewett 2010	27	171	11	176	2.32 [1.18, 4.54]	6.31
Higuchi 2016 (fixed dose)	10	164	2	90	2.64 [0.59, 11.81]	2.47
Higuchi 2016 (flexible dose)	18	162	3	88	3.03 [0.92, 10.03]	3.41
Khan 1998 (75 mg)	5	91	1	32	1.72 [0.21, 14.18]	1.41
Khan 1998 (150 mg)	2	94	0	33	1.75 [0.09, 35.60]	0.74
Khan 1998 (200 mg)	6	88	0	32	4.52 [0.26, 77.99]	0.82
Learned 2012	15	118	11	115	1.29 [0.62, 2.70]	5.83
Mendels 1993 (25 mg)	7	72	3	23	0.77 [0.21, 2.76]	3.11
Mendels 1993 (50 - 75 mg)	17	59	2	24	2.91 [0.72, 11.74]	2.74
Mendels 1993 (150 - 200 mg)	12	67	2	24	1.97 [0.47, 8.25]	2.64
Nemeroff 2007	13	87	3	99	4.42 [1.30, 15.04]	3.30
Rudolph 1998 (75 mg)	17	72	1	30	5.92 [0.82, 42.67]	1.58
Rudolph 1998 (225 mg)	20	69	1	30	6.97 [0.98, 49.77]	1.59
Rudolph 1998 (375 mg)	21	67	2	28	3.58 [0.89, 14.37]	2.76
Rudolph 1999	26	74	7	91	3.64 [1.66, 7.99]	5.51
Schatzberg 2006	17	85	5	91	3.20 [1.23, 8.34]	4.49
Schweizer 1994	12	61	3	75	4.27 [1.26, 14.54]	3.30
Sheehan 2009	15	80	3	92	5.00 [1.50, 16.71]	3.37
Silverstone 1999	49	79	20	99	2.28 [1.44, 3.59]	8.01
**Overall**					2.49 [1.90, 3.26]	

Heterogeneity: $\tau^2 = 0.18$, $I^2 = 37.85\%$, $H^2 = 1.61$

Test of $\theta_i = \theta_j$: Q(28) = 32.48, p = 0.26

Test of $\theta = 0$: z = 6.64, p = 0.00

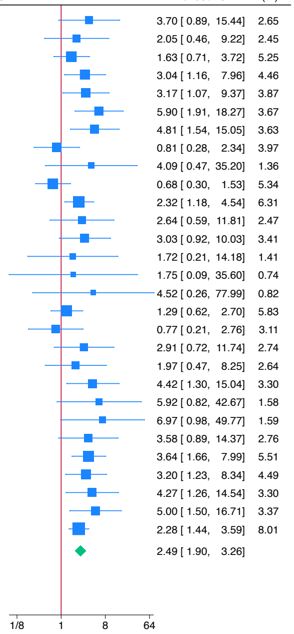

Random-effects Sidik–Jonkman model

Supplementary Figure 21: Meta-analysis of venlafaxine versus placebo on sweating

Study	Venlafaxine Events	Venlafaxine No events	Placebo Events	Placebo No events	Risk ratio with 95% CI	Weight (%)
600-B-367-EU (75 mg)	8	75	1	40	3.95 [0.51, 30.54]	2.30
600-B-367-EU (150 mg)	12	70	0	42	12.95 [0.79, 213.54]	1.29
Alvarez 2012	17	96	2	103	7.90 [1.87, 33.37]	4.18
Claghorn 1990	8	71	1	79	8.10 [1.04, 63.28]	2.28
Cunningham 1994	9	63	1	75	9.50 [1.23, 73.11]	2.31
Cunningham 1997 (IR)	13	83	1	49	6.77 [0.91, 50.28]	2.38
Cunningham 1997 (XR)	18	79	2	48	4.64 [1.12, 19.20]	4.28
EudraCT 2004-000562-13	12	115	4	116	2.83 [0.94, 8.55]	6.29
Guelfi 1995	7	39	1	46	7.15 [0.92, 55.86]	2.28
Hewett 2009	15	172	7	190	2.26 [0.94, 5.41]	8.56
Hewett 2010	16	182	7	180	2.16 [0.91, 5.13]	8.67
Higuchi 2016 (fixed dose)	3	171	1	91	1.59 [0.17, 15.03]	1.94
Higuchi 2016 (flexible dose)	15	165	1	90	7.58 [1.02, 56.51]	2.37
Learned 2012	15	118	3	123	4.74 [1.40, 15.97]	5.46
Lieberman 2008 (225 mg)	21	96	6	119	3.74 [1.56, 8.94]	8.59
Mendels 1993 (25 mg)	2	77	0	26	1.69 [0.08, 34.06]	1.13
Mendels 1993 (50 - 75 mg)	4	72	0	26	3.16 [0.18, 56.71]	1.22
Mendels 1993 (150 - 200 mg)	17	62	0	26	11.81 [0.73, 189.85]	1.31
Nemeroff 2007	14	86	2	100	7.14 [1.67, 30.61]	4.12
Rudolph 1999	10	90	3	95	3.27 [0.93, 11.51]	5.17
Schatzberg 2006	11	91	1	95	10.35 [1.36, 78.67]	2.33
Schweizer 1994	7	66	0	78	16.01 [0.93, 275.48]	1.25
Silverstone 1999	36	92	12	107	2.79 [1.52, 5.10]	12.56
Thase 1997	13	82	4	98	3.49 [1.18, 10.33]	6.44
Schweizer 1991	11	33	0	16	8.69 [0.54, 139.48]	1.31
**Overall**					3.99 [2.88, 5.54]	

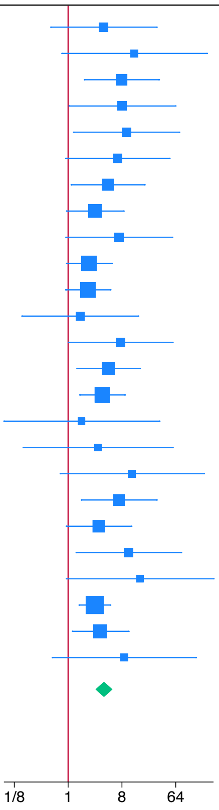

Heterogeneity: $\tau^2 = 0.13$, $I^2 = 20.46\%$, $H^2 = 1.26$

Test of $\theta_i = \theta_j$: Q(24) = 13.18, p = 0.96

Test of $\theta = 0$: z = 8.28, p = 0.00

Random-effects Sidik–Jonkman model

Supplementary Figure 22: Meta-analysis of venlafaxine versus placebo on somnolence

Study	Venlafaxine Events	Venlafaxine No events	Placebo Events	Placebo No events	Risk ratio with 95% CI	Weight (%)
600-B-367-EU (75 mg)	6	77	1	40	2.96 [0.37, 23.81]	1.07
600-B-367-EU (150 mg)	7	75	1	41	3.59 [0.46, 28.19]	1.10
Claghorn 1990	28	51	11	69	2.58 [1.38, 4.81]	8.17
Cunningham 1994	31	41	9	67	3.64 [1.86, 7.09]	7.46
Cunningham 1997 (IR)	23	73	4	46	2.99 [1.10, 8.18]	4.01
Cunningham 1997 (XR)	20	77	5	45	2.06 [0.82, 5.17]	4.64
EudraCT 2004-000562-13	12	115	3	117	3.78 [1.09, 13.06]	2.80
Guelfi 1995	1	45	0	47	3.06 [0.13, 73.33]	0.47
Hewett 2010	9	189	9	178	0.94 [0.38, 2.33]	4.78
Higuchi 2016 (fixed dose)	21	153	7	85	1.59 [0.70, 3.59]	5.58
Higuchi 2016 (flexible dose)	31	149	8	83	1.96 [0.94, 4.09]	6.52
Khan 1998 (75 mg)	7	89	0	33	5.26 [0.31, 89.63]	0.59
Khan 1998 (150 mg)	4	92	0	33	3.15 [0.17, 57.08]	0.57
Khan 1998 (200 mg)	4	90	0	32	3.13 [0.17, 56.52]	0.57
Learned 2012	7	126	3	123	2.21 [0.58, 8.36]	2.47
Lieberman 2008 (225 mg)	22	95	13	112	1.81 [0.96, 3.42]	7.95
Mendels 1993 (25 mg)	15	64	3	23	1.65 [0.52, 5.24]	3.16
Mendels 1993 (50 - 75 mg)	13	63	3	23	1.48 [0.46, 4.79]	3.08
Mendels 1993 (150 - 200 mg)	22	57	3	23	2.41 [0.79, 7.41]	3.33
Rudolph 1998 (75 mg)	15	74	1	30	5.22 [0.72, 37.94]	1.18
Rudolph 1998 (225 mg)	16	73	1	30	5.57 [0.77, 40.30]	1.19
Rudolph 1998 (375 mg)	23	65	2	28	3.92 [0.98, 15.65]	2.30
Rudolph 1999	8	92	6	92	1.31 [0.47, 3.63]	3.91
Schatzberg 2006	8	94	3	93	2.51 [0.69, 9.18]	2.58
Schweizer 1994	19	54	11	67	1.85 [0.94, 3.61]	7.43
Silverstone 1999	17	111	7	112	2.26 [0.97, 5.25]	5.31
Thase 1997	26	69	11	91	2.54 [1.33, 4.85]	7.79
**Overall**					2.23 [1.78, 2.78]	

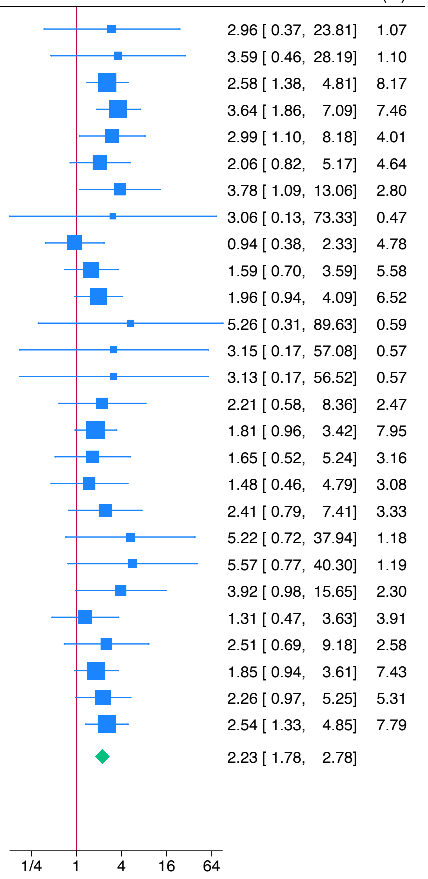

Heterogeneity: $\tau^2 = 0.05$, $I^2 = 16.92\%$, $H^2 = 1.20$

Test of $\theta_i = \theta_j$: $Q(26) = 13.23$, $p = 0.98$

Test of $\theta = 0$: $z = 7.09$, $p = 0.00$

Random-effects Sidik–Jonkman model

Supplementary Figure 23: Meta-analysis of venlafaxine versus placebo on constipation

Study	Venlafaxine Events	Venlafaxine No events	Placebo Events	Placebo No events	Risk ratio with 95% CI	Weight (%)
600-B-367-EU (75 mg)	1	82	1	40	0.49 [0.03, 7.70]	1.13
600-B-367-EU (150 mg)	5	77	1	41	2.56 [0.31, 21.22]	1.79
Alvarez 2012	11	102	1	104	10.22 [1.34, 77.81]	1.91
Claghorn 1990	11	68	1	79	11.14 [1.47, 84.25]	1.92
Cunningham 1994	16	56	3	73	5.63 [1.71, 18.51]	4.22
Cunningham 1997 (IR)	14	82	2	48	3.65 [0.86, 15.41]	3.26
Cunningham 1997 (XR)	16	81	2	48	4.12 [0.99, 17.23]	3.30
EudraCT 2004-000562-13	6	121	5	115	1.13 [0.36, 3.62]	4.36
Guelfi 1995	2	44	1	46	2.04 [0.19, 21.77]	1.47
Hewett 2010	12	186	3	184	3.78 [1.08, 13.18]	3.97
Higuchi 2016 (fixed dose)	17	157	4	88	2.25 [0.78, 6.48]	4.86
Higuchi 2016 (flexible dose)	17	163	4	87	2.15 [0.74, 6.20]	4.86
Learned 2012	12	121	9	117	1.26 [0.55, 2.89]	6.26
Lieberman 2008 (225 mg)	9	108	2	123	4.81 [1.06, 21.79]	3.04
Mendels 1993 (25 mg)	6	73	2	24	0.99 [0.21, 4.60]	2.97
Mendels 1993 (50 - 75 mg)	9	67	2	24	1.54 [0.36, 6.67]	3.18
Mendels 1993 (150 - 200 mg)	15	64	2	24	2.47 [0.60, 10.08]	3.37
Nemeroff 2007	10	90	5	97	2.04 [0.72, 5.76]	4.98
Rudolph 1998 (75 mg)	17	72	3	28	1.97 [0.62, 6.28]	4.37
Rudolph 1998 (225 mg)	10	79	3	28	1.16 [0.34, 3.95]	4.08
Rudolph 1998 (375 mg)	17	71	4	26	1.45 [0.53, 3.97]	5.15
Schatzberg 2006	22	80	4	92	5.18 [1.85, 14.47]	5.03
Schweizer 1994	9	64	6	72	1.60 [0.60, 4.28]	5.29
Sheehan 2009	24	71	11	84	2.18 [1.13, 4.20]	7.55
Silverstone 1999	22	106	13	106	1.57 [0.83, 2.98]	7.68
**Overall**					2.24 [1.64, 3.04]	

Heterogeneity: $\tau^2 = 0.21$, $I^2 = 38.26\%$, $H^2 = 1.62$

Test of $\theta_i = \theta_j$: Q(24) = 21.26, p = 0.62

Test of $\theta = 0$: z = 5.13, p = 0.00

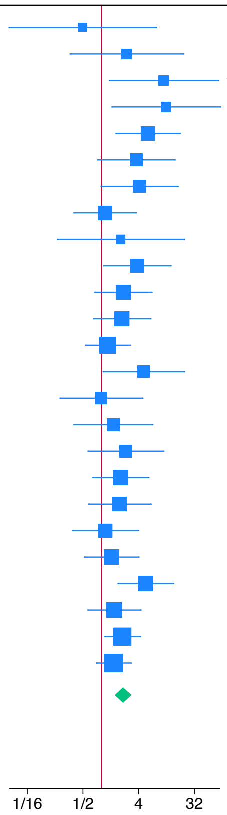

Random-effects Sidik–Jonkman model

Supplementary Figure 24: Meta-analysis of venlafaxine versus placebo on nervousness

Study	Venlafaxine Events	Venlafaxine No events	Placebo Events	Placebo No events	Risk ratio with 95% CI	Weight (%)
600-B-367-EU (75 mg)	1	82	1	40	0.49 [0.03, 7.70]	2.23
600-B-367-EU (150 mg)	2	80	1	41	1.02 [0.10, 10.97]	2.89
Claghorn 1990	7	72	6	74	1.18 [0.42, 3.36]	9.48
Cunningham 1994	15	57	9	67	1.76 [0.82, 3.77]	12.78
Guelfi 1995	3	43	0	47	7.15 [0.38, 134.66]	1.98
Mendels 1993 (25 mg)	4	75	2	24	0.66 [0.13, 3.39]	5.26
Mendels 1993 (50 - 75 mg)	7	69	1	25	2.39 [0.31, 18.55]	3.70
Mendels 1993 (150 - 200 mg)	13	66	1	25	4.28 [0.59, 31.15]	3.90
Rudolph 1998 (75 mg)	19	70	1	30	6.62 [0.92, 47.40]	3.95
Rudolph 1998 (225 mg)	12	77	1	30	4.18 [0.57, 30.84]	3.85
Rudolph 1998 (375 mg)	11	77	2	28	1.88 [0.44, 7.98]	6.30
Rudolph 1999	12	88	5	93	2.35 [0.86, 6.43]	9.89
Schatzberg 2006	2	100	2	94	0.94 [0.14, 6.55]	4.04
Schweizer 1994	10	63	3	75	3.56 [1.02, 12.43]	7.67
Silverstone 1999	23	105	8	111	2.67 [1.24, 5.74]	12.73
Thase 1997	16	79	4	98	4.29 [1.49, 12.39]	9.34
**Overall**					2.20 [1.43, 3.40]	

Heterogeneity: $\tau^2 = 0.23$, $I^2 = 33.36\%$, $H^2 = 1.50$

Test of $\theta_i = \theta_j$: Q(15) = 11.11, p = 0.74

Test of $\theta = 0$: z = 3.56, p = 0.00

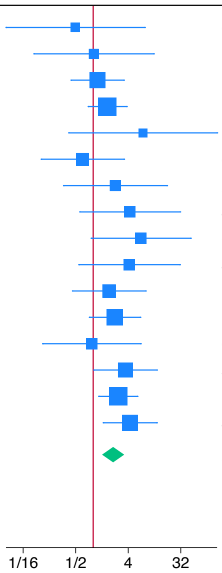

Random-effects Sidik–Jonkman model

Supplementary Figure 25: Meta-analysis of venlafaxine versus placebo on insomnia

Study	Venlafaxine Events	Venlafaxine No events	Placebo Events	Placebo No events	Risk ratio with 95% CI	Weight (%)
600-B-367-EU (75 mg)	2	81	2	39	0.49 [0.07, 3.38]	1.36
600-B-367-EU (150 mg)	3	79	1	41	1.54 [0.16, 14.32]	1.04
Alvarez 2012	14	99	5	100	2.60 [0.97, 6.97]	4.15
Claghorn 1990	13	66	6	74	2.19 [0.88, 5.48]	4.60
Cunningham 1994	7	65	3	73	2.46 [0.66, 9.16]	2.65
EudraCT 2004-000562-13	14	113	8	112	1.65 [0.72, 3.80]	5.24
Guelfi 1995	3	43	3	44	1.02 [0.22, 4.80]	2.01
Hewett 2009	7	180	4	193	1.84 [0.55, 6.20]	3.02
Hewett 2010	13	185	8	179	1.53 [0.65, 3.62]	5.04
Higuchi 2016 (fixed dose)	4	170	3	89	0.70 [0.16, 3.08]	2.18
Higuchi 2016 (flexible dose)	8	172	3	88	1.35 [0.37, 4.96]	2.69
Khan 1998 (75 mg)	5	91	0	33	3.86 [0.22, 67.91]	0.65
Khan 1998 (150 mg)	3	93	0	33	2.45 [0.13, 46.29]	0.62
Khan 1998 (200 mg)	5	89	0	32	3.82 [0.22, 67.25]	0.65
Learned 2012	7	126	11	115	0.60 [0.24, 1.51]	4.61
Mendels 1993 (25 mg)	14	65	4	22	1.15 [0.42, 3.19]	3.95
Mendels 1993 (50 - 75 mg)	10	66	4	22	0.86 [0.29, 2.49]	3.67
Mendels 1993 (150 - 200 mg)	19	60	3	23	2.08 [0.67, 6.48]	3.36
Nemeroff 2007	22	78	14	88	1.60 [0.87, 2.95]	7.51
Rudolph 1998 (75 mg)	20	69	3	28	2.32 [0.74, 7.28]	3.32
Rudolph 1998 (225 mg)	18	71	3	28	2.09 [0.66, 6.61]	3.28
Rudolph 1998 (375 mg)	12	76	3	27	1.36 [0.41, 4.51]	3.09
Schatzberg 2006	10	92	4	92	2.35 [0.76, 7.25]	3.40
Schweizer 1994	15	58	10	68	1.60 [0.77, 3.34]	6.13
Sheehan 2009	18	77	8	87	2.25 [1.03, 4.92]	5.67
Silverstone 1999	41	87	12	107	3.18 [1.76, 5.75]	7.73
Thase 1997	33	62	15	87	2.36 [1.37, 4.06]	8.40
**Overall**					1.73 [1.37, 2.19]	

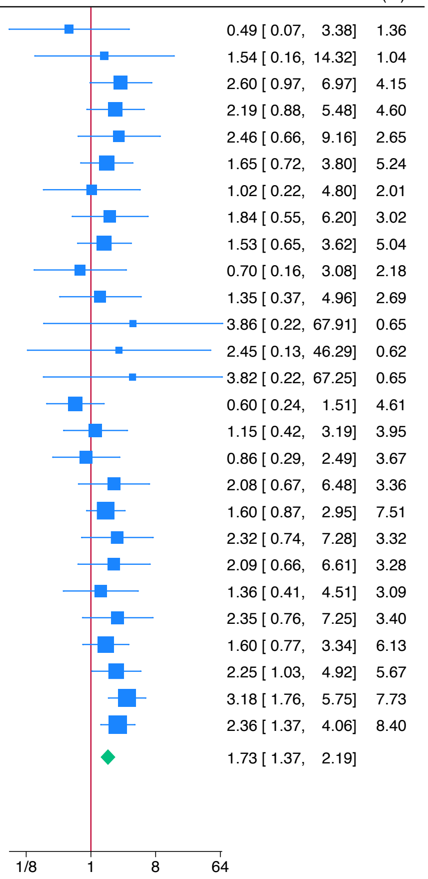

Heterogeneity: $\tau^2 = 0.10$, $I^2 = 26.87\%$, $H^2 = 1.37$

Test of $\theta_i = \theta_j$: Q(26) = 19.55, p = 0.81

Test of $\theta = 0$: z = 4.57, p = 0.00

Random-effects Sidik–Jonkman model

Supplementary Figure 26: Meta-analysis of venlafaxine versus placebo on asthenia

Study	Venlafaxine Events	Venlafaxine No events	Placebo Events	Placebo No events	Risk ratio with 95% CI	Weight (%)
600-B-367-EU (75 mg)	5	78	1	40	2.47 [0.30, 20.46]	2.03
600-B-367-EU (150 mg)	3	79	1	41	1.54 [0.16, 14.32]	1.84
Alvarez 2012	11	102	6	99	1.70 [0.65, 4.44]	7.57
Claghorn 1990	5	74	2	78	2.53 [0.51, 12.67]	3.31
Cunningham 1994	7	65	3	73	2.46 [0.66, 9.16]	4.67
EudraCT 2004-000562-13	10	117	2	118	4.72 [1.06, 21.12]	3.75
Guelfi 1995	2	44	0	47	5.11 [0.25, 103.55]	1.04
Hewett 2009	9	178	4	193	2.37 [0.74, 7.57]	5.69
Hewett 2010	16	182	13	174	1.16 [0.57, 2.35]	11.20
Khan 1998 (75 mg)	5	91	0	33	3.86 [0.22, 67.91]	1.14
Khan 1998 (150 mg)	5	91	0	33	3.86 [0.22, 67.91]	1.14
Khan 1998 (200 mg)	3	91	0	32	2.43 [0.13, 45.84]	1.09
Learned 2012	13	120	5	121	2.46 [0.90, 6.71]	7.10
Lieberman 2008 (225 mg)	7	110	8	117	0.93 [0.35, 2.50]	7.30
Mendels 1993 (25 mg)	10	69	3	23	1.10 [0.33, 3.69]	5.32
Mendels 1993 (50 - 75 mg)	8	68	3	23	0.91 [0.26, 3.18]	5.06
Mendels 1993 (150 - 200 mg)	13	66	2	24	2.14 [0.52, 8.86]	4.10
Nemeroff 2007	10	90	5	97	2.04 [0.72, 5.76]	6.75
Rudolph 1999	10	90	2	96	4.90 [1.10, 21.79]	3.78
Schatzberg 2006	12	90	5	91	2.26 [0.83, 6.17]	7.07
Schweizer 1994	9	64	10	68	0.96 [0.41, 2.23]	9.02
**Overall**					1.78 [1.30, 2.43]	

Heterogeneity: $\tau^2 = 0.10$, $I^2 = 19.72\%$, $H^2 = 1.25$

Test of $\theta_i = \theta_j$: Q(20) = 12.71, p = 0.89

Test of $\theta = 0$: z = 3.58, p = 0.00

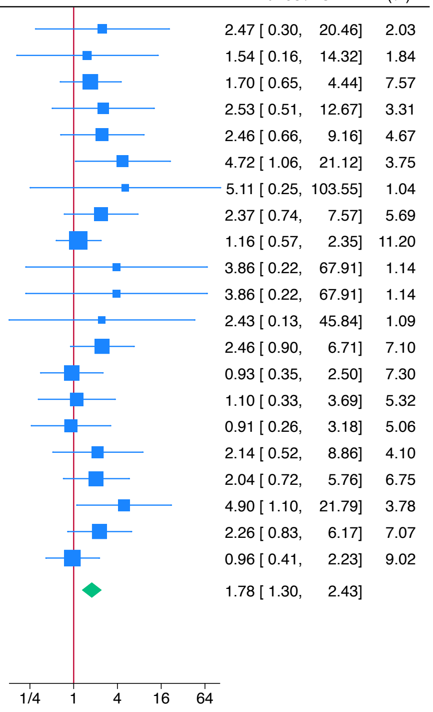

Random-effects Sidik–Jonkman model

Supplementary Figure 27: Meta-analysis of venlafaxine versus placebo on tremor

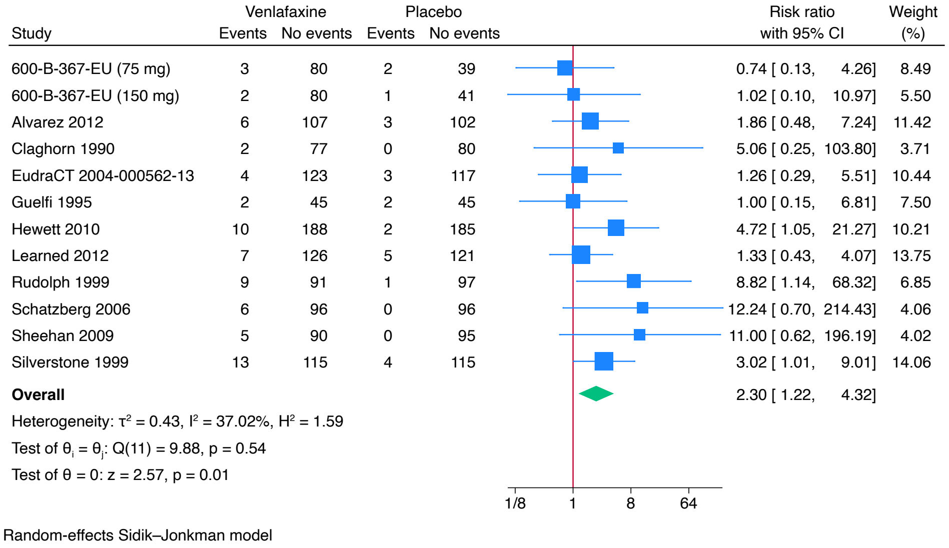

Supplementary Figure 28: Meta-analysis of venlafaxine versus placebo on decreased appetite

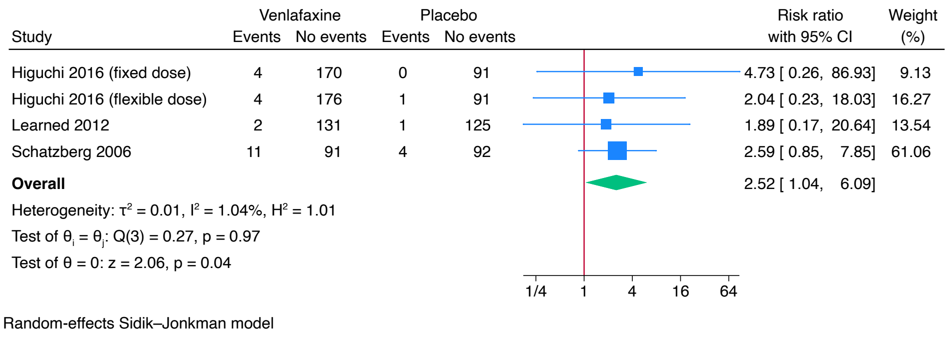

Supplementary Figure 29: Meta-analysis of venlafaxine versus placebo on abdominal pain

Study	Venlafaxine Events	Venlafaxine No events	Placebo Events	Placebo No events	Risk ratio with 95% CI	Weight (%)
600-B-367-EU (75 mg)	0	83	2	39	0.10 [0.00, 2.04]	8.14
600-B-367-EU (150 mg)	2	80	1	41	1.02 [0.10, 10.97]	11.43
EudraCT 2004-000562-13	8	119	11	109	0.69 [0.29, 1.65]	26.35
Hewett 2010	9	189	8	179	1.06 [0.42, 2.70]	25.66
Higuchi 2016 (fixed dose)	5	169	3	88	0.87 [0.21, 3.57]	19.85
Higuchi 2016 (flexible dose)	0	180	4	88	0.06 [0.00, 1.05]	8.58
**Overall**					0.58 [0.22, 1.57]	

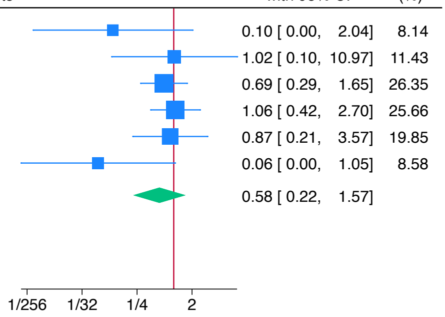

Heterogeneity: $\tau^2 = 0.77$, $I^2 = 58.37\%$, $H^2 = 2.40$

Test of $\theta_i = \theta_j$: Q(5) = 5.40, p = 0.37

Test of $\theta = 0$: z = -1.07, p = 0.28

Random-effects Sidik–Jonkman model

Supplementary Figure 30: Meta-analysis of venlafaxine versus placebo on abnormal dreams

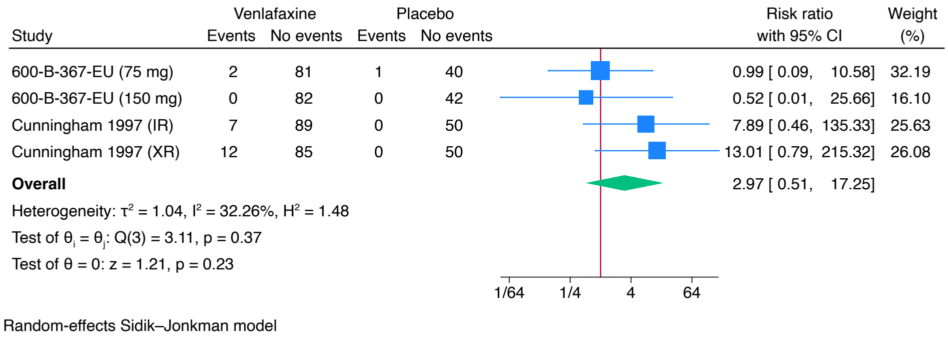

Supplementary Figure 31: Meta-analysis of venlafaxine versus placebo on abnormal vision

Study	Venlafaxine Events	Venlafaxine No events	Placebo Events	Placebo No events	Risk ratio with 95% CI	Weight (%)
600-B-367-EU (75 mg)	0	83	1	40	0.17 [0.01, 4.00]	5.77
600-B-367-EU (150 mg)	0	82	0	42	0.52 [0.01, 25.66]	4.03
Alvarez 2012	6	107	2	103	2.79 [0.58, 13.51]	16.06
EudraCT 2004-000562-13	3	124	0	120	6.62 [0.35, 126.78]	6.54
Guelfi 1995	1	45	0	47	3.06 [0.13, 73.33]	5.78
Lieberman 2008 (225 mg)	5	112	3	122	1.78 [0.44, 7.29]	18.21
Schatzberg 2006	8	94	5	91	1.51 [0.51, 4.44]	23.23
Sheehan 2009	10	85	3	92	3.33 [0.95, 11.73]	20.38
**Overall**					1.95 [0.85, 4.47]	

Heterogeneity: $\tau^2 = 0.46$, $I^2 = 35.92\%$, $H^2 = 1.56$

Test of $\theta_i = \theta_j$: $Q(7) = 4.60$, $p = 0.71$

Test of $\theta = 0$: $z = 1.58$, $p = 0.11$

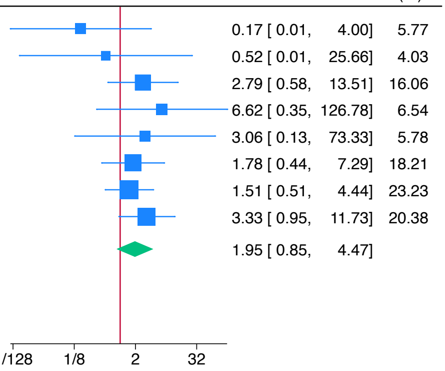

Random-effects Sidik–Jonkman model

Supplementary Figure 32: Meta-analysis of venlafaxine versus placebo on agitation

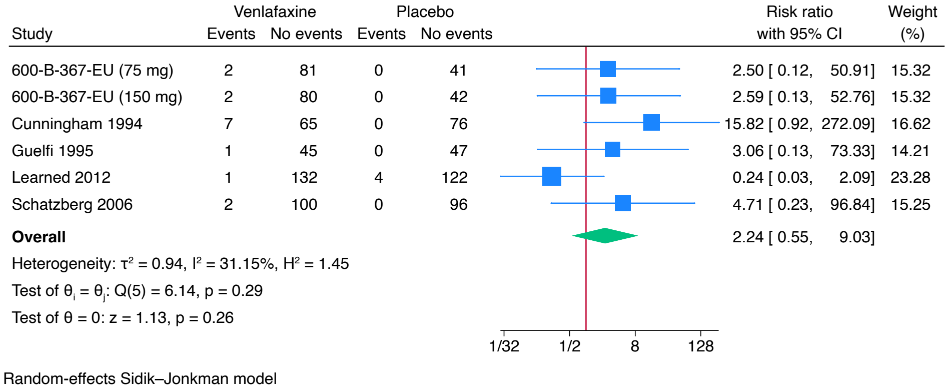

Supplementary Figure 33: Meta-analysis of venlafaxine versus placebo on back pain

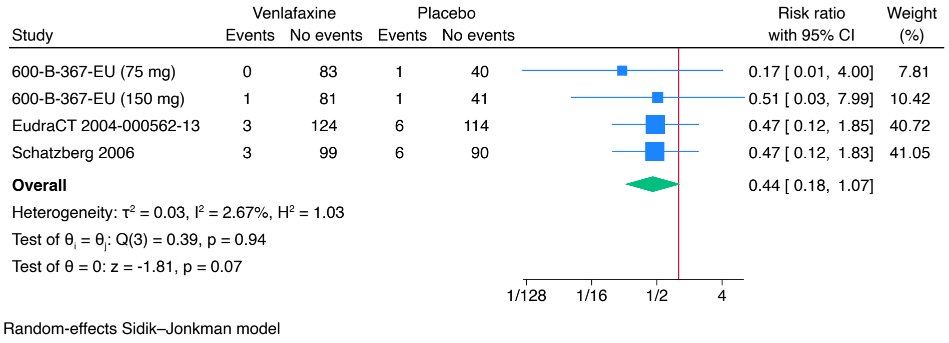

STATA®

Supplementary Figure 34: Meta-analysis of venlafaxine versus placebo on increased blood pressure

Study	Venlafaxine Events	Venlafaxine No events	Placebo Events	Placebo No events	Risk ratio with 95% CI	Weight (%)
Higuchi 2016 (fixed dose)	4	170	1	91	2.11 [0.24, 18.65]	20.08
Higuchi 2016 (flexible dose)	5	175	1	90	2.53 [0.30, 21.32]	20.89
Schatzberg 2006	5	97	5	91	0.94 [0.28, 3.15]	59.03
**Overall**					1.36 [0.50, 3.70]	

Heterogeneity: $\tau^2 = 0.06$, $I^2 = 6.64\%$, $H^2 = 1.07$

Test of $\theta_i = \theta_j$: $Q(2) = 0.84$, $p = 0.66$

Test of $\theta = 0$: $z = 0.60$, $p = 0.55$

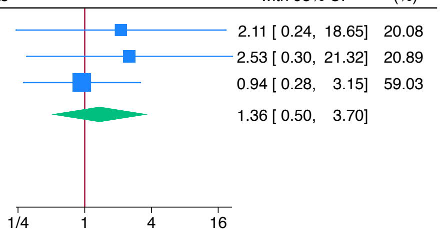

Random-effects Sidik–Jonkman model

Supplementary Figure 35: Meta-analysis of venlafaxine versus placebo on coughing

Study	Venlafaxine Events	Venlafaxine No events	Placebo Events	Placebo No events	Risk ratio with 95% CI	Weight (%)
600-B-367-EU (75 mg)	1	82	0	41	1.50 [0.06, 36.04]	17.33
600-B-367-EU (150 mg)	1	81	1	41	0.51 [0.03, 7.99]	23.07
Schatzberg 2006	2	100	4	92	0.47 [0.09, 2.51]	59.60
**Overall**					0.59 [0.15, 2.23]	

Heterogeneity: $\tau^2 = 0.05$, $I^2 = 3.02\%$, $H^2 = 1.03$

Test of $\theta_i = \theta_j$: $Q(2) = 0.41$, $p = 0.81$

Test of $\theta = 0$: $z = -0.78$, $p = 0.43$

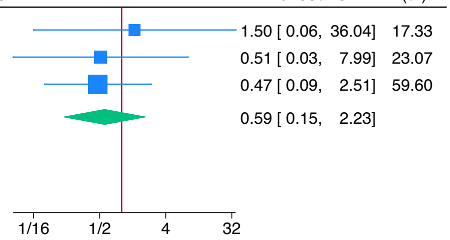

Random-effects Sidik–Jonkman model

Supplementary Figure 36: Meta-analysis of venlafaxine versus placebo on diarrhoea

Study	Venlafaxine Events	Venlafaxine No events	Placebo Events	Placebo No events	Risk ratio with 95% CI	Weight (%)
600-B-367-EU (75 mg)	3	80	3	38	0.49 [0.10, 2.34]	3.53
600-B-367-EU (150 mg)	1	81	3	39	0.17 [0.02, 1.59]	3.48
Alvarez 2012	5	108	5	100	0.93 [0.28, 3.12]	4.55
Cunningham 1994	7	65	5	71	1.48 [0.49, 4.45]	4.27
Cunningham 1997 (IR)	5	91	3	47	0.87 [0.22, 3.48]	3.46
Cunningham 1997 (XR)	13	84	3	47	2.23 [0.67, 7.48]	3.48
EudraCT 2004-000562-13	3	124	6	114	0.47 [0.12, 1.85]	5.42
EudraCT 2007-007025-51	0	7	2	5	0.20 [0.01, 3.54]	2.20
Guelfi 1995	0	46	1	46	0.34 [0.01, 8.15]	1.30
Hewett 2010	10	188	9	178	1.05 [0.44, 2.53]	8.13
Higuchi 2016 (fixed dose)	6	168	3	88	1.05 [0.27, 4.09]	3.46
Higuchi 2016 (flexible dose)	8	172	4	88	1.02 [0.32, 3.31]	4.65
Learned 2012	8	125	8	118	0.95 [0.37, 2.45]	7.21
Nemeroff 2007	9	91	9	93	1.02 [0.42, 2.46]	7.82
Rudolph 1999	14	86	9	89	1.52 [0.69, 3.36]	7.98
Schatzberg 2006	12	90	13	83	0.87 [0.42, 1.81]	11.76
Silverstone 1999	22	106	19	100	1.08 [0.61, 1.89]	17.29
**Overall**					1.00 [0.78, 1.28]	

Heterogeneity: $I^2 = 0.00\%$, $H^2 = 1.00$

Test of $\theta_i = \theta_j$: Q(16) = 9.58, p = 0.89

Test of $\theta = 0$: z = -0.02, p = 0.99

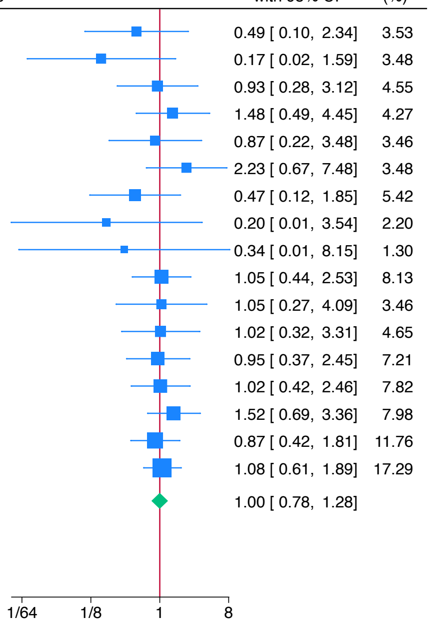

Fixed-effects Mantel–Haenszel model

Supplementary Figure 37: Meta-analysis of venlafaxine versus placebo on dyspepsia

Study	Venlafaxine Events	Venlafaxine No events	Placebo Events	Placebo No events	Risk ratio with 95% CI	Weight (%)
600-B-367-EU (75 mg)	2	81	2	39	0.49 [0.07, 3.38]	5.78
600-B-367-EU (150 mg)	1	81	2	40	0.26 [0.02, 2.74]	3.91
Cunningham 1994	2	70	5	71	0.42 [0.08, 2.11]	8.01
Learned 2012	2	131	2	124	0.95 [0.14, 6.62]	5.67
Nemeroff 2007	9	91	16	86	0.57 [0.27, 1.24]	25.56
Schatzberg 2006	11	91	8	88	1.29 [0.54, 3.08]	21.72
Silverstone 1999	13	115	16	103	0.76 [0.38, 1.50]	29.35
**Overall**					0.72 [0.44, 1.16]	

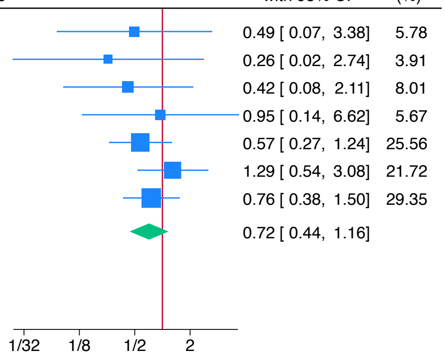

Heterogeneity: $\tau^2 = 0.08$, $I^2 = 20.08\%$, $H^2 = 1.25$

Test of $\theta_i = \theta_j$: $Q(6) = 3.48$, $p = 0.75$

Test of $\theta = 0$: $z = -1.36$, $p = 0.17$

Random-effects Sidik–Jonkman model

Supplementary Figure 38: Meta-analysis of venlafaxine versus placebo on flatulence

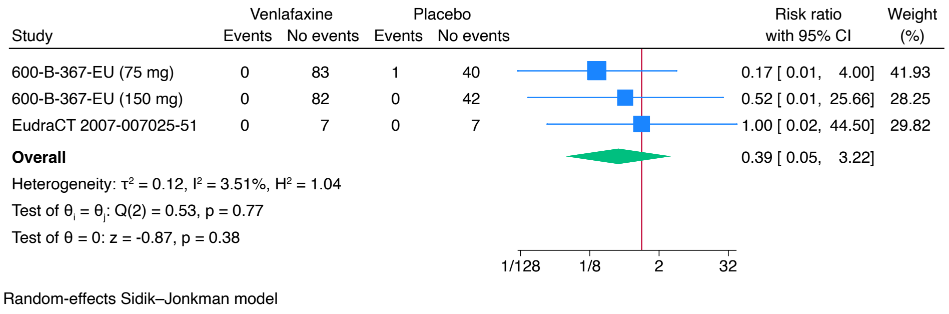

Supplementary Figure 39: Meta-analysis of venlafaxine versus placebo on headache

Study	Venlafaxine Events	Venlafaxine No events	Placebo Events	Placebo No events	Risk ratio with 95% CI	Weight (%)
600-B-367-EU (75 mg)	11	72	5	36	1.09 [0.40, 2.92]	1.76
600-B-367-EU (150 mg)	7	75	6	36	0.60 [0.21, 1.67]	1.65
Alvarez 2012	32	81	26	79	1.14 [0.73, 1.78]	6.73
Claghorn 1990	21	58	17	63	1.25 [0.72, 2.19]	4.75
Cunningham 1994	22	50	25	51	0.93 [0.58, 1.49]	6.12
EudraCT 2004-000562-13	29	98	34	86	0.81 [0.53, 1.24]	7.09
EudraCT 2007-007025-51	0	7	1	6	0.33 [0.02, 7.02]	0.20
Guelfi 1995	2	44	4	43	0.51 [0.10, 2.65]	0.66
Hewett 2009	25	162	19	178	1.39 [0.79, 2.43]	4.71
Hewett 2010	28	170	31	156	0.85 [0.53, 1.37]	6.19
Higuchi 2016 (fixed dose)	16	158	7	85	1.21 [0.52, 2.83]	2.31
Higuchi 2016 (flexible dose)	18	162	7	84	1.30 [0.56, 3.00]	2.39
Learned 2012	28	105	24	102	1.11 [0.68, 1.80]	5.86
Mendels 1993 (25 mg)	20	59	7	19	0.94 [0.45, 1.97]	2.98
Mendels 1993 (50 - 75 mg)	19	57	7	19	0.93 [0.44, 1.95]	2.94
Mendels 1993 (150 - 200 mg)	17	62	7	19	0.80 [0.37, 1.71]	2.83
Nemeroff 2007	36	64	34	68	1.08 [0.74, 1.58]	8.34
Rudolph 1998 (75 mg)	23	66	7	24	1.14 [0.55, 2.40]	2.96
Rudolph 1998 (225 mg)	23	66	7	24	1.14 [0.55, 2.40]	2.96
Rudolph 1998 (375 mg)	22	66	8	22	0.94 [0.47, 1.88]	3.31
Schatzberg 2006	27	75	21	75	1.21 [0.74, 1.99]	5.69
Schweizer 1994	19	54	21	57	0.97 [0.57, 1.65]	5.13
Silverstone 1999	57	71	59	60	0.90 [0.69, 1.17]	12.45
**Overall**					1.01 [0.88, 1.15]	

Heterogeneity: $\tau^2 = 0.02$, $I^2 = 19.96\%$, $H^2 = 1.25$

Test of $\theta_i = \theta_j$: Q(22) = 8.69, p = 0.99

Test of $\theta = 0$: z = 0.09, p = 0.93

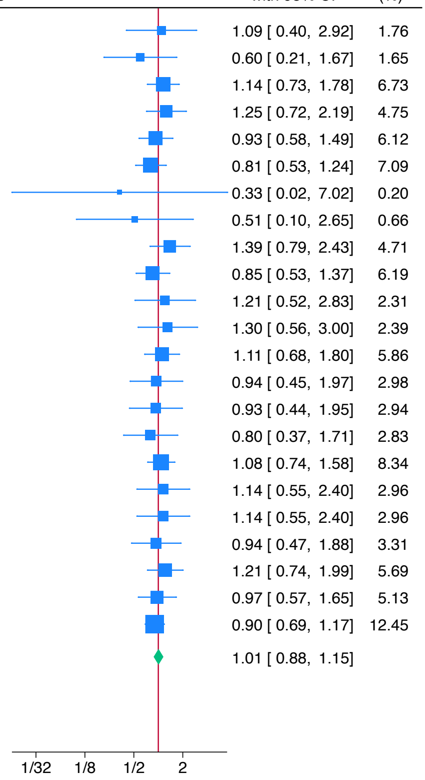

Random-effects Sidik–Jonkman model

Supplementary Figure 40: Meta-analysis of venlafaxine versus placebo on infection

Study	Venlafaxine Events	Venlafaxine No events	Placebo Events	Placebo No events	Risk ratio with 95% CI	Weight (%)
600-B-367-EU (75 mg)	3	80	2	39	0.74 [0.13, 4.26]	6.51
600-B-367-EU (150 mg)	0	82	1	41	0.17 [0.01, 4.15]	4.81
Cunningham 1994	8	64	7	69	1.21 [0.46, 3.16]	16.57
EudraCT 2004-000562-13	2	125	10	110	0.19 [0.04, 0.84]	25.02
Mendels 1993 (25 mg)	10	69	2	24	1.65 [0.39, 7.03]	7.32
Mendels 1993 (50 - 75 mg)	3	73	2	24	0.51 [0.09, 2.90]	7.25
Mendels 1993 (150 - 200 mg)	7	72	1	25	2.30 [0.30, 17.86]	3.66
Rudolph 1999	13	87	6	92	2.12 [0.84, 5.36]	14.74
Schweizer 1994	3	70	6	72	0.53 [0.14, 2.06]	14.11
**Overall**					0.93 [0.61, 1.43]	

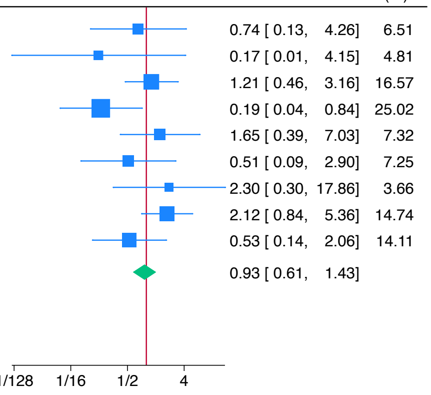

Heterogeneity: $I^2 = 28.97\%$, $H^2 = 1.41$

Test of $\theta_i = \theta_j$: Q(8) = 11.26, p = 0.19

Test of $\theta = 0$: z = -0.31, p = 0.75

Fixed-effects Mantel–Haenszel model

Supplementary Figure 41: Meta-analysis of venlafaxine versus placebo on influenza

Study	Venlafaxine Events	Venlafaxine No events	Placebo Events	Placebo No events	Risk ratio with 95% CI	Weight (%)
600-B-367-EU (75 mg)	2	81	2	39	0.49 [0.07, 3.38]	16.83
600-B-367-EU (150 mg)	0	82	1	41	0.17 [0.01, 4.15]	7.93
Guelfi 1995	1	45	1	46	1.02 [0.07, 15.85]	10.09
Hewett 2010	4	194	9	178	0.42 [0.13, 1.34]	28.62
Rudolph 1999	7	93	4	94	1.72 [0.52, 5.67]	27.92
Schatzberg 2006	2	100	0	96	4.71 [0.23, 96.84]	8.62
**Overall**					0.80 [0.30, 2.14]	

Heterogeneity: $\tau^2 = 0.52$, $I^2 = 37.51\%$, $H^2 = 1.60$

Test of $\theta_i = \theta_j$: $Q(5) = 5.23$, $p = 0.39$

Test of $\theta = 0$: $z = -0.44$, $p = 0.66$

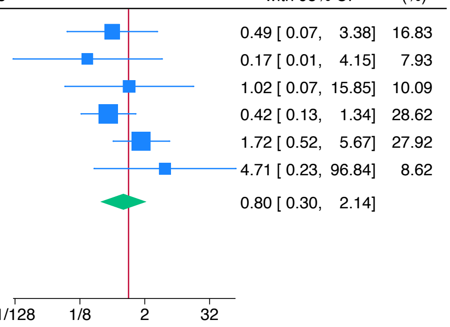

Random-effects Sidik–Jonkman model

Supplementary Figure 42: Meta-analysis of venlafaxine versus placebo on malaise

Study	Venlafaxine Events	Venlafaxine No events	Placebo Events	Placebo No events	Risk ratio with 95% CI	Weight (%)
600-B-367-EU (75 mg)	0	83	1	40	0.17 [0.01, 4.00]	13.08
600-B-367-EU (150 mg)	0	82	0	42	0.52 [0.01, 25.66]	9.25
Higuchi 2016 (fixed dose)	9	165	2	90	2.38 [0.52, 10.78]	35.52
Higuchi 2016 (flexible dose)	11	169	3	88	1.85 [0.53, 6.48]	42.15
**Overall**					1.31 [0.37, 4.69]	

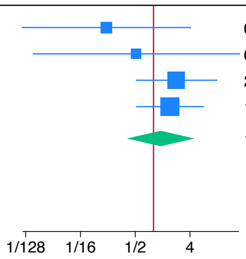

Heterogeneity: $\tau^2 = 0.59$, $I^2 = 36.17\%$, $H^2 = 1.57$

Test of $\theta_i = \theta_j$: $Q(3) = 2.58$, $p = 0.46$

Test of $\theta = 0$: $z = 0.42$, $p = 0.67$

Random-effects Sidik–Jonkman model

Supplementary Figure 43: Meta-analysis of venlafaxine versus placebo on nasopharyngitis

Study	Venlafaxine Events	Venlafaxine No events	Placebo Events	Placebo No events	Risk ratio with 95% CI	Weight (%)
Alvarez 2012	4	109	9	96	0.41 [0.13, 1.30]	9.04
Hewett 2010	11	187	9	178	1.15 [0.49, 2.72]	14.83
Higuchi 2016 (fixed dose)	35	139	20	72	0.93 [0.57, 1.51]	32.95
Higuchi 2016 (flexible dose)	32	148	21	70	0.77 [0.47, 1.26]	32.84
Learned 2012	6	127	7	119	0.81 [0.28, 2.35]	10.34
**Overall**					0.83 [0.57, 1.19]	

Heterogeneity: $\tau^2 = 0.04$, $I^2 = 25.40\%$, $H^2 = 1.34$

Test of $\theta_i = \theta_j$: $Q(4) = 2.27$, $p = 0.69$

Test of $\theta = 0$: $z = -1.02$, $p = 0.31$

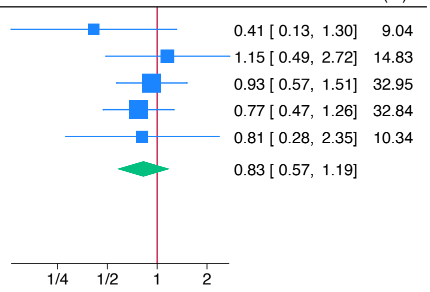

Random-effects Sidik–Jonkman model

Supplementary Figure 44: Meta-analysis of venlafaxine versus placebo on palpitations

Study	Venlafaxine Events	Venlafaxine No events	Placebo Events	Placebo No events	Risk ratio with 95% CI	Weight (%)
600-B-367-EU (75 mg)	0	83	1	40	0.17 [0.01, 4.00]	6.53
600-B-367-EU (150 mg)	3	79	1	41	1.54 [0.16, 14.32]	12.13
Higuchi 2016 (fixed dose)	8	166	2	89	2.09 [0.45, 9.65]	21.78
Higuchi 2016 (flexible dose)	6	174	3	89	1.02 [0.26, 3.99]	25.44
Learned 2012	5	128	2	124	2.37 [0.47, 11.99]	20.01
Rudolph 1998 (75 mg)	1	22	0	13	1.75 [0.08, 40.11]	6.71
Rudolph 1998 (225 mg)	2	20	0	13	3.04 [0.16, 58.90]	7.41
**Overall**					1.48 [0.63, 3.47]	

Heterogeneity: $\tau^2 = 0.26$, $I^2 = 19.73\%$, $H^2 = 1.25$

Test of $\theta_i = \theta_j$: $Q(6) = 2.85$, $p = 0.83$

Test of $\theta = 0$: $z = 0.91$, $p = 0.36$

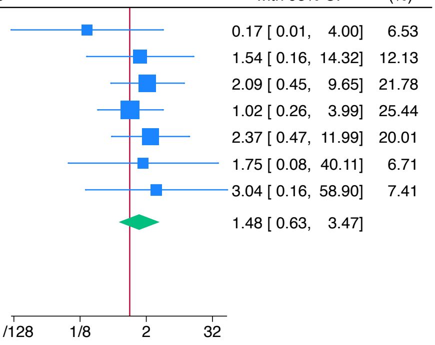

Random-effects Sidik–Jonkman model

Supplementary Figure 45: Meta-analysis of venlafaxine versus placebo on rhinitis

Study	Venlafaxine Events	Venlafaxine No events	Placebo Events	Placebo No events	Risk ratio with 95% CI	Weight (%)
600-B-367-EU (75 mg)	2	81	1	40	0.99 [0.09, 10.58]	11.97
600-B-367-EU (150 mg)	1	81	0	42	1.55 [0.06, 37.35]	5.89
Cunningham 1994	5	67	0	76	11.60 [0.65, 206.14]	4.35
Schweizer 1994	4	69	9	69	0.47 [0.15, 1.48]	77.79
**Overall**					1.08 [0.49, 2.40]	

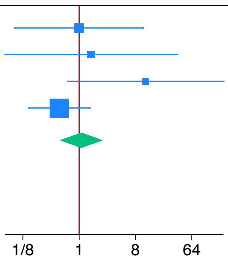

Heterogeneity: I^2 = 36.15%, H^2 = 1.57

Test of $\theta_i = \theta_j$: Q(3) = 4.70, p = 0.20

Test of θ = 0: z = 0.20, p = 0.84

Fixed-effects Mantel–Haenszel model

Supplementary Figure 46: Meta-analysis of venlafaxine versus placebo on tachycardia

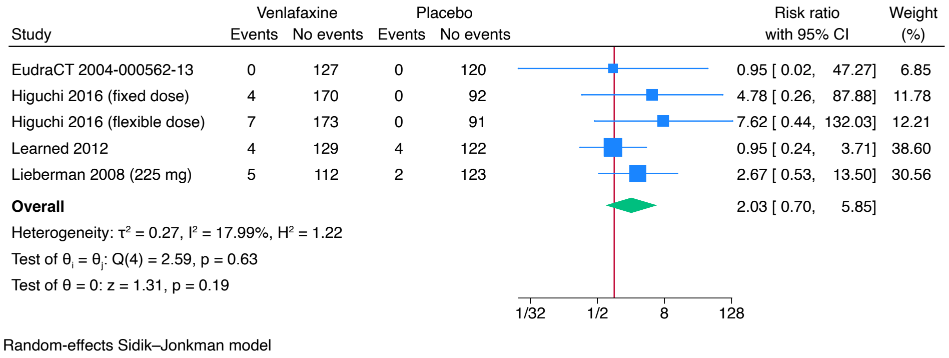

Supplementary Figure 47: Meta-analysis of venlafaxine versus placebo on urinary frequency

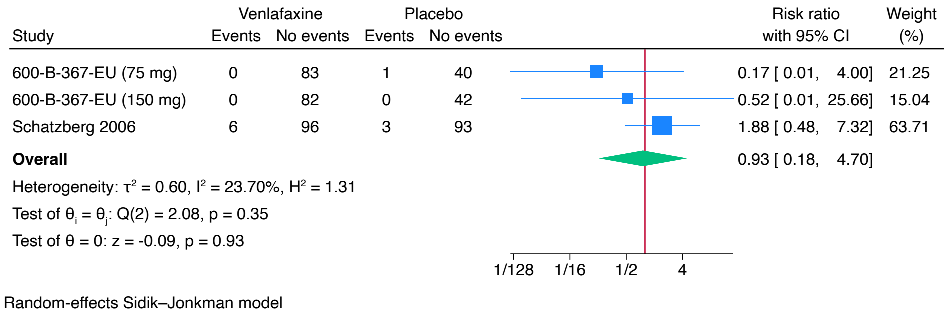

Supplementary Figure 48: Meta-analysis of venlafaxine versus placebo on vertigo

Study	Venlafaxine Events	Venlafaxine No events	Placebo Events	Placebo No events	Risk ratio with 95% CI	Weight (%)
600-B-367-EU (75 mg)	2	81	1	40	0.99 [0.09, 10.58]	11.39
600-B-367-EU (150 mg)	2	80	1	41	1.02 [0.10, 10.97]	11.38
EudraCT 2004-000562-13	9	118	5	115	1.70 [0.59, 4.93]	43.14
Guelfi 1995	0	46	1	46	0.34 [0.01, 8.15]	6.58
Higuchi 2016 (fixed dose)	4	170	1	90	2.09 [0.24, 18.44]	13.32
Higuchi 2016 (flexible dose)	6	174	1	91	3.07 [0.37, 25.09]	14.19
**Overall**					1.52 [0.66, 3.49]	

Heterogeneity: $\tau^2 = 0.12$, $I^2 = 10.59\%$, $H^2 = 1.12$

Test of $\theta_i = \theta_j$: Q(5) = 1.64, p = 0.90

Test of $\theta = 0$: z = 0.98, p = 0.33

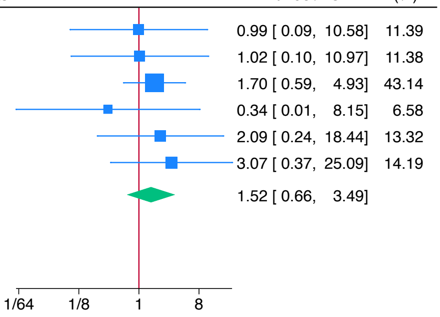

Random-effects Sidik–Jonkman model

Supplementary Figure 49: Meta-analysis of venlafaxine versus placebo on vomiting

Study	Venlafaxine Events	Venlafaxine No events	Placebo Events	Placebo No events	Risk ratio with 95% CI	Weight (%)
600-B-367-EU (75 mg)	2	81	1	40	0.99 [0.09, 10.58]	6.32
600-B-367-EU (150 mg)	3	79	1	41	1.54 [0.16, 14.32]	6.90
Alvarez 2012	4	109	1	104	3.72 [0.42, 32.72]	7.15
EudraCT 2004-000562-13	3	124	2	118	1.42 [0.24, 8.34]	9.34
Guelfi 1995	0	46	2	45	0.20 [0.01, 4.14]	4.37
Higuchi 2016 (fixed dose)	5	169	3	89	0.88 [0.22, 3.61]	12.00
Higuchi 2016 (flexible dose)	4	176	4	87	0.51 [0.13, 1.98]	12.39
Lieberman 2008 (225 mg)	3	114	0	125	7.47 [0.39, 143.17]	4.51
Nemeroff 2007	11	89	2	100	5.61 [1.28, 24.67]	11.41
Schatzberg 2006	9	93	2	94	4.24 [0.94, 19.11]	11.21
Sheehan 2009	9	86	4	91	2.25 [0.72, 7.06]	14.41
**Overall**					1.74 [0.86, 3.49]	

Heterogeneity: $\tau^2 = 0.54$, $I^2 = 41.12\%$, $H^2 = 1.70$

Test of $\theta_i = \theta_j$: $Q(10) = 11.62$, $p = 0.31$

Test of $\theta = 0$: $z = 1.55$, $p = 0.12$

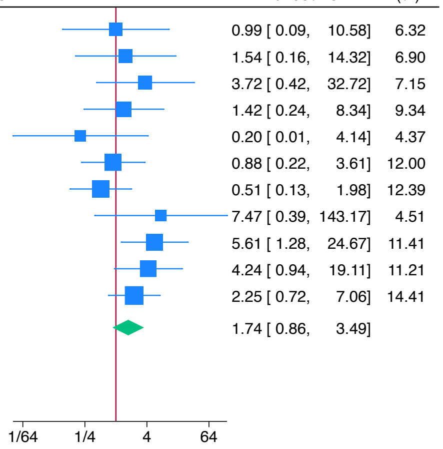

Random-effects Sidik–Jonkman model

Supplementary Figure 50: Meta-analysis of venlafaxine versus placebo on weight loss

Study	Venlafaxine Events	Venlafaxine No events	Placebo Events	Placebo No events	Risk ratio with 95% CI	Weight (%)
600-B-367-EU (75 mg)	2	81	0	41	2.50 [0.12, 50.91]	26.35
600-B-367-EU (150 mg)	1	81	0	42	1.55 [0.06, 37.35]	23.69
Guelfi 1995	2	44	0	47	5.11 [0.25, 103.55]	26.42
Schatzberg 2006	1	101	0	96	2.83 [0.12, 68.52]	23.55
**Overall**					2.78 [0.59, 13.11]	

Heterogeneity: $\tau^2 = 0.02$, $I^2 = 0.64\%$, $H^2 = 1.01$

Test of $\theta_i = \theta_j$: Q(3) = 0.29, p = 0.96

Test of $\theta = 0$: z = 1.29, p = 0.20

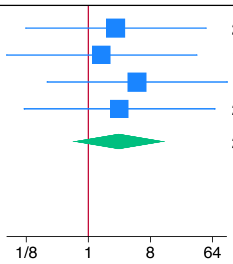

Random-effects Sidik–Jonkman model

Supplementary Figure 51: Meta-analysis of venlafaxine versus placebo on abnormality of accommodation

Study	Venlafaxine Events	Venlafaxine No events	Placebo Events	Placebo No events	Risk ratio with 95% CI	Weight (%)
600-B-367-EU (75 mg)	0	83	0	41	0.50 [0.01, 24.76]	9.16
600-B-367-EU (150 mg)	2	80	0	42	2.59 [0.13, 52.76]	14.93
Cunningham 1994	7	65	3	73	2.46 [0.66, 9.16]	60.94
Guelfi 1995	2	44	0	47	5.11 [0.25, 103.55]	14.97
**Overall**					2.39 [0.72, 7.99]	

Heterogeneity: $\tau^2 = 0.17$, $I^2 = 8.94\%$, $H^2 = 1.10$

Test of $\theta_i = \theta_j$: Q(3) = 0.87, p = 0.83

Test of $\theta = 0$: z = 1.42, p = 0.16

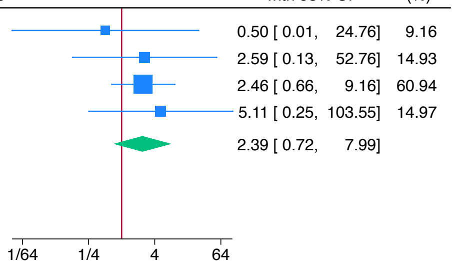

Random-effects Sidik–Jonkman model

Supplementary Figure 52: Meta-analysis of venlafaxine versus placebo on pruritis

Study	Venlafaxine Events	Venlafaxine No events	Placebo Events	Placebo No events	Risk ratio with 95% CI	Weight (%)
600-B-367-EU (75 mg)	2	81	0	41	2.50 [0.12, 50.91]	25.05
600-B-367-EU (150 mg)	1	81	0	42	1.55 [0.06, 37.35]	23.50
Guelfi 1995	2	44	0	47	5.11 [0.25, 103.55]	25.09
Schatzberg 2006	0	102	5	91	0.09 [0.00, 1.53]	26.37
**Overall**					1.10 [0.15, 7.89]	

Heterogeneity: $\tau^2 = 1.68$, $I^2 = 41.43\%$, $H^2 = 1.71$

Test of $\theta_i = \theta_j$: Q(3) = 4.34, p = 0.23

Test of $\theta = 0$: z = 0.09, p = 0.93

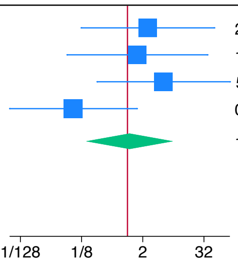

Random-effects Sidik–Jonkman model

Supplementary Figure 53: Meta-analysis of venlafaxine versus placebo on vasodilation

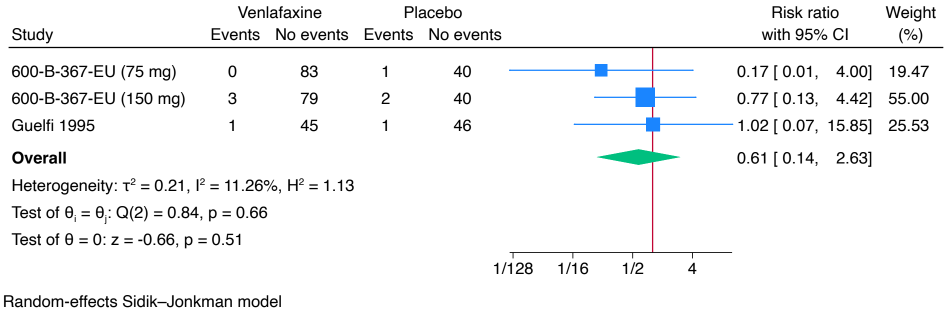

Supplementary Figure 54: Meta-analysis of venlafaxine versus placebo on neck pain

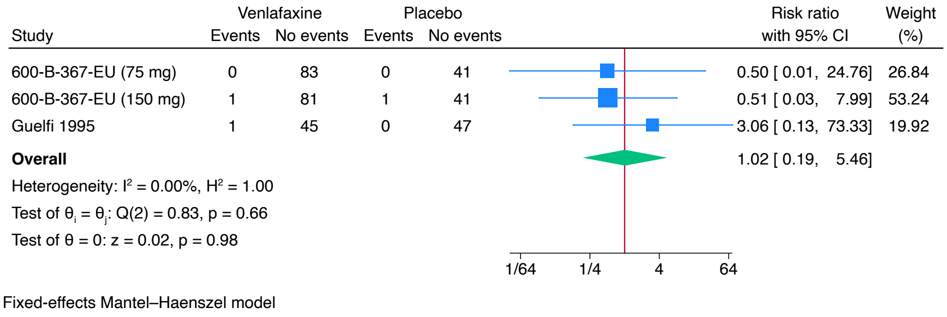

Supplementary Figure 55: Meta-analysis of venlafaxine versus placebo on pain

Study	Venlafaxine Events	Venlafaxine No events	Placebo Events	Placebo No events	Risk ratio with 95% CI	Weight (%)
600-B-367-EU (75 mg)	1	82	1	40	0.49 [0.03, 7.70]	30.78
600-B-367-EU (150 mg)	3	79	1	41	1.54 [0.16, 14.32]	42.83
Guelfi 1995	2	44	0	47	5.11 [0.25, 103.55]	26.39
**Overall**					1.49 [0.28, 7.93]	

Heterogeneity: $\tau^2 = 0.40$, $I^2 = 18.16\%$, $H^2 = 1.22$

Test of $\theta_i = \theta_j$: Q(2) = 1.26, p = 0.53

Test of $\theta = 0$: z = 0.47, p = 0.64

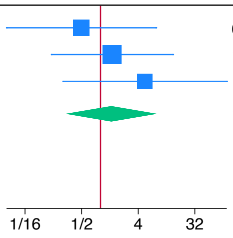

Random-effects Sidik–Jonkman model

Supplementary Figure 56: Meta-analysis of venlafaxine versus placebo on increased salivation

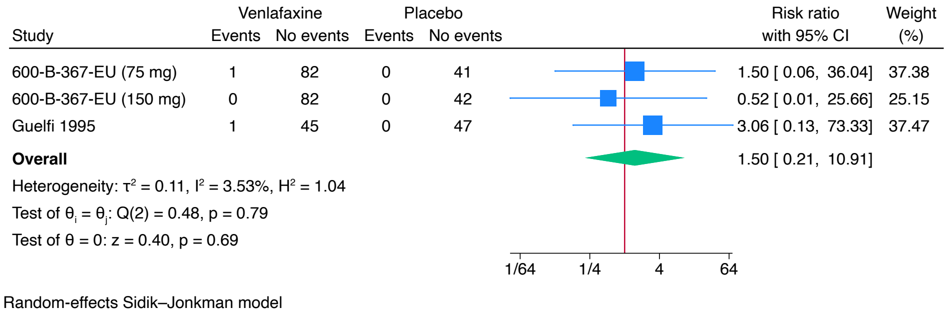

Supplementary Figure 57: Meta-analysis of venlafaxine versus placebo on tongue discolouration

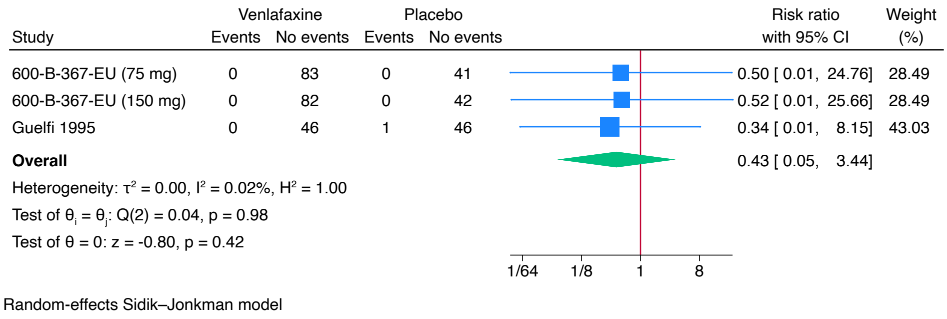

Supplementary Figure 58: Meta-analysis of venlafaxine versus placebo on hypochromic anaemia

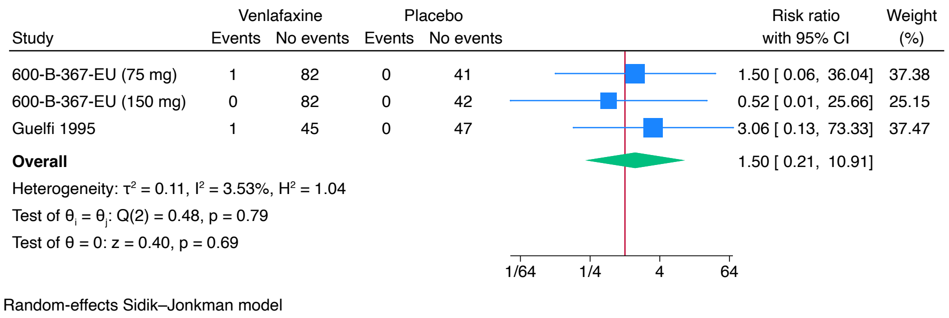

Supplementary Figure 59: Meta-analysis of venlafaxine versus placebo on hypercholesterolemia

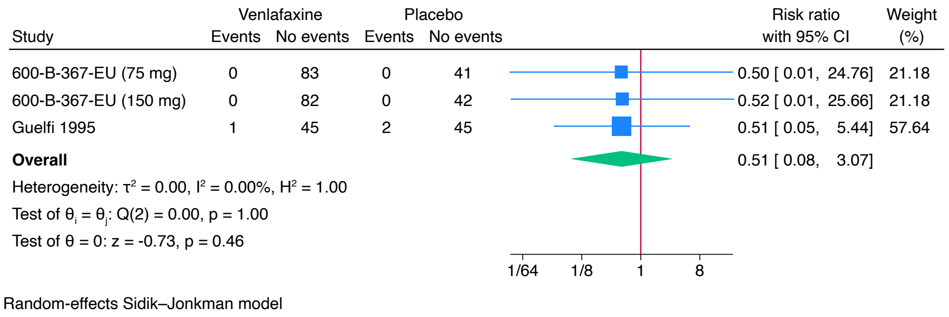

Supplementary Figure 60: Meta-analysis of venlafaxine versus placebo on bronchitis

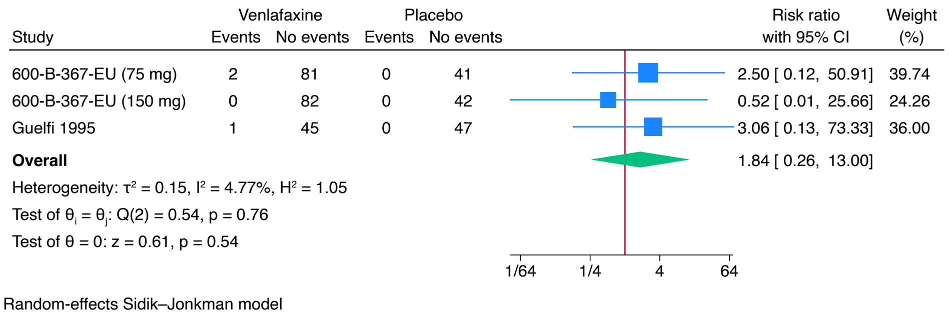

Supplementary Figure 61: Meta-analysis of venlafaxine versus placebo on pharyngitis

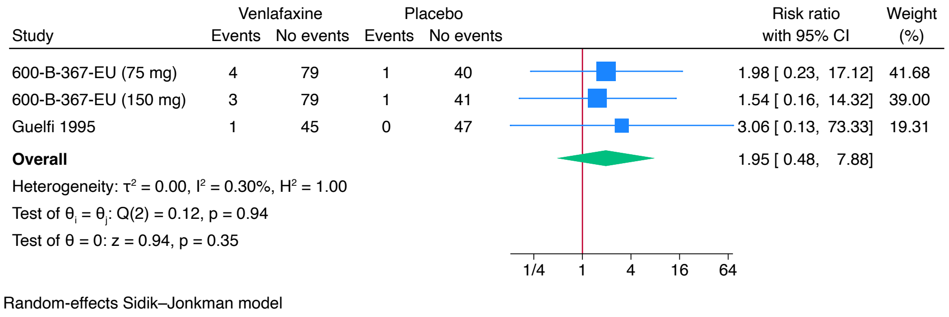

Supplementary Figure 62: Meta-analysis of venlafaxine versus placebo on urinary tract infection

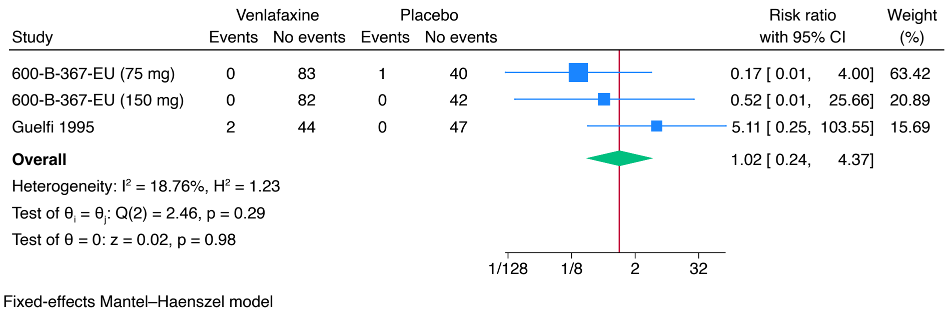

Supplementary Figure 63: Meta-analysis of venlafaxine versus placebo on urine abnormality

Study	Venlafaxine Events	Venlafaxine No events	Placebo Events	Placebo No events	Risk ratio with 95% CI	Weight (%)
600-B-367-EU (75 mg)	0	83	0	41	0.50 [0.01, 24.76]	28.80
600-B-367-EU (150 mg)	0	82	0	42	0.52 [0.01, 25.66]	28.46
Guelfi 1995	1	45	1	46	1.02 [0.07, 15.85]	42.74
**Overall**					0.73 [0.11, 4.95]	

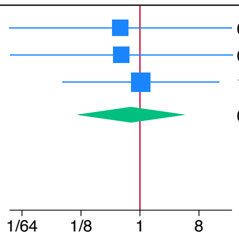

Heterogeneity: $I^2 = 0.00\%$, $H^2 = 1.00$

Test of $\theta_i = \theta_j$: Q(2) = 0.12, p = 0.94

Test of $\theta = 0$: z = -0.32, p = 0.75

Fixed-effects Mantel–Haenszel model

STATA®

Supplementary Figure 64: Meta-analysis of venlafaxine versus placebo on taste alteration

Study	Venlafaxine Events	Venlafaxine No events	Placebo Events	Placebo No events	Risk ratio with 95% CI	Weight (%)
600-B-367-EU (75 mg)	0	83	1	40	0.17 [0.01, 4.00]	63.42
600-B-367-EU (150 mg)	0	82	0	42	0.52 [0.01, 25.66]	20.89
Guelfi 1995	2	44	0	47	5.11 [0.25, 103.55]	15.69
**Overall**					1.02 [0.24, 4.37]	

Heterogeneity: $I^2 = 18.76\%$, $H^2 = 1.23$

Test of $\theta_i = \theta_j$: Q(2) = 2.46, p = 0.29

Test of $\theta = 0$: z = 0.02, p = 0.98

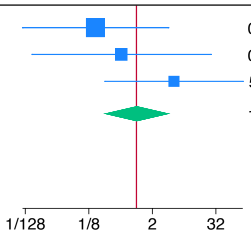

Fixed-effects Mantel–Haenszel model

Supplementary Figure 65: Meta-analysis of venlafaxine versus placebo on HDRS-17

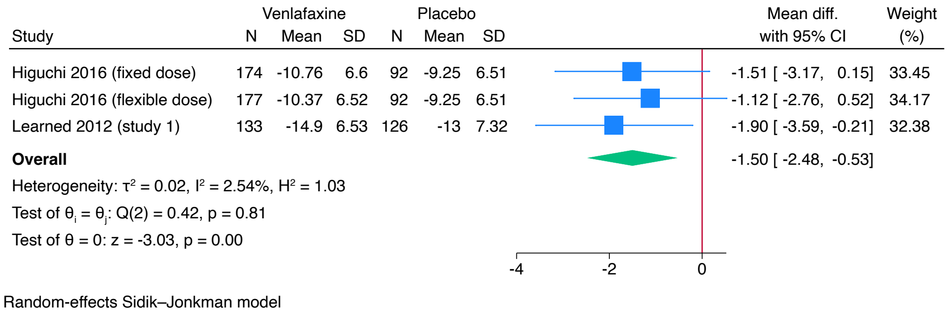

Supplementary Figure 66: Meta-analysis of venlafaxine versus placebo on suicidal ideation

Study	Venlafaxine Events	Venlafaxine No events	Placebo Events	Placebo No events	Risk ratio with 95% CI	Weight (%)
600-B-367-EU (150 mg)	1	81	0	42	1.55 [0.06, 37.35]	1.76
600-B-367-EU (75 mg)	0	84	0	41	0.49 [0.01, 24.47]	1.17
Hewett 2010	0	198	1	186	0.31 [0.01, 7.68]	1.74
Higuchi 2016 (fixed dose)	41	132	19	72	1.14 [0.70, 1.84]	44.19
Higuchi 2016 (flexible dose)	47	133	20	72	1.20 [0.76, 1.90]	46.55
Sheehan 2009	2	93	2	93	1.00 [0.14, 6.95]	4.59
**Overall**					1.13 [0.74, 1.73]	

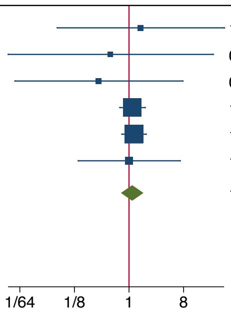

Heterogeneity: $\tau^2 = 0.05$, $I^2 = 15.80\%$, $H^2 = 1.19$

Test of $\theta_i = \theta_j$: $Q(5) = 0.90$, $p = 0.97$

Test of $\theta = 0$: $z = 0.56$, $p = 0.58$

Random-effects Sidik–Jonkman model

Supplementary Figure 67: Meta-analysis of venlafaxine versus placebo on MADRS

Study	Venlafaxine N	Venlafaxine Mean	Venlafaxine SD	Placebo N	Placebo Mean	Placebo SD	Mean diff. with 95% CI	Weight (%)
600-B-367-EU (75 mg)	53	8.2	9.5	27	9.4	7.3	-1.20 [-5.29, 2.89]	6.02
600-B-367-EU (150 mg)	48	7.6	9	26	9.4	7.3	-1.80 [-5.83, 2.23]	6.13
Alvarez 2012	95	-24.2	8.8	88	-16.6	9.4	-7.60 [-10.24, -4.96]	9.44
Guelfi 1995	36	13.6	10.5	23	19.1	13.2	-5.50 [-11.58, 0.58]	3.45
Hewett 2010	193	-17	10.56	186	-13.2	10.64	-3.80 [-5.93, -1.67]	10.96
Higuchi 2016 (fixed dose)	172	-15.3	10.1	91	-12.41	10.12	-2.89 [-5.46, -0.32]	9.64
Higuchi 2016 (flexible dose)	176	-15.05	10.08	91	-12.41	10.12	-2.64 [-5.19, -0.09]	9.68
Khan 1991 (75 mg)	23	21.3	9.7	9	25.3	11.7	-4.00 [-11.92, 3.92]	2.23
Khan 1991 (225 mg)	22	18.4	11.5	8	25.3	11.7	-6.90 [-16.25, 2.45]	1.67
Khan 1991 (375 mg)	22	16.4	9.2	9	25.3	11.7	-8.90 [-16.62, -1.18]	2.33
Mendels 1993 (25 mg)	78	-11.53	9.82	25	-10.53	8.98	-1.00 [-5.34, 3.34]	5.59
Mendels 1993 (50 - 75 mg)	72	-12.53	9.6	25	-10.53	8.98	-2.00 [-6.30, 2.30]	5.66
Mendels 1993 (150 - 200 mg)	77	-14.8	9.64	25	-10.53	8.98	-4.27 [-8.55, 0.01]	5.69
Sheehan 2009	53	13.53	10.31	55	18.98	11.78	-5.45 [-9.63, -1.27]	5.86
Thase 1997	91	15.2	1.14	100	20.6	1.08	-5.40 [-5.71, -5.09]	15.67
**Overall**							-4.03 [-5.30, -2.75]	

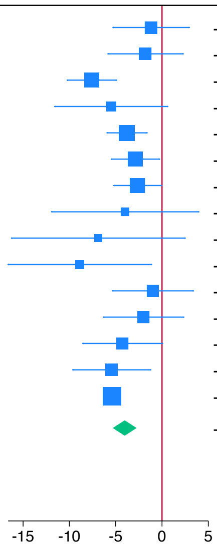

Heterogeneity: $\tau^2 = 2.68$, $I^2 = 60.01\%$, $H^2 = 2.50$

Test of $\theta_i = \theta_j$: Q(14) = 26.70, p = 0.02

Test of $\theta = 0$: z = -6.19, p = 0.00

Random-effects Sidik–Jonkman model

Supplementary Figure 68: Meta-analysis of venlafaxine versus placebo on suicides or suicide attempts (sensitivity analysis)

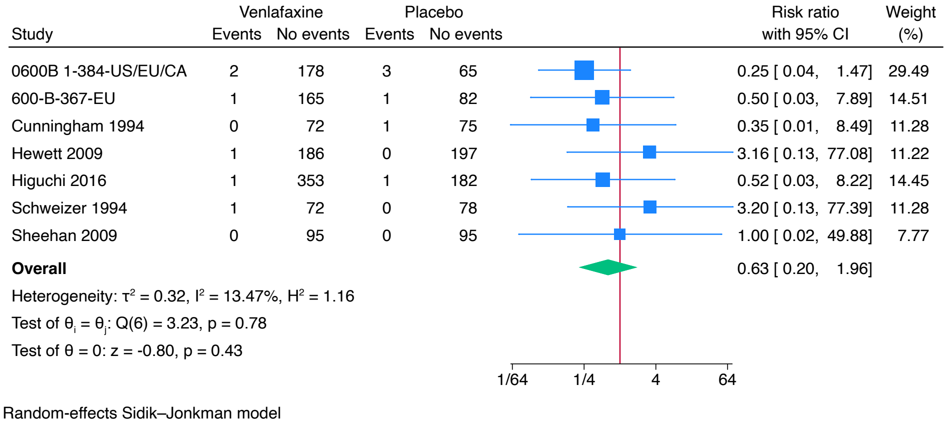

Supplementary Figure 69: Meta-analysis of venlafaxine versus placebo on serious adverse events (sensitivity analysis)

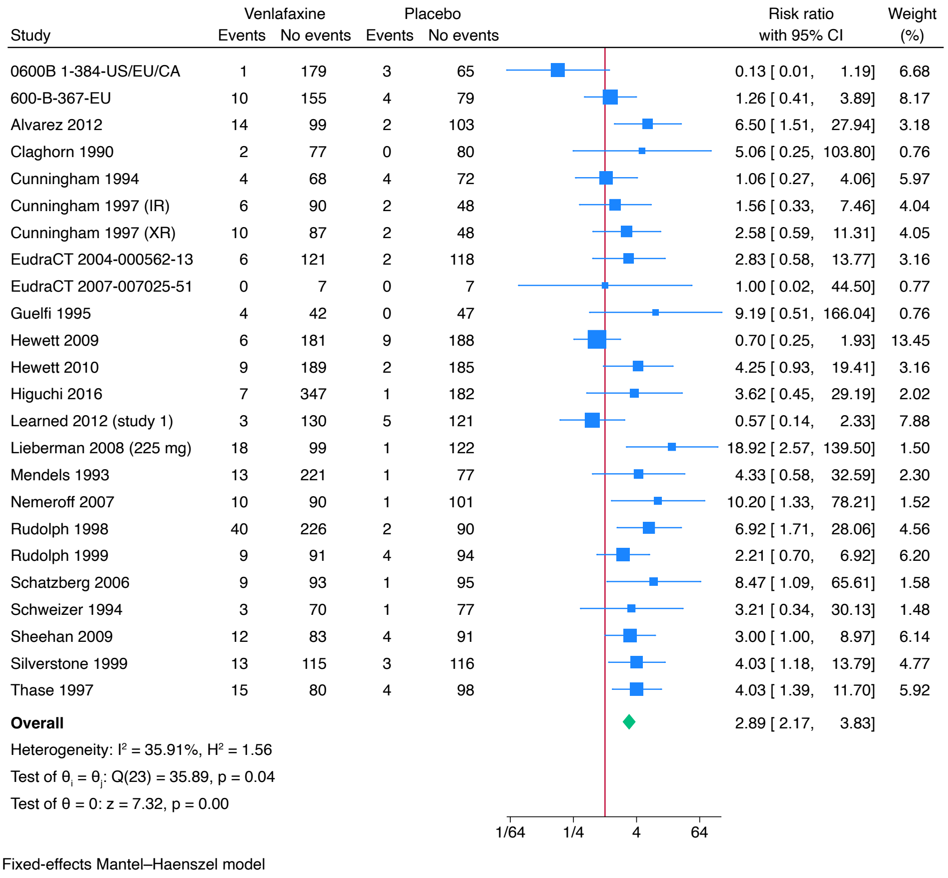

Supplementary Figure 70: Meta-analysis of venlafaxine versus placebo on sexual dysfunction (sensitivity analysis)

Study	Venlafaxine Events	Venlafaxine No events	Placebo Events	Placebo No events	Risk ratio with 95% CI	Weight (%)
600-B-367-EU	2	52	0	26	2.45 [0.12, 49.36]	8.35
Alvarez 2012	14	99	2	103	6.50 [1.51, 27.94]	25.80
Cunningham 1997 (IR)	2	29	0	20	3.28 [0.17, 64.99]	7.51
Cunningham 1997 (XR)	10	27	0	21	12.16 [0.75, 197.55]	7.88
EudraCT 2004-000562-13	2	33	0	40	5.69 [0.28, 114.74]	5.82
Lieberman 2008 (225 mg)	4	32	1	44	5.00 [0.58, 42.80]	11.06
Mendels 1993	11	223	0	78	7.73 [0.46, 129.71]	9.31
Schatzberg 2006	9	93	1	95	8.47 [1.09, 65.61]	12.82
Thase 1997	8	27	1	40	9.37 [1.23, 71.31]	11.46
**Overall**					6.85 [3.22, 14.58]	

Heterogeneity: $I^2 = 0.00\%$, $H^2 = 1.00$

Test of $\theta_i = \theta_j$: $Q(8) = 1.09$, $p = 1.00$

Test of $\theta = 0$: $z = 5.00$, $p = 0.00$

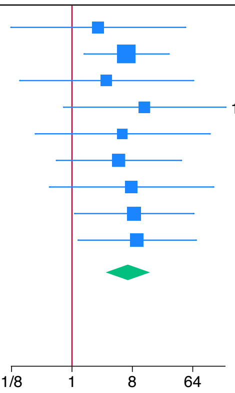

Fixed-effects Mantel–Haenszel model

Supplementary Figure 71: Meta-analysis of venlafaxine versus placebo on anorexia (sensitivity analysis)

Study	Venlafaxine Events	Venlafaxine No events	Placebo Events	Placebo No events	Risk ratio with 95% CI	Weight (%)
600-B-367-EU	2	163	2	81	0.50 [0.07, 3.51]	9.86
Cunningham 1997 (IR)	6	90	2	48	1.56 [0.33, 7.46]	9.74
Cunningham 1997 (XR)	10	87	2	48	2.58 [0.59, 11.31]	9.78
EudraCT 2004-000562-13	6	121	2	118	2.83 [0.58, 13.77]	7.62
Hewett 2010	9	189	2	185	4.25 [0.93, 19.41]	7.62
Lieberman 2008 (225 mg)	18	99	1	122	18.92 [2.57, 139.50]	3.61
Rudolph 1998	40	226	2	90	6.92 [1.71, 28.06]	11.01
Rudolph 1999	9	91	4	94	2.21 [0.70, 6.92]	14.96
Silverstone 1999	13	115	3	116	4.03 [1.18, 13.79]	11.52
Thase 1997	15	80	4	98	4.03 [1.39, 11.70]	14.29
**Overall**					3.81 [2.47, 5.86]	

Heterogeneity: $I^2 = 9.16\%$, $H^2 = 1.10$

Test of $\theta_i = \theta_j$: $Q(9) = 9.91$, $p = 0.36$

Test of $\theta = 0$: $z = 6.07$, $p = 0.00$

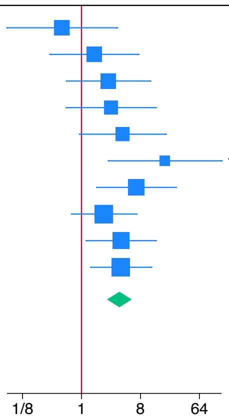

Fixed-effects Mantel–Haenszel model

Supplementary Figure 72: Meta-analysis of venlafaxine versus placebo on anxiety (sensitivity analysis)

Study	Venlafaxine Events	Venlafaxine No events	Placebo Events	Placebo No events	Risk ratio with 95% CI	Weight (%)
600-B-367-EU	2	163	2	81	0.50 [0.07, 3.51]	9.04
EudraCT 2004-000562-13	0	127	1	119	0.32 [0.01, 7.66]	5.24
Hewett 2009	6	181	9	188	0.70 [0.25, 1.93]	29.78
Learned 2012	3	130	5	121	0.57 [0.14, 2.33]	17.45
Mendels 1993	13	221	1	77	4.33 [0.58, 32.59]	5.10
Nemeroff 2007	10	90	1	101	10.20 [1.33, 78.21]	3.36
Rudolph 1998	10	57	0	26	8.34 [0.51, 137.36]	2.43
Schatzberg 2006	2	100	4	92	0.47 [0.09, 2.51]	14.00
Sheehan 2009	12	83	4	91	3.00 [1.00, 8.97]	13.59
**Overall**					1.61 [1.01, 2.56]	

Heterogeneity: $I^2 = 49.27\%$, $H^2 = 1.97$

Test of $\theta_i = \theta_j$: Q(8) = 15.77, p = 0.05

Test of $\theta = 0$: z = 2.01, p = 0.04

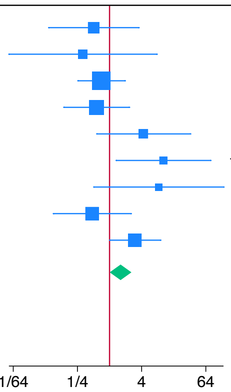

Fixed-effects Mantel–Haenszel model

Supplementary Figure 73: Meta-analysis of venlafaxine versus placebo on worsening of depression (sensitivity analysis)

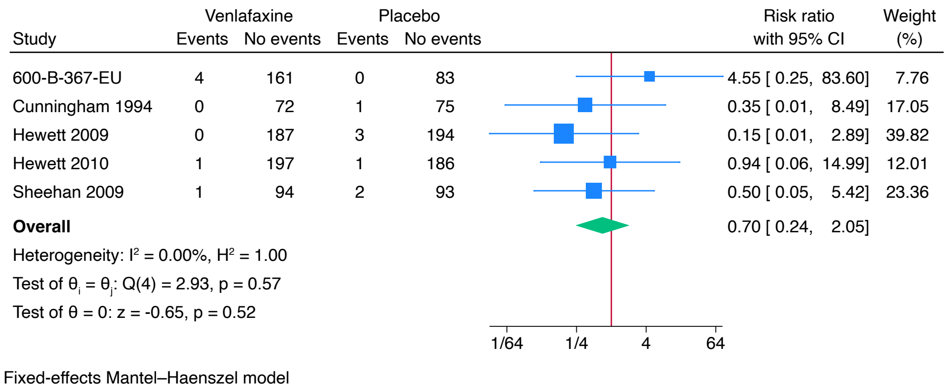

Supplementary Figure 74: Meta-analysis of venlafaxine versus placebo on hypertension (sensitivity analysis)

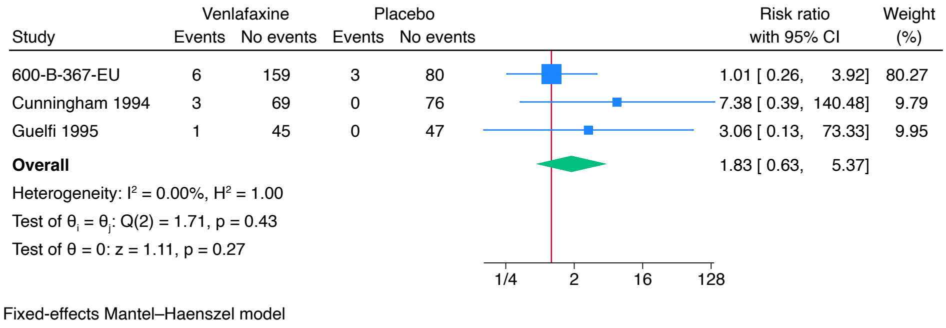

Supplementary Figure 75: Meta-analysis of venlafaxine versus placebo on hypotension (sensitivity analysis)

Study	Venlafaxine Events	Venlafaxine No events	Placebo Events	Placebo No events	Risk ratio with 95% CI	Weight (%)
600-B-367-EU	1	164	0	83	1.52 [0.06, 36.87]	33.49
Higuchi 2016	7	347	1	182	3.62 [0.45, 29.19]	66.51
**Overall**					2.92 [0.52, 16.42]	

Heterogeneity: $I^2 = 0.00\%$, $H^2 = 1.00$

Test of $\theta_i = \theta_j$: Q(1) = 0.20, p = 0.65

Test of $\theta = 0$: z = 1.21, p = 0.23

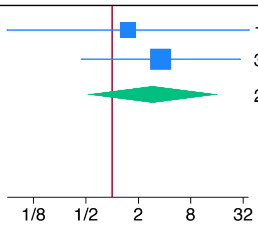

Fixed-effects Mantel–Haenszel model

Supplementary Figure 76: Meta-analysis of venlafaxine versus placebo on discontinuation symptoms (sensitivity analysis)

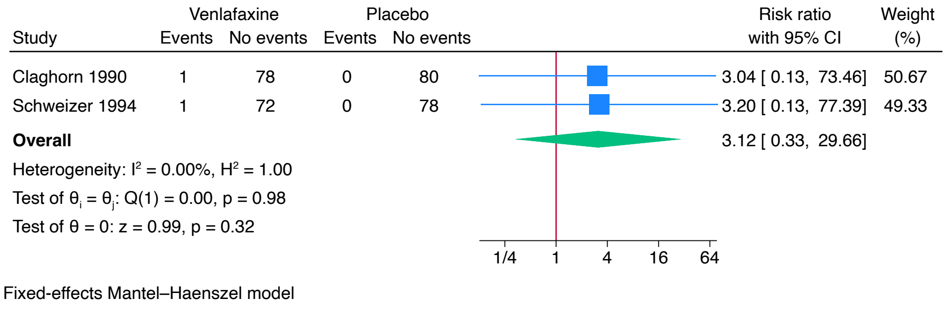

Supplementary Figure 77: Meta-analysis of venlafaxine versus placebo on fall (sensitivity analysis)

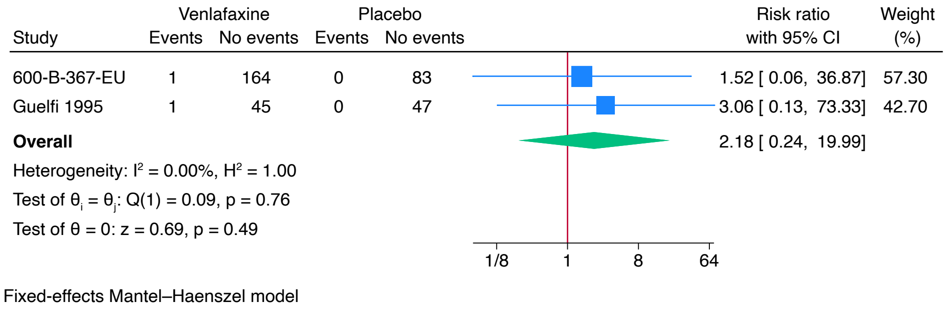

Supplementary Figure 78: Meta-analysis of venlafaxine versus placebo on intentional overdose (sensitivity analysis)

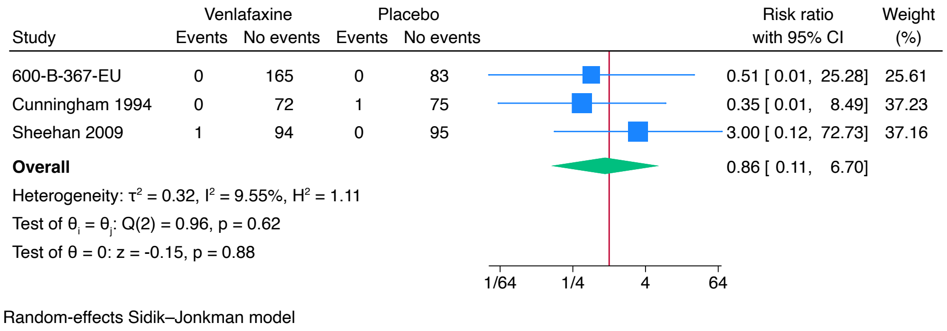

Supplementary Figure 79: Meta-analysis of venlafaxine versus placebo on QTc (sensitivity analysis)

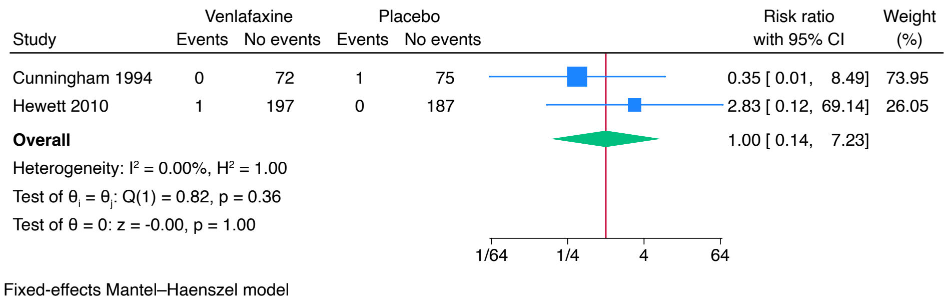

Supplementary Figure 80: Meta-analysis of venlafaxine versus placebo on syncope (sensitivity analysis)

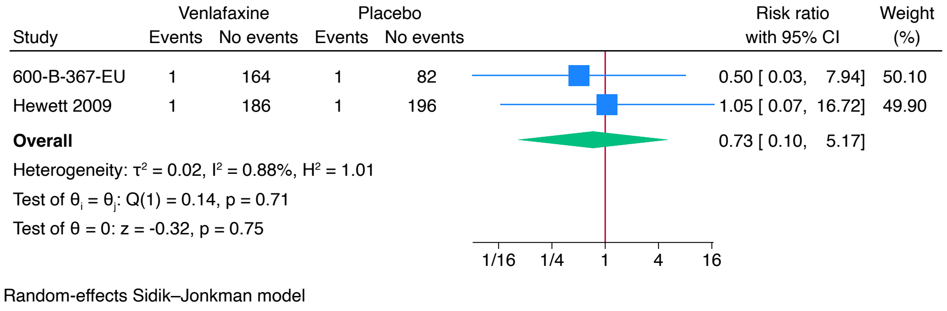

Supplementary Figure 81: Meta-analysis of venlafaxine versus placebo on non-serious adverse events (sensitivity analysis)

Study	Venlafaxine Events	Venlafaxine No events	Placebo Events	Placebo No events	Risk ratio with 95% CI	Weight (%)
600-B-367-EU (75 mg)	56	27	25	16	1.11 [0.83, 1.47]	2.79
600-B-367-EU (150 mg)	53	29	25	17	1.09 [0.81, 1.46]	2.76
Alvarez 2012	85	28	64	41	1.23 [1.02, 1.49]	5.54
Claghorn 1990	72	7	59	21	1.24 [1.07, 1.43]	4.89
Cunningham 1994	71	1	56	20	1.34 [1.17, 1.53]	4.55
Cunningham 1997 (IR)	43	53	5	45	4.48 [1.89, 10.59]	0.55
Cunningham 1997 (XR)	44	53	5	45	4.54 [1.92, 10.72]	0.55
EudraCT 2004-000562-13	92	35	77	43	1.13 [0.95, 1.34]	6.61
EudraCT 2007-007025-51	4	3	4	3	1.00 [0.40, 2.48]	0.33
Guelfi 1995	36	10	22	25	1.67 [1.19, 2.35]	1.82
Hewett 2009	93	94	95	102	1.03 [0.84, 1.26]	7.72
Hewett 2010	133	65	112	75	1.12 [0.96, 1.31]	9.61
Higuchi 2016 (fixed dose)	131	43	61	30	1.12 [0.95, 1.33]	6.68
Higuchi 2016 (flexible dose)	147	33	62	30	1.21 [1.03, 1.42]	6.85
Khan 1991 (75 mg)	23	0	5	3	1.60 [0.95, 2.71]	0.67
Khan 1991 (225 mg)	22	0	6	3	1.51 [0.95, 2.38]	0.76
Khan 1991 (375 mg)	21	1	6	3	1.43 [0.89, 2.29]	0.71
Khan 1998 (75 mg)	24	72	2	31	4.12 [1.03, 16.52]	0.25
Khan 1998 (150 mg)	19	77	2	31	3.27 [0.80, 13.27]	0.25
Khan 1998 (200 mg)	26	68	2	30	4.43 [1.11, 17.61]	0.25
Learned 2012 (study 1)	94	39	77	49	1.16 [0.97, 1.38]	6.60
Lieberman 2008 (225 mg)	27	90	16	109	1.80 [1.03, 3.17]	1.29
Luthringer 1996	5	7	6	6	0.83 [0.35, 2.00]	0.50
Mendels 1993 (25 mg)	20	59	7	19	0.94 [0.45, 1.97]	0.88
Mendels 1993 (50 - 75 mg)	19	57	7	19	0.93 [0.44, 1.95]	0.87
Mendels 1993 (150 - 200 mg)	17	52	7	19	0.92 [0.43, 1.95]	0.85
Nemeroff 2007	40	60	8	94	5.10 [2.51, 10.34]	0.66
Rudolph 1999	36	64	14	84	2.52 [1.45, 4.37]	1.18
Schatzberg 2006	94	8	83	13	1.07 [0.97, 1.17]	7.13
Schweizer 1991 (75, 225 and 375 mg)	24	20	4	12	2.18 [0.90, 5.32]	0.49
Schweizer 1994	63	10	64	14	1.05 [0.92, 1.21]	5.16
Sheehan 2009	87	8	85	10	1.02 [0.93, 1.12]	7.09
Silverstone 1999	49	79	20	99	2.28 [1.44, 3.59]	1.73
Thase 1997	34	61	18	84	2.03 [1.23, 3.34]	1.45
**Overall**					1.29 [1.23, 1.35]	

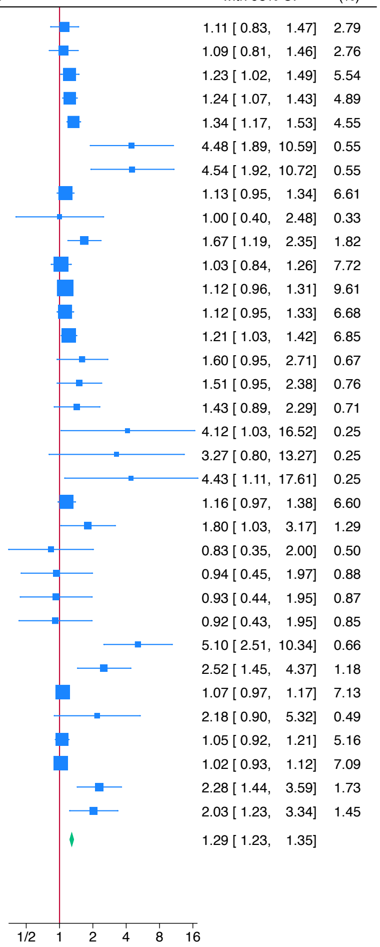

Heterogeneity: $I^2 = 74.17\%$, $H^2 = 3.87$

Test of $\theta_i = \theta_j$: $Q(33) = 127.76$, $p = 0.00$

Test of $\theta = 0$: $z = 10.63$, $p = 0.00$

Fixed-effects Mantel–Haenszel model

Supplementary Figure 82: Meta-analysis of venlafaxine versus placebo on nausea (sensitivity analysis)

Graph 17/03/2024, 18.33

Study	Venlafaxine Events	Venlafaxine No events	Placebo Events	Placebo No events	Risk ratio with 95% CI	Weight (%)
600-B-367-EU (75 mg)	15	68	3	38	2.47 [0.76, 8.05]	1.34
600-B-367-EU (150 mg)	22	60	2	40	5.63 [1.39, 22.82]	0.88
Alvarez 2012	38	75	10	95	3.53 [1.85, 6.72]	3.45
Claghorn 1990	35	44	7	73	5.06 [2.39, 10.71]	2.31
Cunningham 1994	32	40	4	72	8.44 [3.14, 22.68]	1.29
Cunningham 1997 (IR)	43	53	5	45	4.48 [1.89, 10.59]	2.19
Cunningham 1997 (XR)	44	53	5	45	4.54 [1.92, 10.72]	2.19
EudraCT 2004-000562-13	27	100	14	106	1.82 [1.00, 3.30]	4.79
EudraCT 2007-007025-51	0	7	0	7	1.00 [0.02, 44.50]	0.17
Guelfi 1995	8	38	1	46	8.17 [1.06, 62.78]	0.33
Hewett 2009	36	151	21	176	1.81 [1.10, 2.98]	6.80
Hewett 2010	53	145	16	171	3.13 [1.86, 5.27]	5.47
Higuchi 2016 (fixed dose)	39	135	12	80	1.72 [0.95, 3.12]	5.22
Higuchi 2016 (flexible dose)	53	127	12	79	2.23 [1.26, 3.96]	5.30
Khan 1998 (75 mg)	8	88	1	32	2.75 [0.36, 21.17]	0.49
Khan 1998 (150 mg)	7	89	0	33	5.26 [0.31, 89.63]	0.25
Khan 1998 (200 mg)	16	78	0	32	11.46 [0.71, 185.79]	0.25
Learned 2012	29	104	14	112	1.96 [1.09, 3.54]	4.78
Lieberman 2008 (225 mg)	34	83	16	109	2.27 [1.33, 3.89]	5.14
Mendels 1993 (25 mg)	22	57	3	23	2.41 [0.79, 7.41]	1.50
Mendels 1993 (50 - 75 mg)	19	57	2	24	3.25 [0.81, 13.01]	0.99
Mendels 1993 (150 - 200 mg)	23	56	2	24	3.78 [0.96, 14.97]	1.00
Nemeroff 2007	40	60	8	94	5.10 [2.51, 10.34]	2.63
Rudolph 1998 (75 mg)	29	60	4	27	2.53 [0.96, 6.61]	1.97
Rudolph 1998 (225 mg)	34	55	4	27	2.96 [1.14, 7.67]	1.97
Rudolph 1998 (375 mg)	51	37	5	25	3.48 [1.53, 7.89]	2.48
Rudolph 1999	36	64	14	84	2.52 [1.45, 4.37]	4.70
Schatzberg 2006	46	56	13	83	3.33 [1.92, 5.77]	4.45
Schweizer 1994	20	53	11	67	1.94 [1.00, 3.77]	3.54
Sheehan 2009	36	59	15	80	2.40 [1.41, 4.08]	4.99
Silverstone 1999	52	76	33	86	1.46 [1.02, 2.10]	11.37
Thase 1997	34	61	18	84	2.03 [1.23, 3.34]	5.77
**Overall**					2.68 [2.37, 3.04]	

Heterogeneity: $I^2 = 25.23\%$, $H^2 = 1.34$

Test of $\theta_i = \theta_j$: Q(31) = 41.46, p = 0.10

Test of $\theta = 0$: z = 15.54, p = 0.00

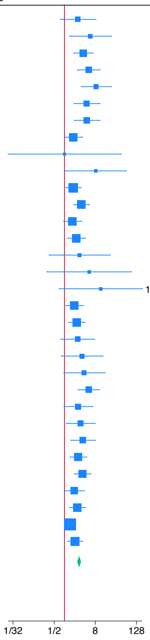

Fixed-effects Mantel–Haenszel model

Supplementary Figure 83: Meta-analysis of venlafaxine versus placebo on dry mouth (sensitivity analysis)

Graph 17/03/2024, 17.54

Study	Venlafaxine Events	Venlafaxine No events	Placebo Events	Placebo No events	Risk ratio with 95% CI	Weight (%)
600-B-367-EU (75 mg)	5	78	0	41	5.50 [0.31, 97.13]	0.37
600-B-367-EU (150 mg)	8	74	0	42	8.81 [0.52, 148.99]	0.36
Alvarez 2012	19	94	7	98	2.52 [1.11, 5.75]	4.00
Claghorn 1990	19	60	12	68	1.60 [0.84, 3.08]	6.57
Cunningham 1994	21	51	5	71	4.43 [1.77, 11.13]	2.68
Cunningham 1997 (IR)	21	75	4	46	2.73 [0.99, 7.53]	2.90
Cunningham 1997 (XR)	16	81	4	46	2.06 [0.73, 5.84]	2.91
EudraCT 2004-000562-13	17	110	1	119	16.06 [2.17, 118.84]	0.57
EudraCT 2007-007025-51	1	6	0	7	3.00 [0.14, 63.15]	0.28
Guelfi 1995	3	43	3	44	1.02 [0.22, 4.80]	1.64
Hewett 2009	13	174	11	186	1.25 [0.57, 2.71]	5.91
Hewett 2010	35	163	13	174	2.54 [1.39, 4.65]	7.37
Higuchi 2016 (fixed dose)	5	169	1	90	2.61 [0.31, 22.05]	0.72
Higuchi 2016 (flexible dose)	7	173	0	92	7.71 [0.45, 133.48]	0.36
Learned 2012	12	121	5	121	2.27 [0.82, 6.27]	2.83
Lieberman 2008 (225 mg)	30	87	9	116	3.56 [1.77, 7.18]	4.80
Mendels 1993 (25 mg)	14	65	4	22	1.15 [0.42, 3.19]	3.32
Mendels 1993 (50 - 75 mg)	16	60	3	23	1.82 [0.58, 5.76]	2.46
Mendels 1993 (150 - 200 mg)	23	56	3	23	2.52 [0.82, 7.72]	2.49
Nemeroff 2007	24	76	15	87	1.63 [0.91, 2.92]	8.19
Rudolph 1998 (75 mg)	16	73	4	27	1.39 [0.50, 3.85]	3.27
Rudolph 1998 (225 mg)	19	70	4	27	1.65 [0.61, 4.49]	3.27
Rudolph 1998 (375 mg)	17	71	4	26	1.45 [0.53, 3.97]	3.29
Schatzberg 2006	23	79	14	82	1.55 [0.85, 2.83]	7.95
Schweizer 1994	14	59	7	71	2.14 [0.91, 5.00]	3.73
Sheehan 2009	36	59	9	86	4.00 [2.04, 7.84]	4.96
Silverstone 1999	30	98	14	105	1.99 [1.11, 3.57]	8.00
Thase 1997	17	78	9	93	2.03 [0.95, 4.33]	4.79
**Overall**					2.26 [1.91, 2.67]	

Heterogeneity: $I^2 = 0.00\%$, $H^2 = 1.00$

Test of $\theta_i = \theta_j$: $Q(27) = 23.70$, $p = 0.65$

Test of $\theta = 0$: $z = 9.60$, $p = 0.00$

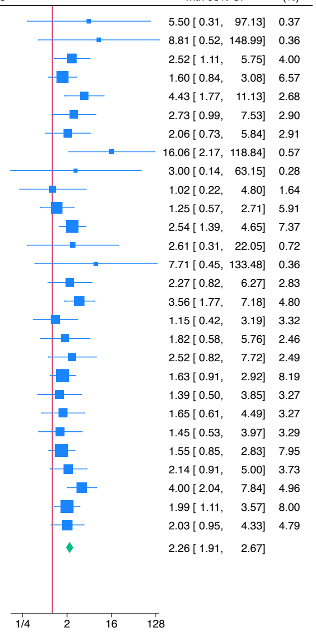

Fixed-effects Mantel–Haenszel model

Supplementary Figure 84: Meta-analysis of venlafaxine versus placebo on dizziness (sensitivity analysis)

Graph 17/03/2024, 18.34

Study	Venlafaxine Events	Venlafaxine No events	Placebo Events	Placebo No events	Risk ratio with 95% CI	Weight (%)
600-B-367-EU (75 mg)	15	68	2	39	3.70 [0.89, 15.44]	1.88
600-B-367-EU (150 mg)	8	74	2	40	2.05 [0.46, 9.22]	1.85
Alvarez 2012	14	99	8	97	1.63 [0.71, 3.72]	5.81
Claghorn 1990	15	64	5	75	3.04 [1.16, 7.96]	3.48
Cunningham 1994	12	60	4	72	3.17 [1.07, 9.37]	2.73
Cunningham 1997 (IR)	34	62	3	47	5.90 [1.91, 18.27]	2.77
Cunningham 1997 (XR)	28	69	3	47	4.81 [1.54, 15.05]	2.78
EudraCT 2004-000562-13	6	121	7	113	0.81 [0.28, 2.34]	5.05
Guelfi 1995	4	42	1	46	4.09 [0.47, 35.20]	0.69
Hewett 2009	9	178	14	183	0.68 [0.30, 1.53]	9.56
Hewett 2010	27	171	11	176	2.32 [1.18, 4.54]	7.93
Higuchi 2016 (fixed dose)	10	164	2	90	2.64 [0.59, 11.81]	1.83
Higuchi 2016 (flexible dose)	18	162	3	88	3.03 [0.92, 10.03]	2.79
Khan 1998 (75 mg)	5	91	1	32	1.72 [0.21, 14.18]	1.04
Khan 1998 (150 mg)	2	94	0	33	1.75 [0.09, 35.60]	0.52
Khan 1998 (200 mg)	6	88	0	32	4.52 [0.26, 77.99]	0.52
Learned 2012	15	118	11	115	1.29 [0.62, 2.70]	7.92
Mendels 1993 (25 mg)	7	72	3	23	0.77 [0.21, 2.76]	3.16
Mendels 1993 (50 - 75 mg)	17	59	2	24	2.91 [0.72, 11.74]	2.09
Mendels 1993 (150 - 200 mg)	12	67	2	24	1.97 [0.47, 8.25]	2.11
Nemeroff 2007	13	87	3	99	4.42 [1.30, 15.04]	2.08
Rudolph 1998 (75 mg)	17	72	1	30	5.92 [0.82, 42.67]	1.04
Rudolph 1998 (225 mg)	20	69	1	30	6.97 [0.98, 49.77]	1.04
Rudolph 1998 (375 mg)	21	67	2	28	3.58 [0.89, 14.37]	2.09
Rudolph 1999	26	74	7	91	3.64 [1.66, 7.99]	4.96
Schatzberg 2006	17	85	5	91	3.20 [1.23, 8.34]	3.61
Schweizer 1994	12	61	3	75	4.27 [1.26, 14.54]	2.03
Sheehan 2009	15	80	3	92	5.00 [1.50, 16.71]	2.10
Silverstone 1999	49	79	20	99	2.28 [1.44, 3.59]	14.53
**Overall**					2.54 [2.10, 3.08]	

Heterogeneity: $I^2 = 15.23\%$, $H^2 = 1.18$

Test of $\theta_i = \theta_j$: Q(28) = 33.03, p = 0.23

Test of $\theta = 0$: z = 9.57, p = 0.00

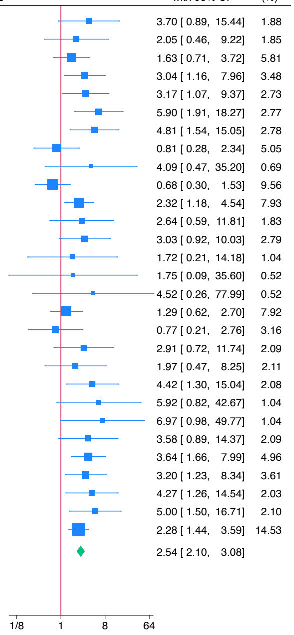

Fixed-effects Mantel–Haenszel model

Supplementary Figure 85: Meta-analysis of venlafaxine versus placebo on sweating (sensitivity analysis)

Study	Venlafaxine Events	Venlafaxine No events	Placebo Events	Placebo No events	Risk ratio with 95% CI	Weight (%)
600-B-367-EU (75 mg)	8	75	1	40	3.95 [0.51, 30.54]	2.02
600-B-367-EU (150 mg)	12	70	0	42	12.95 [0.79, 213.54]	0.99
Alvarez 2012	17	96	2	103	7.90 [1.87, 33.37]	3.12
Claghorn 1990	8	71	1	79	8.10 [1.04, 63.28]	1.50
Cunningham 1994	9	63	1	75	9.50 [1.23, 73.11]	1.47
Cunningham 1997 (IR)	13	83	1	49	6.77 [0.91, 50.28]	1.98
Cunningham 1997 (XR)	18	79	2	48	4.64 [1.12, 19.20]	3.97
EudraCT 2004-000562-13	12	115	4	116	2.83 [0.94, 8.55]	6.19
Guelfi 1995	7	39	1	46	7.15 [0.92, 55.86]	1.49
Hewett 2009	15	172	7	190	2.26 [0.94, 5.41]	10.27
Hewett 2010	16	182	7	180	2.16 [0.91, 5.13]	10.84
Higuchi 2016 (fixed dose)	3	171	1	91	1.59 [0.17, 15.03]	1.97
Higuchi 2016 (flexible dose)	15	165	1	90	7.58 [1.02, 56.51]	2.00
Learned 2012	15	118	3	123	4.74 [1.40, 15.97]	4.64
Lieberman 2008 (225 mg)	21	96	6	119	3.74 [1.56, 8.94]	8.74
Mendels 1993 (25 mg)	2	77	0	26	1.69 [0.08, 34.06]	1.13
Mendels 1993 (50 - 75 mg)	4	72	0	26	3.16 [0.18, 56.71]	1.11
Mendels 1993 (150 - 200 mg)	17	62	0	26	11.81 [0.73, 189.85]	1.13
Nemeroff 2007	14	86	2	100	7.14 [1.67, 30.61]	2.98
Rudolph 1999	10	90	3	95	3.27 [0.93, 11.51]	4.56
Schatzberg 2006	11	91	1	95	10.35 [1.36, 78.67]	1.55
Schweizer 1994	7	66	0	78	16.01 [0.93, 275.48]	0.73
Silverstone 1999	36	92	12	107	2.79 [1.52, 5.10]	18.73
Thase 1997	13	82	4	98	3.49 [1.18, 10.33]	5.81
Schweizer 1991	11	33	0	16	8.69 [0.54, 139.48]	1.09
**Overall**					4.16 [3.18, 5.44]	

Heterogeneity: I^2 = 0.00%, H^2 = 1.00

Test of $\theta_i = \theta_j$: Q(24) = 13.92, p = 0.95

Test of θ = 0: z = 10.41, p = 0.00

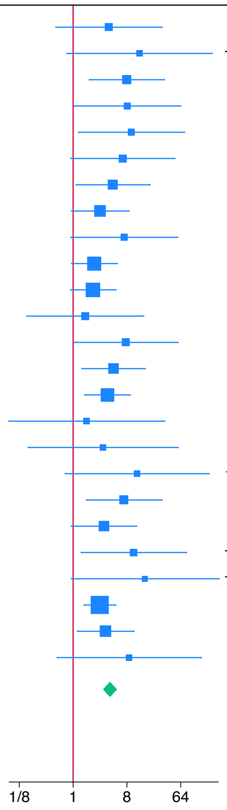

Fixed-effects Mantel–Haenszel model

Supplementary Figure 86: Meta-analysis of venlafaxine versus placebo on somnolence (sensitivity analysis)

Study	Venlafaxine Events	Venlafaxine No events	Placebo Events	Placebo No events	Risk ratio with 95% CI	Weight (%)
600-B-367-EU (75 mg)	6	77	1	40	2.96 [0.37, 23.81]	0.94
600-B-367-EU (150 mg)	7	75	1	41	3.59 [0.46, 28.19]	0.93
Claghorn 1990	28	51	11	69	2.58 [1.38, 4.81]	7.71
Cunningham 1994	31	41	9	67	3.64 [1.86, 7.09]	6.18
Cunningham 1997 (IR)	23	73	4	46	2.99 [1.10, 8.18]	3.71
Cunningham 1997 (XR)	20	77	5	45	2.06 [0.82, 5.17]	4.65
EudraCT 2004-000562-13	12	115	3	117	3.78 [1.09, 13.06]	2.18
Guelfi 1995	1	45	0	47	3.06 [0.13, 73.33]	0.35
Hewett 2010	9	189	9	178	0.94 [0.38, 2.33]	6.53
Higuchi 2016 (fixed dose)	21	153	7	85	1.59 [0.70, 3.59]	6.46
Higuchi 2016 (flexible dose)	31	149	8	83	1.96 [0.94, 4.09]	7.49
Khan 1998 (75 mg)	7	89	0	33	5.26 [0.31, 89.63]	0.52
Khan 1998 (150 mg)	4	92	0	33	3.15 [0.17, 57.08]	0.52
Khan 1998 (200 mg)	4	90	0	32	3.13 [0.17, 56.52]	0.52
Learned 2012	7	126	3	123	2.21 [0.58, 8.36]	2.17
Lieberman 2008 (225 mg)	22	95	13	112	1.81 [0.96, 3.42]	8.86
Mendels 1993 (25 mg)	15	64	3	23	1.65 [0.52, 5.24]	3.18
Mendels 1993 (50 - 75 mg)	13	63	3	23	1.48 [0.46, 4.79]	3.15
Mendels 1993 (150 - 200 mg)	22	57	3	23	2.41 [0.79, 7.41]	3.18
Rudolph 1998 (75 mg)	15	74	1	30	5.22 [0.72, 37.94]	1.05
Rudolph 1998 (225 mg)	16	73	1	30	5.57 [0.77, 40.30]	1.05
Rudolph 1998 (375 mg)	23	65	2	28	3.92 [0.98, 15.65]	2.10
Rudolph 1999	8	92	6	92	1.31 [0.47, 3.63]	4.27
Schatzberg 2006	8	94	3	93	2.51 [0.69, 9.18]	2.18
Schweizer 1994	19	54	11	67	1.85 [0.94, 3.61]	7.50
Silverstone 1999	17	111	7	112	2.26 [0.97, 5.25]	5.12
Thase 1997	26	69	11	91	2.54 [1.33, 4.85]	7.48
**Overall**					2.29 [1.89, 2.77]	

Heterogeneity: I^2 = 0.00%, H^2 = 1.00

Test of $\theta_i = \theta_j$: Q(26) = 13.35, p = 0.98

Test of θ = 0: z = 8.47, p = 0.00

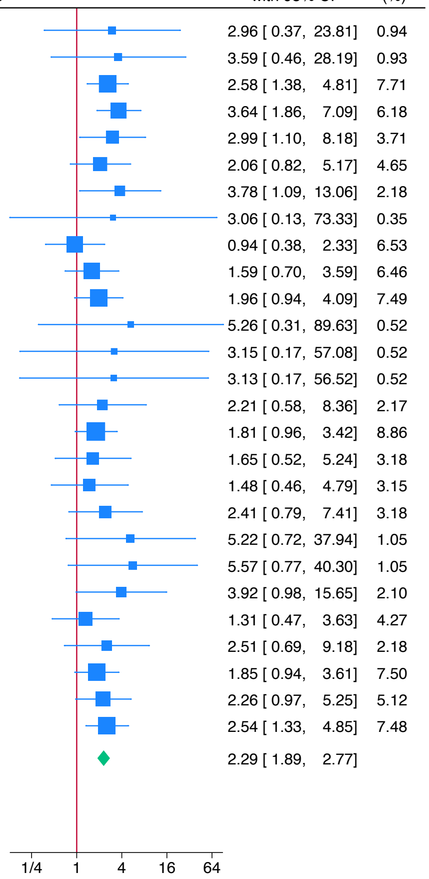

Fixed-effects Mantel–Haenszel model

Supplementary Figure 87: Meta-analysis of venlafaxine versus placebo on constipation (sensitivity analysis)

Study	Venlafaxine Events	Venlafaxine No events	Placebo Events	Placebo No events	Risk ratio with 95% CI	Weight (%)
600-B-367-EU (75 mg)	1	82	1	40	0.49 [0.03, 7.70]	1.25
600-B-367-EU (150 mg)	5	77	1	41	2.56 [0.31, 21.22]	1.24
Alvarez 2012	11	102	1	104	10.22 [1.34, 77.81]	0.97
Claghorn 1990	11	68	1	79	11.14 [1.47, 84.25]	0.93
Cunningham 1994	16	56	3	73	5.63 [1.71, 18.51]	2.73
Cunningham 1997 (IR)	14	82	2	48	3.65 [0.86, 15.41]	2.46
Cunningham 1997 (XR)	16	81	2	48	4.12 [0.99, 17.23]	2.47
EudraCT 2004-000562-13	6	121	5	115	1.13 [0.36, 3.62]	4.80
Guelfi 1995	2	44	1	46	2.04 [0.19, 21.77]	0.92
Hewett 2010	12	186	3	184	3.78 [1.08, 13.18]	2.88
Higuchi 2016 (fixed dose)	17	157	4	88	2.25 [0.78, 6.48]	4.89
Higuchi 2016 (flexible dose)	17	163	4	87	2.15 [0.74, 6.20]	4.96
Learned 2012	12	121	9	117	1.26 [0.55, 2.89]	8.64
Lieberman 2008 (225 mg)	9	108	2	123	4.81 [1.06, 21.79]	1.81
Mendels 1993 (25 mg)	6	73	2	24	0.99 [0.21, 4.60]	2.81
Mendels 1993 (50 - 75 mg)	9	67	2	24	1.54 [0.36, 6.67]	2.78
Mendels 1993 (150 - 200 mg)	15	64	2	24	2.47 [0.60, 10.08]	2.81
Nemeroff 2007	10	90	5	97	2.04 [0.72, 5.76]	4.63
Rudolph 1998 (75 mg)	17	72	3	28	1.97 [0.62, 6.28]	4.16
Rudolph 1998 (225 mg)	10	79	3	28	1.16 [0.34, 3.95]	4.16
Rudolph 1998 (375 mg)	17	71	4	26	1.45 [0.53, 3.97]	5.57
Schatzberg 2006	22	80	4	92	5.18 [1.85, 14.47]	3.85
Schweizer 1994	9	64	6	72	1.60 [0.60, 4.28]	5.42
Sheehan 2009	24	71	11	84	2.18 [1.13, 4.20]	10.28
Silverstone 1999	22	106	13	106	1.57 [0.83, 2.98]	12.59
**Overall**					2.33 [1.87, 2.91]	

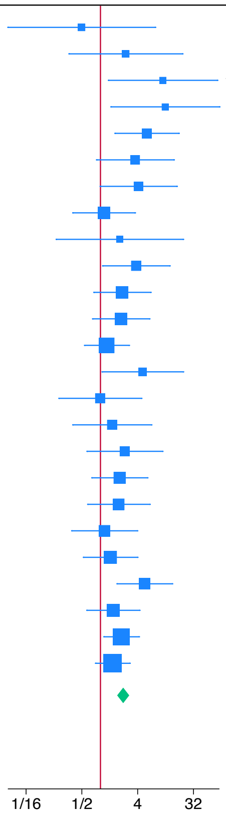

Heterogeneity: $I^2 = 0.00\%$, $H^2 = 1.00$

Test of $\theta_i = \theta_j$: $Q(24) = 21.87$, $p = 0.59$

Test of $\theta = 0$: $z = 7.48$, $p = 0.00$

Fixed-effects Mantel–Haenszel model

Supplementary Figure 88: Meta-analysis of venlafaxine versus placebo on nervousness (sensitivity analysis)

Study	Venlafaxine Events	Venlafaxine No events	Placebo Events	Placebo No events	Risk ratio with 95% CI	Weight (%)
600-B-367-EU (75 mg)	1	82	1	40	0.49 [0.03, 7.70]	2.57
600-B-367-EU (150 mg)	2	80	1	41	1.02 [0.10, 10.97]	2.54
Claghorn 1990	7	72	6	74	1.18 [0.42, 3.36]	11.47
Cunningham 1994	15	57	9	67	1.76 [0.82, 3.77]	16.84
Guelfi 1995	3	43	0	47	7.15 [0.38, 134.66]	0.95
Mendels 1993 (25 mg)	4	75	2	24	0.66 [0.13, 3.39]	5.79
Mendels 1993 (50 - 75 mg)	7	69	1	25	2.39 [0.31, 18.55]	2.87
Mendels 1993 (150 - 200 mg)	13	66	1	25	4.28 [0.59, 31.15]	2.89
Rudolph 1998 (75 mg)	19	70	1	30	6.62 [0.92, 47.40]	2.85
Rudolph 1998 (225 mg)	12	77	1	30	4.18 [0.57, 30.84]	2.85
Rudolph 1998 (375 mg)	11	77	2	28	1.88 [0.44, 7.98]	5.74
Rudolph 1999	12	88	5	93	2.35 [0.86, 6.43]	9.71
Schatzberg 2006	2	100	2	94	0.94 [0.14, 6.55]	3.96
Schweizer 1994	10	63	3	75	3.56 [1.02, 12.43]	5.58
Silverstone 1999	23	105	8	111	2.67 [1.24, 5.74]	15.95
Thase 1997	16	79	4	98	4.29 [1.49, 12.39]	7.42
**Overall**					2.39 [1.74, 3.30]	

Heterogeneity: $I^2 = 0.00\%$, $H^2 = 1.00$

Test of $\theta_i = \theta_j$: $Q(15) = 11.35$, $p = 0.73$

Test of $\theta = 0$: $z = 5.35$, $p = 0.00$

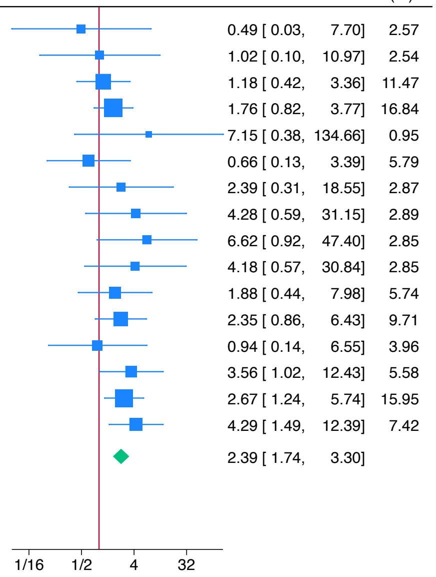

Fixed-effects Mantel–Haenszel model

Supplementary Figure 89: Meta-analysis of venlafaxine versus placebo on insomnia (sensitivity analysis)

Study	Venlafaxine Events	Venlafaxine No events	Placebo Events	Placebo No events	Risk ratio with 95% CI	Weight (%)
600-B-367-EU (75 mg)	2	81	2	39	0.49 [0.07, 3.38]	1.72
600-B-367-EU (150 mg)	3	79	1	41	1.54 [0.16, 14.32]	0.85
Alvarez 2012	14	99	5	100	2.60 [0.97, 6.97]	3.34
Claghorn 1990	13	66	6	74	2.19 [0.88, 5.48]	3.84
Cunningham 1994	7	65	3	73	2.46 [0.66, 9.16]	1.88
EudraCT 2004-000562-13	14	113	8	112	1.65 [0.72, 3.80]	5.30
Guelfi 1995	3	43	3	44	1.02 [0.22, 4.80]	1.91
Hewett 2009	7	180	4	193	1.84 [0.55, 6.20]	2.51
Hewett 2010	13	185	8	179	1.53 [0.65, 3.62]	5.30
Higuchi 2016 (fixed dose)	4	170	3	89	0.70 [0.16, 3.08]	2.53
Higuchi 2016 (flexible dose)	8	172	3	88	1.35 [0.37, 4.96]	2.57
Khan 1998 (75 mg)	5	91	0	33	3.86 [0.22, 67.91]	0.48
Khan 1998 (150 mg)	3	93	0	33	2.45 [0.13, 46.29]	0.48
Khan 1998 (200 mg)	5	89	0	32	3.82 [0.22, 67.25]	0.48
Learned 2012	7	126	11	115	0.60 [0.24, 1.51]	7.28
Mendels 1993 (25 mg)	14	65	4	22	1.15 [0.42, 3.19]	3.88
Mendels 1993 (50 - 75 mg)	10	66	4	22	0.86 [0.29, 2.49]	3.84
Mendels 1993 (150 - 200 mg)	19	60	3	23	2.08 [0.67, 6.48]	2.91
Nemeroff 2007	22	78	14	88	1.60 [0.87, 2.95]	8.93
Rudolph 1998 (75 mg)	20	69	3	28	2.32 [0.74, 7.28]	2.87
Rudolph 1998 (225 mg)	18	71	3	28	2.09 [0.66, 6.61]	2.87
Rudolph 1998 (375 mg)	12	76	3	27	1.36 [0.41, 4.51]	2.88
Schatzberg 2006	10	92	4	92	2.35 [0.76, 7.25]	2.65
Schweizer 1994	15	58	10	68	1.60 [0.77, 3.34]	6.23
Sheehan 2009	18	77	8	87	2.25 [1.03, 4.92]	5.15
Silverstone 1999	41	87	12	107	3.18 [1.76, 5.75]	8.01
Thase 1997	33	62	15	87	2.36 [1.37, 4.06]	9.32
**Overall**					1.82 [1.51, 2.19]	

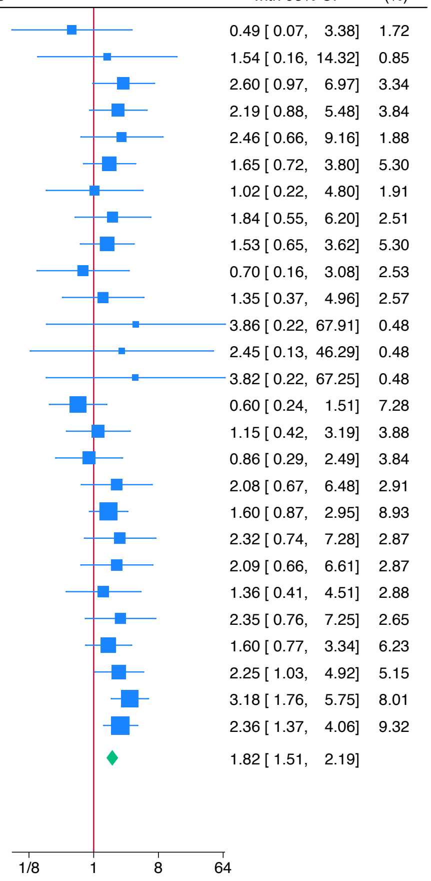

Heterogeneity: $I^2 = 0.00\%$, $H^2 = 1.00$

Test of $\theta_i = \theta_j$: $Q(26) = 19.57$, $p = 0.81$

Test of $\theta = 0$: $z = 6.29$, $p = 0.00$

Fixed-effects Mantel–Haenszel model

Supplementary Figure 90: Meta-analysis of venlafaxine versus placebo on asthenia (sensitivity analysis)

Study	Venlafaxine Events	Venlafaxine No events	Placebo Events	Placebo No events	Risk ratio with 95% CI	Weight (%)
600-B-367-EU (75 mg)	5	78	1	40	2.47 [0.30, 20.46]	1.62
600-B-367-EU (150 mg)	3	79	1	41	1.54 [0.16, 14.32]	1.60
Alvarez 2012	11	102	6	99	1.70 [0.65, 4.44]	7.54
Claghorn 1990	5	74	2	78	2.53 [0.51, 12.67]	2.41
Cunningham 1994	7	65	3	73	2.46 [0.66, 9.16]	3.54
EudraCT 2004-000562-13	10	117	2	118	4.72 [1.06, 21.12]	2.49
Guelfi 1995	2	44	0	47	5.11 [0.25, 103.55]	0.60
Hewett 2009	9	178	4	193	2.37 [0.74, 7.57]	4.72
Hewett 2010	16	182	13	174	1.16 [0.57, 2.35]	16.21
Khan 1998 (75 mg)	5	91	0	33	3.86 [0.22, 67.91]	0.90
Khan 1998 (150 mg)	5	91	0	33	3.86 [0.22, 67.91]	0.90
Khan 1998 (200 mg)	3	91	0	32	2.43 [0.13, 45.84]	0.90
Learned 2012	13	120	5	121	2.46 [0.90, 6.71]	6.23
Lieberman 2008 (225 mg)	7	110	8	117	0.93 [0.35, 2.50]	9.38
Mendels 1993 (25 mg)	10	69	3	23	1.10 [0.33, 3.69]	5.47
Mendels 1993 (50 - 75 mg)	8	68	3	23	0.91 [0.26, 3.18]	5.42
Mendels 1993 (150 - 200 mg)	13	66	2	24	2.14 [0.52, 8.86]	3.65
Nemeroff 2007	10	90	5	97	2.04 [0.72, 5.76]	6.00
Rudolph 1999	10	90	2	96	4.90 [1.10, 21.79]	2.45
Schatzberg 2006	12	90	5	91	2.26 [0.83, 6.17]	6.25
Schweizer 1994	9	64	10	68	0.96 [0.41, 2.23]	11.72
**Overall**					1.81 [1.39, 2.35]	

Heterogeneity: I^2 = 0.00%, H^2 = 1.00

Test of $\theta_i = \theta_j$: Q(20) = 12.89, p = 0.88

Test of θ = 0: z = 4.40, p = 0.00

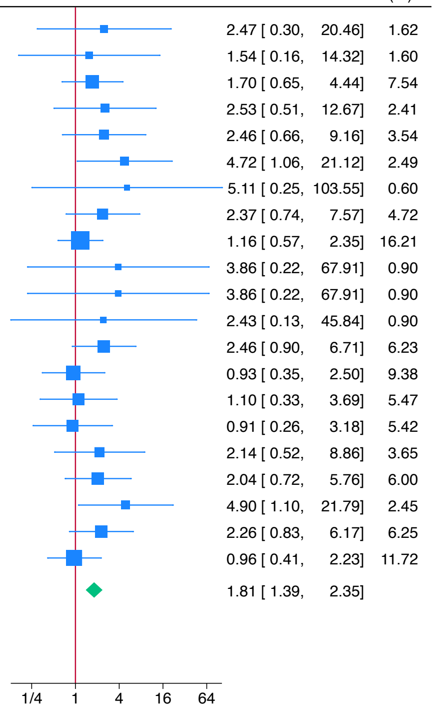

Fixed-effects Mantel–Haenszel model

Supplementary Figure 91: Meta-analysis of venlafaxine versus placebo on tremor (sensitivity analysis)

Study	Venlafaxine Events	Venlafaxine No events	Placebo Events	Placebo No events	Risk ratio with 95% CI	Weight (%)
600-B-367-EU (75 mg)	3	80	2	39	0.74 [0.13, 4.26]	10.28
600-B-367-EU (150 mg)	2	80	1	41	1.02 [0.10, 10.97]	5.08
Alvarez 2012	6	107	3	102	1.86 [0.48, 7.24]	11.94
Claghorn 1990	2	77	0	80	5.06 [0.25, 103.80]	1.91
EudraCT 2004-000562-13	4	123	3	117	1.26 [0.29, 5.51]	11.84
Guelfi 1995	2	45	2	45	1.00 [0.15, 6.81]	7.68
Hewett 2010	10	188	2	185	4.72 [1.05, 21.27]	7.90
Learned 2012	7	126	5	121	1.33 [0.43, 4.07]	19.71
Rudolph 1999	9	91	1	97	8.82 [1.14, 68.32]	3.88
Schatzberg 2006	6	96	0	96	12.24 [0.70, 214.43]	1.98
Sheehan 2009	5	90	0	95	11.00 [0.62, 196.19]	1.92
Silverstone 1999	13	115	4	115	3.02 [1.01, 9.01]	15.91
**Overall**					2.58 [1.65, 4.04]	

Heterogeneity: $I^2 = 0.00\%$, $H^2 = 1.00$

Test of $\theta_i = \theta_j$: $Q(11) = 10.35$, $p = 0.50$

Test of $\theta = 0$: $z = 4.16$, $p = 0.00$

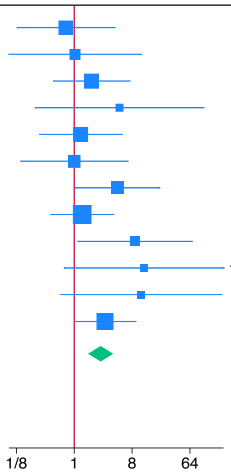

Fixed-effects Mantel–Haenszel model

Supplementary Figure 92: Meta-analysis of venlafaxine versus placebo on decreased appetite (sensitivity analysis)

Study	Venlafaxine Events	Venlafaxine No events	Placebo Events	Placebo No events	Risk ratio with 95% CI	Weight (%)
Higuchi 2016 (fixed dose)	4	170	0	91	4.73 [0.26, 86.93]	9.20
Higuchi 2016 (flexible dose)	4	176	1	91	2.04 [0.23, 18.03]	18.57
Learned 2012	2	131	1	125	1.89 [0.17, 20.64]	14.41
Schatzberg 2006	11	91	4	92	2.59 [0.85, 7.85]	57.82
**Overall**					2.58 [1.08, 6.18]	

Heterogeneity: I^2 = 0.00%, H^2 = 1.00

Test of $\theta_i = \theta_j$: Q(3) = 0.28, p = 0.96

Test of θ = 0: z = 2.14, p = 0.03

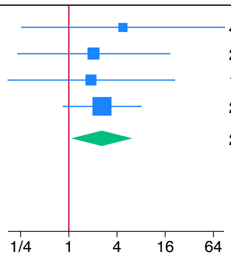

Fixed-effects Mantel–Haenszel model

Supplementary Figure 93: Meta-analysis of venlafaxine versus placebo on abdominal pain (sensitivity analysis)

Study	Venlafaxine Events	Venlafaxine No events	Placebo Events	Placebo No events	Risk ratio with 95% CI	Weight (%)
600-B-367-EU (75 mg)	0	83	2	39	0.10 [0.00, 2.04]	9.78
600-B-367-EU (150 mg)	2	80	1	41	1.02 [0.10, 10.97]	3.88
EudraCT 2004-000562-13	8	119	11	109	0.69 [0.29, 1.65]	33.19
Hewett 2010	9	189	8	179	1.06 [0.42, 2.70]	24.14
Higuchi 2016 (fixed dose)	5	169	3	88	0.87 [0.21, 3.57]	11.56
Higuchi 2016 (flexible dose)	0	180	4	88	0.06 [0.00, 1.05]	17.44
**Overall**					0.64 [0.39, 1.07]	

Heterogeneity: $I^2 = 10.41\%$, $H^2 = 1.12$

Test of $\theta_i = \theta_j$: Q(5) = 5.58, p = 0.35

Test of $\theta = 0$: z = -1.69, p = 0.09

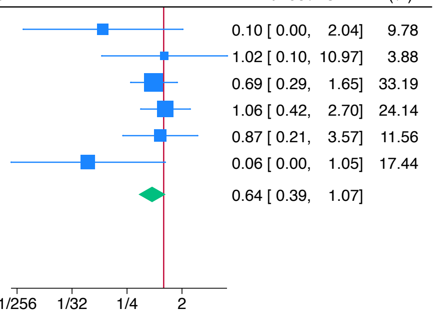

Fixed-effects Mantel–Haenszel model

Supplementary Figure 94: Meta-analysis of venlafaxine versus placebo on abnormal dreams (sensitivity analysis)

Study	Venlafaxine Events	Venlafaxine No events	Placebo Events	Placebo No events	Risk ratio with 95% CI	Weight (%)
600-B-367-EU (75 mg)	2	81	1	40	0.99 [0.09, 10.58]	40.44
600-B-367-EU (150 mg)	0	82	0	42	0.52 [0.01, 25.66]	19.90
Cunningham 1997 (IR)	7	89	0	50	7.89 [0.46, 135.33]	19.80
Cunningham 1997 (XR)	12	85	0	50	13.01 [0.79, 215.32]	19.87
**Overall**					4.65 [1.28, 16.93]	

Heterogeneity: $I^2 = 14.35\%$, $H^2 = 1.17$

Test of $\theta_i = \theta_j$: Q(3) = 3.50, p = 0.32

Test of $\theta = 0$: z = 2.33, p = 0.02

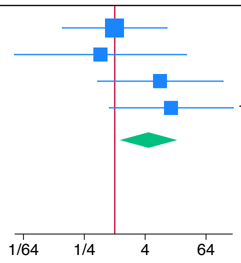

Fixed-effects Mantel–Haenszel model

Supplementary Figure 95: Meta-analysis of venlafaxine versus placebo on abnormal vision (sensitivity analysis)

Study	Venlafaxine Events	Venlafaxine No events	Placebo Events	Placebo No events	Risk ratio with 95% CI	Weight (%)
600-B-367-EU (75 mg)	0	83	1	40	0.17 [0.01, 4.00]	11.91
600-B-367-EU (150 mg)	0	82	0	42	0.52 [0.01, 25.66]	3.92
Alvarez 2012	6	107	2	103	2.79 [0.58, 13.51]	12.35
EudraCT 2004-000562-13	3	124	0	120	6.62 [0.35, 126.78]	3.06
Guelfi 1995	1	45	0	47	3.06 [0.13, 73.33]	2.95
Lieberman 2008 (225 mg)	5	112	3	122	1.78 [0.44, 7.29]	17.27
Schatzberg 2006	8	94	5	91	1.51 [0.51, 4.44]	30.68
Sheehan 2009	10	85	3	92	3.33 [0.95, 11.73]	17.86
**Overall**					2.04 [1.16, 3.61]	

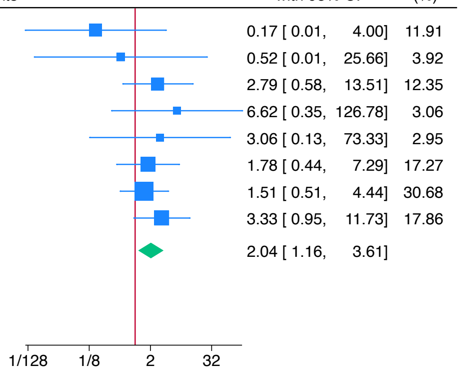

Heterogeneity: $I^2 = 0.00\%$, $H^2 = 1.00$

Test of $\theta_i = \theta_j$: Q(7) = 4.61, p = 0.71

Test of $\theta = 0$: z = 2.46, p = 0.01

Fixed-effects Mantel–Haenszel model

Supplementary Figure 96: Meta-analysis of venlafaxine versus placebo on agitation (sensitivity analysis)

Study	Venlafaxine Events	Venlafaxine No events	Placebo Events	Placebo No events	Risk ratio with 95% CI	Weight (%)
600-B-367-EU (75 mg)	2	81	0	41	2.50 [0.12, 50.91]	9.62
600-B-367-EU (150 mg)	2	80	0	42	2.59 [0.13, 52.76]	9.51
Cunningham 1994	7	65	0	76	15.82 [0.92, 272.09]	7.02
Guelfi 1995	1	45	0	47	3.06 [0.13, 73.33]	7.14
Learned 2012	1	132	4	122	0.24 [0.03, 2.09]	59.28
Schatzberg 2006	2	100	0	96	4.71 [0.23, 96.84]	7.43
**Overall**					2.31 [0.94, 5.64]	

Heterogeneity: $I^2 = 19.48\%$, $H^2 = 1.24$

Test of $\theta_i = \theta_j$: Q(5) = 6.21, p = 0.29

Test of $\theta = 0$: z = 1.83, p = 0.07

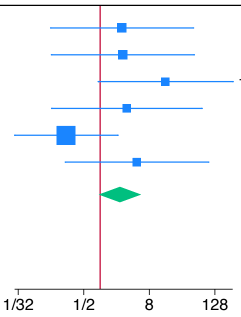

Fixed-effects Mantel–Haenszel model

Supplementary Figure 97: Meta-analysis of venlafaxine versus placebo on back pain (sensitivity analysis)

Study	Venlafaxine Events	Venlafaxine No events	Placebo Events	Placebo No events	Risk ratio with 95% CI	Weight (%)
600-B-367-EU (75 mg)	0	83	1	40	0.17 [0.01, 4.00]	12.76
600-B-367-EU (150 mg)	1	81	1	41	0.51 [0.03, 7.99]	8.44
EudraCT 2004-000562-13	3	124	6	114	0.47 [0.12, 1.85]	39.36
Schatzberg 2006	3	99	6	90	0.47 [0.12, 1.83]	39.44
**Overall**					0.44 [0.18, 1.04]	

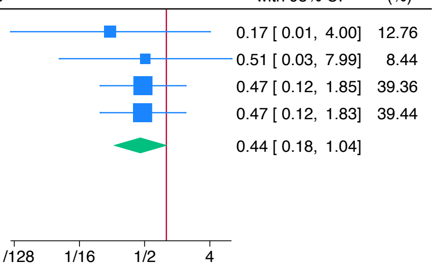

Heterogeneity: $I^2 = 0.00\%$, $H^2 = 1.00$

Test of $\theta_i = \theta_j$: Q(3) = 0.39, p = 0.94

Test of $\theta = 0$: z = -1.88, p = 0.06

Fixed-effects Mantel–Haenszel model

Supplementary Figure 98: Meta-analysis of venlafaxine versus placebo on increased blood pressure (sensitivity analysis)

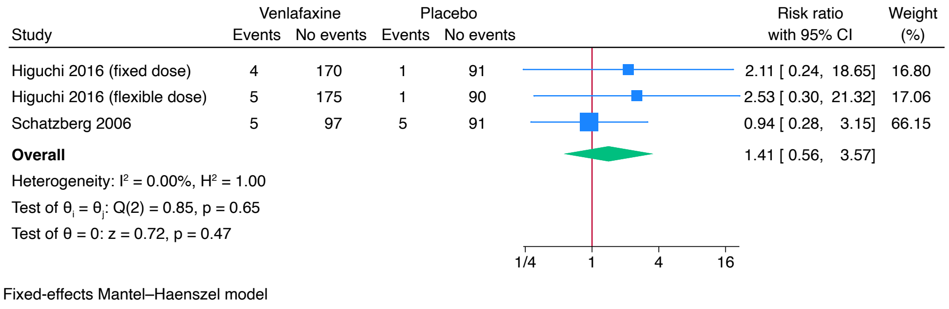

Supplementary Figure 99: Meta-analysis of venlafaxine versus placebo on coughing (sensitivity analysis)

Study	Venlafaxine Events	Venlafaxine No events	Placebo Events	Placebo No events	Risk ratio with 95% CI	Weight (%)
600-B-367-EU (75 mg)	1	82	0	41	1.50 [0.06, 36.04]	10.91
600-B-367-EU (150 mg)	1	81	1	41	0.51 [0.03, 7.99]	21.64
Schatzberg 2006	2	100	4	92	0.47 [0.09, 2.51]	67.45
**Overall**					0.59 [0.17, 2.12]	

Heterogeneity: $I^2 = 0.00\%$, $H^2 = 1.00$

Test of $\theta_i = \theta_j$: Q(2) = 0.41, p = 0.81

Test of $\theta = 0$: z = -0.81, p = 0.42

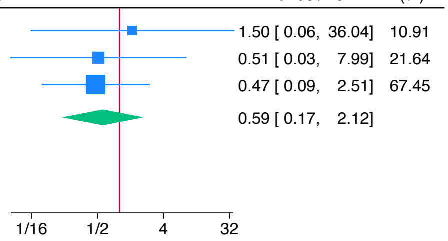

Fixed-effects Mantel–Haenszel model

Supplementary Figure 100: Meta-analysis of venlafaxine versus placebo on diarrhoea (sensitivity analysis)

Study	Venlafaxine Events	Venlafaxine No events	Placebo Events	Placebo No events	Risk ratio with 95% CI	Weight (%)
600-B-367-EU (75 mg)	3	80	3	38	0.49 [0.10, 2.34]	3.73
600-B-367-EU (150 mg)	1	81	3	39	0.17 [0.02, 1.59]	2.02
Alvarez 2012	5	108	5	100	0.93 [0.28, 3.12]	5.46
Cunningham 1994	7	65	5	71	1.48 [0.49, 4.45]	6.22
Cunningham 1997 (IR)	5	91	3	47	0.87 [0.22, 3.48]	4.45
Cunningham 1997 (XR)	13	84	3	47	2.23 [0.67, 7.48]	5.47
EudraCT 2004-000562-13	3	124	6	114	0.47 [0.12, 1.85]	4.59
EudraCT 2007-007025-51	0	7	2	5	0.20 [0.01, 3.54]	1.27
Guelfi 1995	0	46	1	46	0.34 [0.01, 8.15]	1.05
Hewett 2010	10	188	9	178	1.05 [0.44, 2.53]	8.23
Higuchi 2016 (fixed dose)	6	168	3	88	1.05 [0.27, 4.09]	4.59
Higuchi 2016 (flexible dose)	8	172	4	88	1.02 [0.32, 3.31]	5.70
Learned 2012	8	125	8	118	0.95 [0.37, 2.45]	7.52
Nemeroff 2007	9	91	9	93	1.02 [0.42, 2.46]	8.19
Rudolph 1999	14	86	9	89	1.52 [0.69, 3.36]	9.23
Schatzberg 2006	12	90	13	83	0.87 [0.42, 1.81]	9.92
Silverstone 1999	22	106	19	100	1.08 [0.61, 1.89]	12.36
**Overall**					0.97 [0.69, 1.36]	

Heterogeneity: $\tau^2 = 0.16$, $I^2 = 34.54\%$, $H^2 = 1.53$

Test of $\theta_i = \theta_j$: $Q(16) = 9.57$, $p = 0.89$

Test of $\theta = 0$: $z = -0.18$, $p = 0.85$

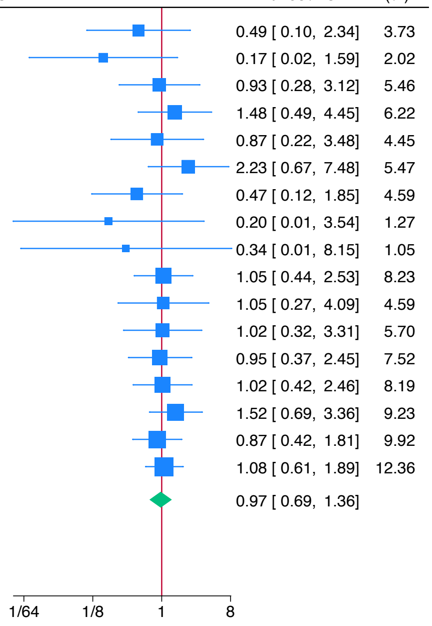

Random-effects Sidik–Jonkman model

Supplementary Figure 101: Meta-analysis of venlafaxine versus placebo on dyspepsia (sensitivity analysis)

Study	Venlafaxine Events	Venlafaxine No events	Placebo Events	Placebo No events	Risk ratio with 95% CI	Weight (%)
600-B-367-EU (75 mg)	2	81	2	39	0.49 [0.07, 3.38]	5.06
600-B-367-EU (150 mg)	1	81	2	40	0.26 [0.02, 2.74]	5.00
Cunningham 1994	2	70	5	71	0.42 [0.08, 2.11]	9.19
Learned 2012	2	131	2	124	0.95 [0.14, 6.62]	3.88
Nemeroff 2007	9	91	16	86	0.57 [0.27, 1.24]	29.94
Schatzberg 2006	11	91	8	88	1.29 [0.54, 3.08]	15.58
Silverstone 1999	13	115	16	103	0.76 [0.38, 1.50]	31.34
**Overall**					0.72 [0.49, 1.07]	

Heterogeneity: I^2 = 0.00%, H^2 = 1.00

Test of $\theta_i = \theta_j$: Q(6) = 3.49, p = 0.75

Test of θ = 0: z = -1.61, p = 0.11

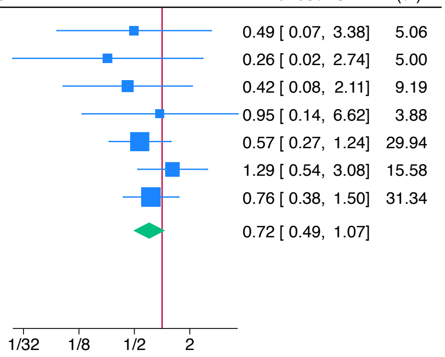

Fixed-effects Mantel–Haenszel model

Supplementary Figure 102: Meta-analysis of venlafaxine versus placebo on flatulence (sensitivity analysis)

Study	Venlafaxine Events	Venlafaxine No events	Placebo Events	Placebo No events	Risk ratio with 95% CI	Weight (%)
600-B-367-EU (75 mg)	0	83	1	40	0.17 [0.01, 4.00]	63.32
600-B-367-EU (150 mg)	0	82	0	42	0.52 [0.01, 25.66]	20.85
EudraCT 2007-007025-51	0	7	0	7	1.00 [0.02, 44.50]	15.83
**Overall**					0.37 [0.05, 2.55]	

Heterogeneity: $I^2 = 0.00\%$, $H^2 = 1.00$

Test of $\theta_i = \theta_j$: $Q(2) = 0.53$, $p = 0.77$

Test of $\theta = 0$: $z = -1.01$, $p = 0.31$

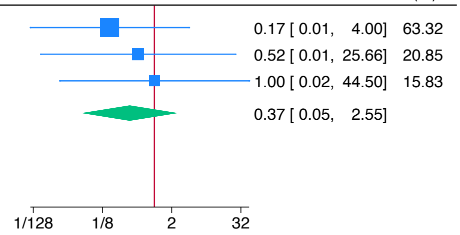

Fixed-effects Mantel–Haenszel model

Supplementary Figure 103: Meta-analysis of venlafaxine versus placebo on headache (sensitivity analysis)

Study	Venlafaxine Events	Venlafaxine No events	Placebo Events	Placebo No events	Risk ratio with 95% CI	Weight (%)
600-B-367-EU (75 mg)	11	72	5	36	1.09 [0.40, 2.92]	1.60
600-B-367-EU (150 mg)	7	75	6	36	0.60 [0.21, 1.67]	1.90
Alvarez 2012	32	81	26	79	1.14 [0.73, 1.78]	6.45
Claghorn 1990	21	58	17	63	1.25 [0.72, 2.19]	4.04
Cunningham 1994	22	50	25	51	0.93 [0.58, 1.49]	5.82
EudraCT 2004-000562-13	29	98	34	86	0.81 [0.53, 1.24]	8.37
EudraCT 2007-007025-51	0	7	1	6	0.33 [0.02, 7.02]	0.36
Guelfi 1995	2	44	4	43	0.51 [0.10, 2.65]	0.95
Hewett 2009	25	162	19	178	1.39 [0.79, 2.43]	4.43
Hewett 2010	28	170	31	156	0.85 [0.53, 1.37]	7.63
Higuchi 2016 (fixed dose)	16	158	7	85	1.21 [0.52, 2.83]	2.19
Higuchi 2016 (flexible dose)	18	162	7	84	1.30 [0.56, 3.00]	2.23
Learned 2012	28	105	24	102	1.11 [0.68, 1.80]	5.90
Mendels 1993 (25 mg)	20	59	7	19	0.94 [0.45, 1.97]	2.52
Mendels 1993 (50 - 75 mg)	19	57	7	19	0.93 [0.44, 1.95]	2.50
Mendels 1993 (150 - 200 mg)	17	62	7	19	0.80 [0.37, 1.71]	2.52
Nemeroff 2007	36	64	34	68	1.08 [0.74, 1.58]	8.06
Rudolph 1998 (75 mg)	23	66	7	24	1.14 [0.55, 2.40]	2.49
Rudolph 1998 (225 mg)	23	66	7	24	1.14 [0.55, 2.40]	2.49
Rudolph 1998 (375 mg)	22	66	8	22	0.94 [0.47, 1.88]	2.86
Schatzberg 2006	27	75	21	75	1.21 [0.74, 1.99]	5.18
Schweizer 1994	19	54	21	57	0.97 [0.57, 1.65]	4.86
Silverstone 1999	57	71	59	60	0.90 [0.69, 1.17]	14.64
**Overall**					1.01 [0.90, 1.13]	

Heterogeneity: $I^2 = 0.00\%$, $H^2 = 1.00$

Test of $\theta_i = \theta_j$: Q(22) = 8.71, p = 0.99

Test of $\theta = 0$: z = 0.12, p = 0.91

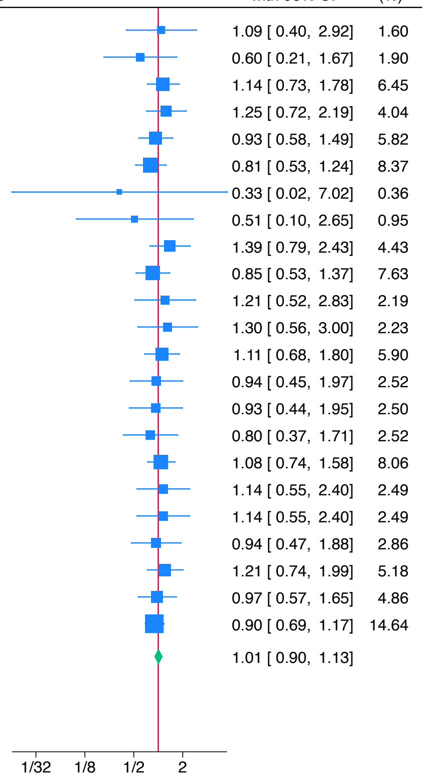

Fixed-effects Mantel–Haenszel model

Supplementary Figure 104: Meta-analysis of venlafaxine versus placebo on infection (sensitivity analysis)

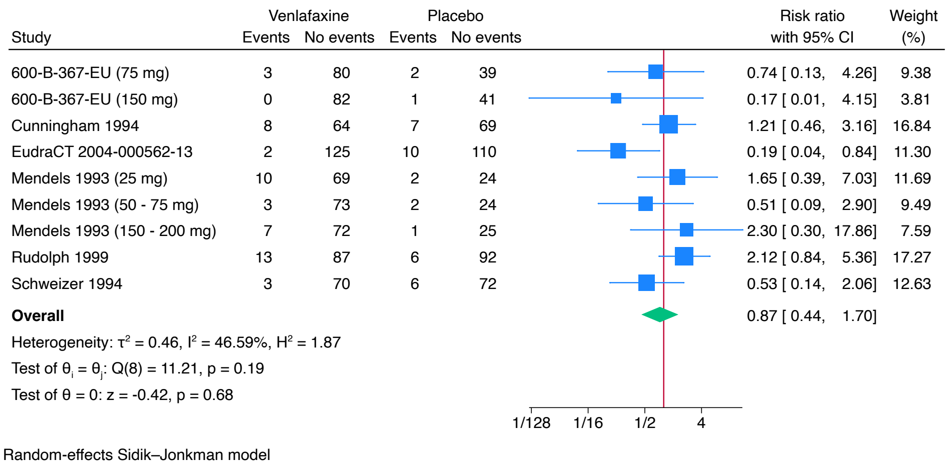

Supplementary Figure 105: Meta-analysis of venlafaxine versus placebo on influenza (sensitivity analysis)

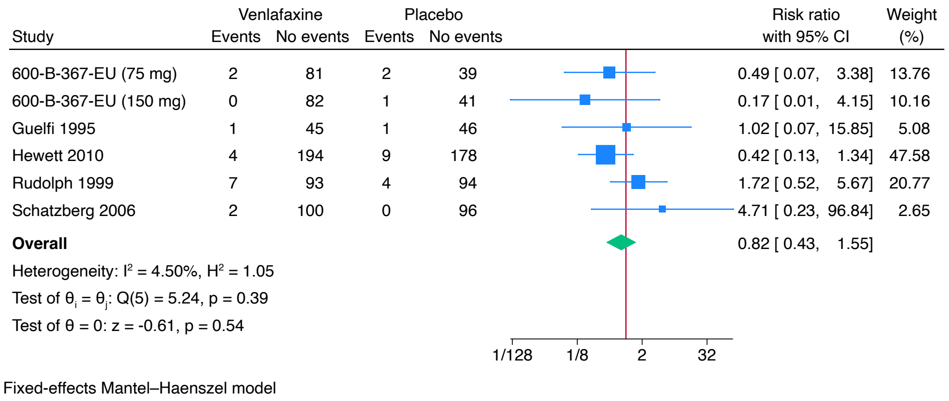

Supplementary Figure 106: Meta-analysis of venlafaxine versus placebo on malaise (sensitivity analysis)

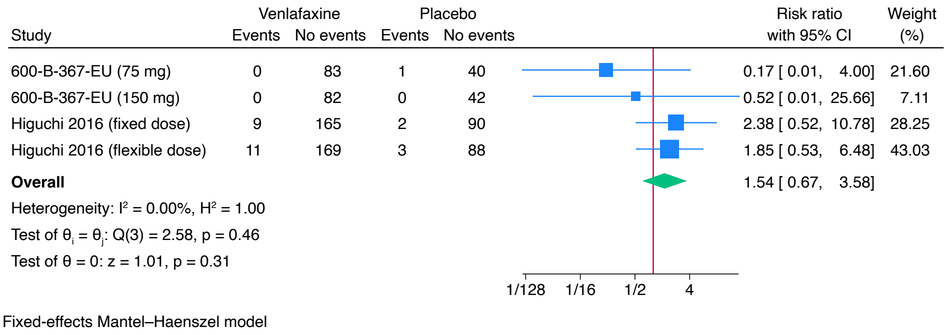

Supplementary Figure 107: Meta-analysis of venlafaxine versus placebo on nasopharyngitis (sensitivity analysis)

Study	Venlafaxine Events	Venlafaxine No events	Placebo Events	Placebo No events	Risk ratio with 95% CI	Weight (%)
Alvarez 2012	4	109	9	96	0.41 [0.13, 1.30]	11.69
Hewett 2010	11	187	9	178	1.15 [0.49, 2.72]	11.59
Higuchi 2016 (fixed dose)	35	139	20	72	0.93 [0.57, 1.51]	32.77
Higuchi 2016 (flexible dose)	32	148	21	70	0.77 [0.47, 1.26]	34.94
Learned 2012	6	127	7	119	0.81 [0.28, 2.35]	9.00
**Overall**					0.83 [0.62, 1.11]	

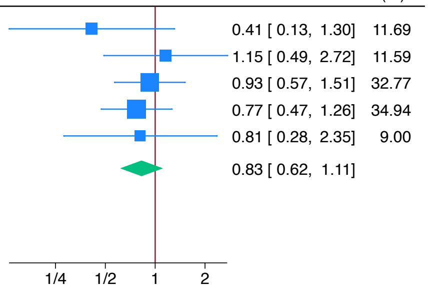

Heterogeneity: I^2 = 0.00%, H^2 = 1.00

Test of $\theta_i = \theta_j$: Q(4) = 2.27, p = 0.69

Test of θ = 0: z = -1.25, p = 0.21

Fixed-effects Mantel–Haenszel model

Supplementary Figure 108: Meta-analysis of venlafaxine versus placebo on palpitations (sensitivity analysis)

Study	Venlafaxine Events	Venlafaxine No events	Placebo Events	Placebo No events	Risk ratio with 95% CI	Weight (%)
600-B-367-EU (75 mg)	0	83	1	40	0.17 [0.01, 4.00]	15.12
600-B-367-EU (150 mg)	3	79	1	41	1.54 [0.16, 14.32]	10.00
Higuchi 2016 (fixed dose)	8	166	2	89	2.09 [0.45, 9.65]	19.86
Higuchi 2016 (flexible dose)	6	174	3	89	1.02 [0.26, 3.99]	30.02
Learned 2012	5	128	2	124	2.37 [0.47, 11.99]	15.53
Rudolph 1998 (75 mg)	1	22	0	13	1.75 [0.08, 40.11]	4.77
Rudolph 1998 (225 mg)	2	20	0	13	3.04 [0.16, 58.90]	4.70
**Overall**					1.50 [0.74, 3.00]	

Heterogeneity: $I^2 = 0.00\%$, $H^2 = 1.00$

Test of $\theta_i = \theta_j$: $Q(6) = 2.85$, $p = 0.83$

Test of $\theta = 0$: $z = 1.13$, $p = 0.26$

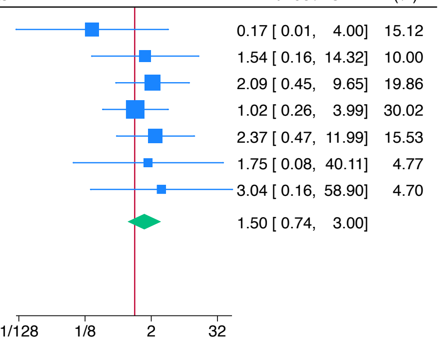

Fixed-effects Mantel–Haenszel model

Supplementary Figure 109: Meta-analysis of venlafaxine versus placebo on rhinitis (sensitivity analysis)

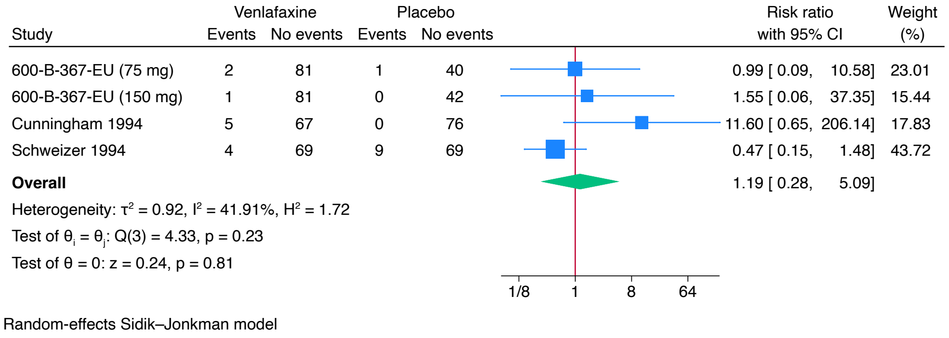

Supplementary Figure 110: Meta-analysis of venlafaxine versus placebo on tachycardia (sensitivity analysis)

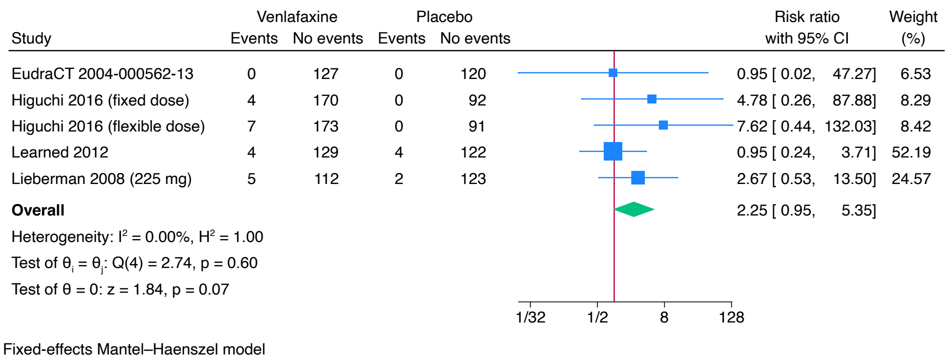

Supplementary Figure 111: Meta-analysis of venlafaxine versus placebo on urinary frequency (sensitivity analysis)

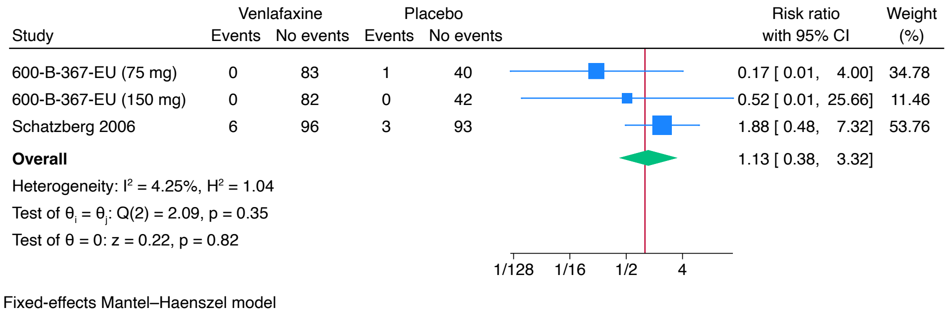

Supplementary Figure 112: Meta-analysis of venlafaxine versus placebo on vertigo (sensitivity analysis)

Study	Venlafaxine Events	Venlafaxine No events	Placebo Events	Placebo No events	Risk ratio with 95% CI	Weight (%)
600-B-367-EU (75 mg)	2	81	1	40	0.99 [0.09, 10.58]	11.23
600-B-367-EU (150 mg)	2	80	1	41	1.02 [0.10, 10.97]	11.09
EudraCT 2004-000562-13	9	118	5	115	1.70 [0.59, 4.93]	43.12
Guelfi 1995	0	46	1	46	0.34 [0.01, 8.15]	12.45
Higuchi 2016 (fixed dose)	4	170	1	90	2.09 [0.24, 18.44]	11.01
Higuchi 2016 (flexible dose)	6	174	1	91	3.07 [0.37, 25.09]	11.10
**Overall**					1.57 [0.76, 3.26]	

Heterogeneity: $I^2 = 0.00\%$, $H^2 = 1.00$

Test of $\theta_i = \theta_j$: Q(5) = 1.64, p = 0.90

Test of $\theta = 0$: z = 1.21, p = 0.23

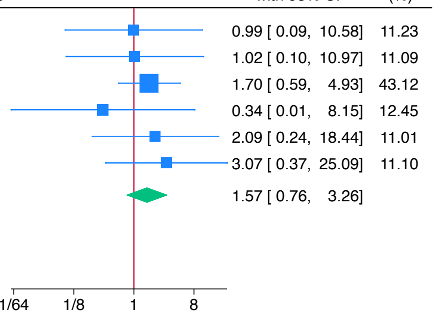

Fixed-effects Mantel–Haenszel model

Supplementary Figure 113: Meta-analysis of venlafaxine versus placebo on vomiting (sensitivity analysis)

Study	Venlafaxine Events	Venlafaxine No events	Placebo Events	Placebo No events	Risk ratio with 95% CI	Weight (%)
600-B-367-EU (75 mg)	2	81	1	40	0.99 [0.09, 10.58]	5.15
600-B-367-EU (150 mg)	3	79	1	41	1.54 [0.16, 14.32]	5.09
Alvarez 2012	4	109	1	104	3.72 [0.42, 32.72]	3.99
EudraCT 2004-000562-13	3	124	2	118	1.42 [0.24, 8.34]	7.91
Guelfi 1995	0	46	2	45	0.20 [0.01, 4.14]	9.52
Higuchi 2016 (fixed dose)	5	169	3	89	0.88 [0.22, 3.61]	15.10
Higuchi 2016 (flexible dose)	4	176	4	87	0.51 [0.13, 1.98]	20.44
Lieberman 2008 (225 mg)	3	114	0	125	7.47 [0.39, 143.17]	1.86
Nemeroff 2007	11	89	2	100	5.61 [1.28, 24.67]	7.62
Schatzberg 2006	9	93	2	94	4.24 [0.94, 19.11]	7.93
Sheehan 2009	9	86	4	91	2.25 [0.72, 7.06]	15.39
**Overall**					1.89 [1.19, 3.02]	

Heterogeneity: $I^2 = 14.66\%$, $H^2 = 1.17$

Test of $\theta_i = \theta_j$: Q(10) = 11.72, p = 0.30

Test of $\theta = 0$: z = 2.69, p = 0.01

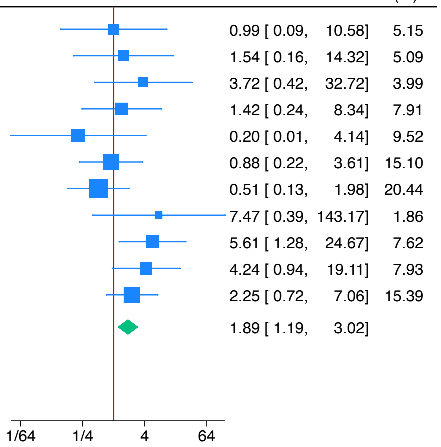

Fixed-effects Mantel–Haenszel model

Supplementary Figure 114: Meta-analysis of venlafaxine versus placebo on weight loss (sensitivity analysis)

Study	Venlafaxine Events	Venlafaxine No events	Placebo Events	Placebo No events	Risk ratio with 95% CI	Weight (%)
600-B-367-EU (75 mg)	2	81	0	41	2.50 [0.12, 50.91]	28.55
600-B-367-EU (150 mg)	1	81	0	42	1.55 [0.06, 37.35]	28.21
Guelfi 1995	2	44	0	47	5.11 [0.25, 103.55]	21.19
Schatzberg 2006	1	101	0	96	2.83 [0.12, 68.52]	22.05
**Overall**					2.86 [0.63, 13.02]	

Heterogeneity: $I^2 = 0.00\%$, $H^2 = 1.00$

Test of $\theta_i = \theta_j$: Q(3) = 0.29, p = 0.96

Test of $\theta = 0$: z = 1.36, p = 0.17

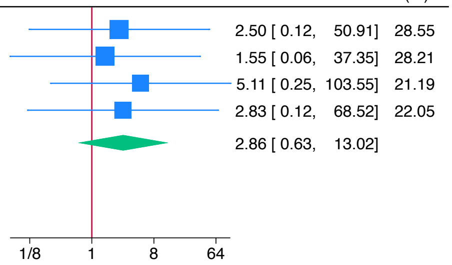

Fixed-effects Mantel–Haenszel model

Supplementary Figure 115: Meta-analysis of venlafaxine versus placebo on abnormality of accommodation (sensitivity analysis)

Study	Venlafaxine Events	Venlafaxine No events	Placebo Events	Placebo No events	Risk ratio with 95% CI	Weight (%)
600-B-367-EU (75 mg)	0	83	0	41	0.50 [0.01, 24.76]	14.07
600-B-367-EU (150 mg)	2	80	0	42	2.59 [0.13, 52.76]	13.90
Cunningham 1994	7	65	3	73	2.46 [0.66, 9.16]	61.59
Guelfi 1995	2	44	0	47	5.11 [0.25, 103.55]	10.44
**Overall**					2.48 [0.87, 7.07]	

Heterogeneity: $I^2 = 0.00\%$, $H^2 = 1.00$

Test of $\theta_i = \theta_j$: Q(3) = 0.87, p = 0.83

Test of $\theta = 0$: z = 1.70, p = 0.09

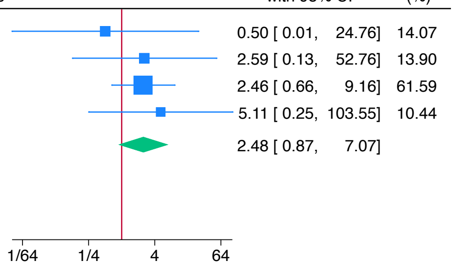

Fixed-effects Mantel–Haenszel model

Supplementary Figure 116: Meta-analysis of venlafaxine versus placebo on pruritis (sensitivity analysis)

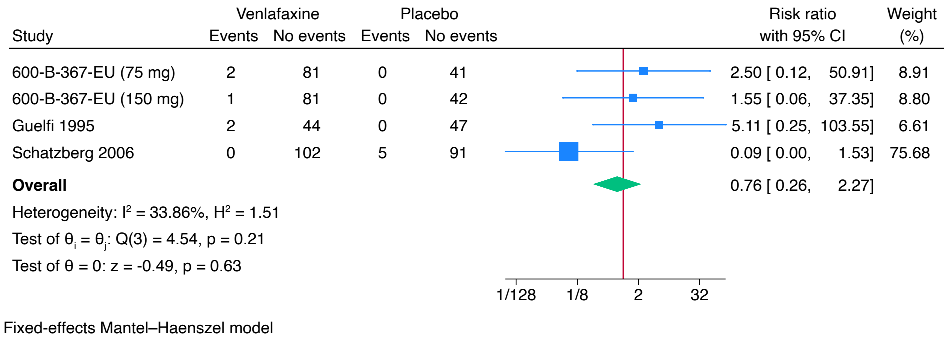

Supplementary Figure 117: Meta-analysis of venlafaxine versus placebo on vasodilation (sensitivity analysis)

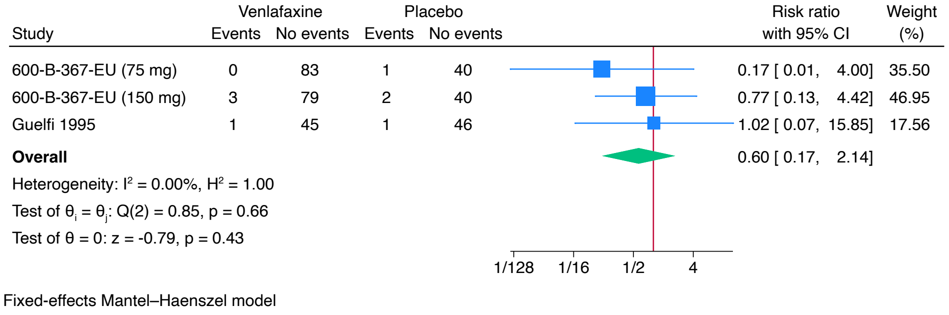

STATA®

Supplementary Figure 118: Meta-analysis of venlafaxine versus placebo on neck pain (sensitivity analysis)

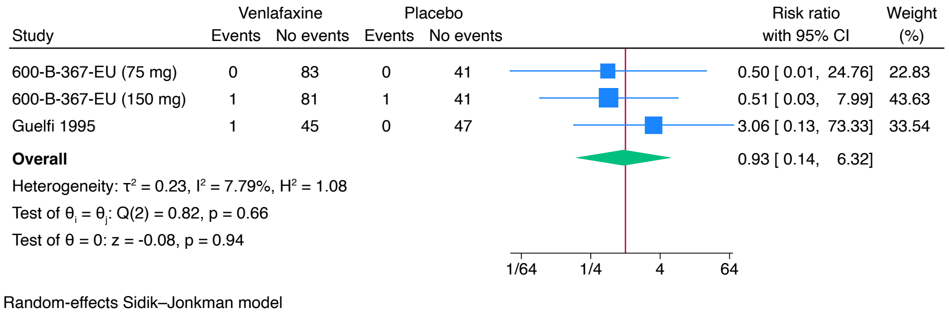

Supplementary Figure 119: Meta-analysis of venlafaxine versus placebo on pain (sensitivity analysis)

Study	Venlafaxine Events	Venlafaxine No events	Placebo Events	Placebo No events	Risk ratio with 95% CI	Weight (%)
600-B-367-EU (75 mg)	1	82	1	40	0.49 [0.03, 7.70]	42.42
600-B-367-EU (150 mg)	3	79	1	41	1.54 [0.16, 14.32]	41.91
Guelfi 1995	2	44	0	47	5.11 [0.25, 103.55]	15.68
**Overall**					1.65 [0.42, 6.58]	

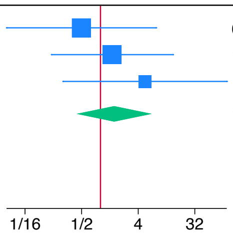

Heterogeneity: $I^2 = 0.00\%$, $H^2 = 1.00$

Test of $\theta_i = \theta_j$: Q(2) = 1.29, p = 0.53

Test of $\theta = 0$: z = 0.71, p = 0.47

Fixed-effects Mantel–Haenszel model

Supplementary Figure 120: Meta-analysis of venlafaxine versus placebo on increased salivation (sensitivity analysis)

Study	Venlafaxine Events	Venlafaxine No events	Placebo Events	Placebo No events	Risk ratio with 95% CI	Weight (%)
600-B-367-EU (75 mg)	1	82	0	41	1.50 [0.06, 36.04]	36.63
600-B-367-EU (150 mg)	0	82	0	42	0.52 [0.01, 25.66]	36.19
Guelfi 1995	1	45	0	47	3.06 [0.13, 73.33]	27.18
**Overall**					1.57 [0.25, 9.94]	

Heterogeneity: I^2 = 0.00%, H^2 = 1.00

Test of $\theta_i = \theta_j$: Q(2) = 0.48, p = 0.79

Test of θ = 0: z = 0.48, p = 0.63

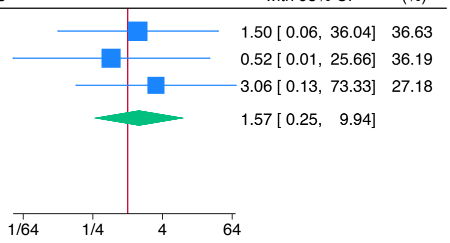

Fixed-effects Mantel–Haenszel model

Supplementary Figure 121: Meta-analysis of venlafaxine versus placebo on tongue discolouration (sensitivity analysis)

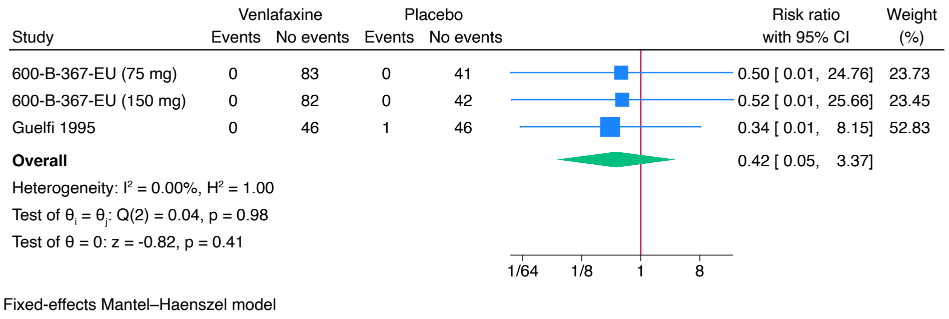

Supplementary Figure 122: Meta-analysis of venlafaxine versus placebo on hypochromic anaemia (sensitivity analysis)

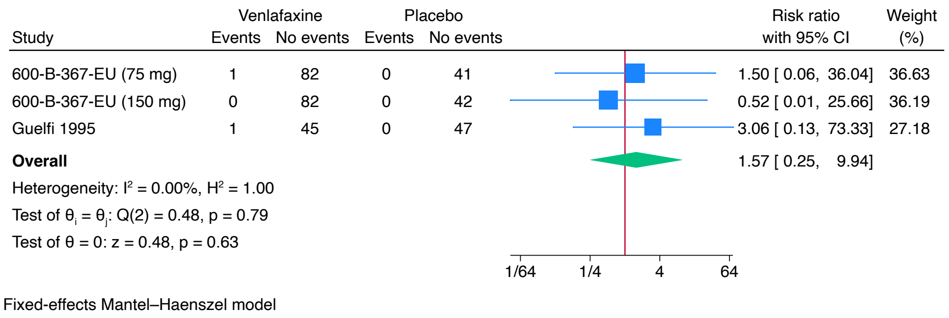

STATA

Supplementary Figure 123: Meta-analysis of venlafaxine versus placebo on hypercholesterolemia (sensitivity analysis)

Study	Venlafaxine Events	Venlafaxine No events	Placebo Events	Placebo No events	Risk ratio with 95% CI	Weight (%)
600-B-367-EU (75 mg)	0	83	0	41	0.50 [0.01, 24.76]	20.18
600-B-367-EU (150 mg)	0	82	0	42	0.52 [0.01, 25.66]	19.94
Guelfi 1995	1	45	2	45	0.51 [0.05, 5.44]	59.88
**Overall**					0.51 [0.08, 3.08]	

Heterogeneity: $I^2 = 0.00\%$, $H^2 = 1.00$

Test of $\theta_i = \theta_j$: Q(2) = 0.00, p = 1.00

Test of $\theta = 0$: z = -0.73, p = 0.46

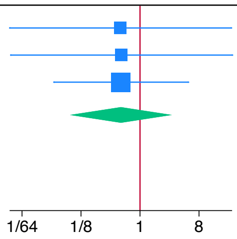

Fixed-effects Mantel–Haenszel model

Supplementary Figure 124: Meta-analysis of venlafaxine versus placebo on bronchitis (sensitivity analysis)

Study	Venlafaxine Events	Venlafaxine No events	Placebo Events	Placebo No events	Risk ratio with 95% CI	Weight (%)
600-B-367-EU (75 mg)	2	81	0	41	2.50 [0.12, 50.91]	36.63
600-B-367-EU (150 mg)	0	82	0	42	0.52 [0.01, 25.66]	36.19
Guelfi 1995	1	45	0	47	3.06 [0.13, 73.33]	27.18
**Overall**					1.94 [0.32, 11.86]	

Heterogeneity: $I^2 = 0.00\%$, $H^2 = 1.00$

Test of $\theta_i = \theta_j$: Q(2) = 0.55, p = 0.76

Test of $\theta = 0$: z = 0.71, p = 0.48

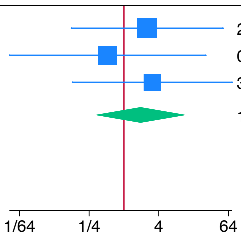

Fixed-effects Mantel–Haenszel model

Supplementary Figure 125: Meta-analysis of venlafaxine versus placebo on pharyngitis (sensitivity analysis)

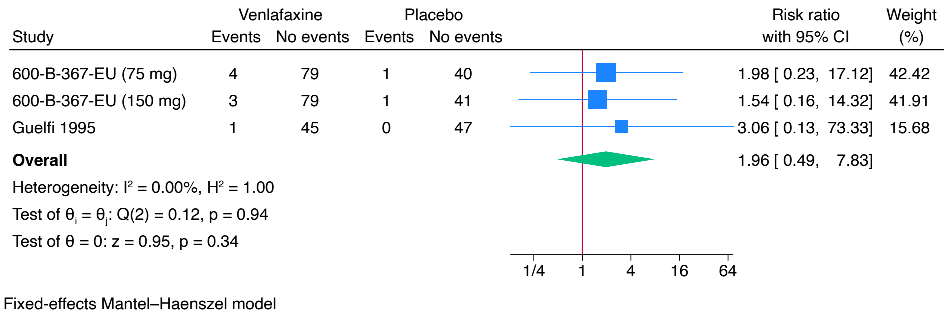

Supplementary Figure 126: Meta-analysis of venlafaxine versus placebo on urinary tract infection (sensitivity analysis)

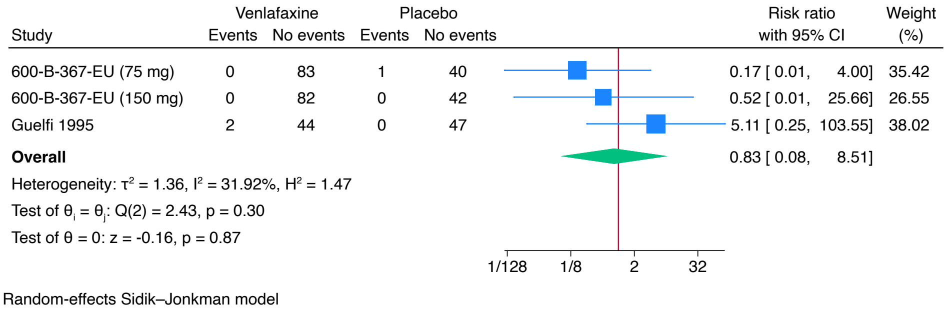

Supplementary Figure 127: Meta-analysis of venlafaxine versus placebo on urine abnormality (sensitivity analysis)

Study	Venlafaxine Events	Venlafaxine No events	Placebo Events	Placebo No events	Risk ratio with 95% CI	Weight (%)
600-B-367-EU (75 mg)	0	83	0	41	0.50 [0.01, 24.76]	24.86
600-B-367-EU (150 mg)	0	82	0	42	0.52 [0.01, 25.66]	24.86
Guelfi 1995	1	45	1	46	1.02 [0.07, 15.85]	50.28
**Overall**					0.72 [0.10, 5.07]	

Heterogeneity: $\tau^2 = 0.01$, $I^2 = 0.20\%$, $H^2 = 1.00$

Test of $\theta_i = \theta_j$: Q(2) = 0.12, p = 0.94

Test of $\theta = 0$: z = -0.33, p = 0.74

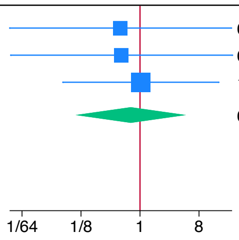

Random-effects Sidik–Jonkman model

Supplementary Figure 128: Meta-analysis of venlafaxine versus placebo on taste alteration (sensitivity analysis)

Study	Venlafaxine Events	Venlafaxine No events	Placebo Events	Placebo No events	Risk ratio with 95% CI	Weight (%)
600-B-367-EU (75 mg)	0	83	1	40	0.17 [0.01, 4.00]	35.42
600-B-367-EU (150 mg)	0	82	0	42	0.52 [0.01, 25.66]	26.55
Guelfi 1995	2	44	0	47	5.11 [0.25, 103.55]	38.02
**Overall**					0.83 [0.08, 8.51]	

Heterogeneity: $\tau^2 = 1.36$, $I^2 = 31.92\%$, $H^2 = 1.47$

Test of $\theta_i = \theta_j$: $Q(2) = 2.43$, $p = 0.30$

Test of $\theta = 0$: $z = -0.16$, $p = 0.87$

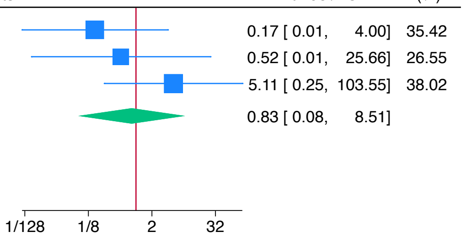

Random-effects Sidik–Jonkman model

Supplementary Figure 129: Meta-analysis of venlafaxine versus placebo on HDRS-17 (sensitivity analysis)

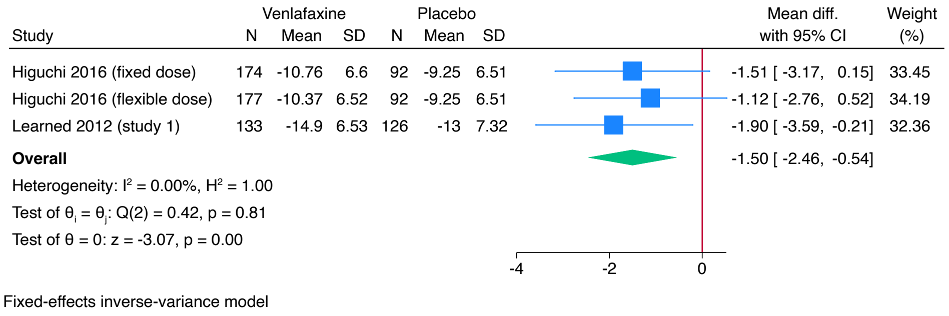

Supplementary Figure 130: Meta-analysis of venlafaxine versus placebo on suicidal ideation (sensitivity analysis)

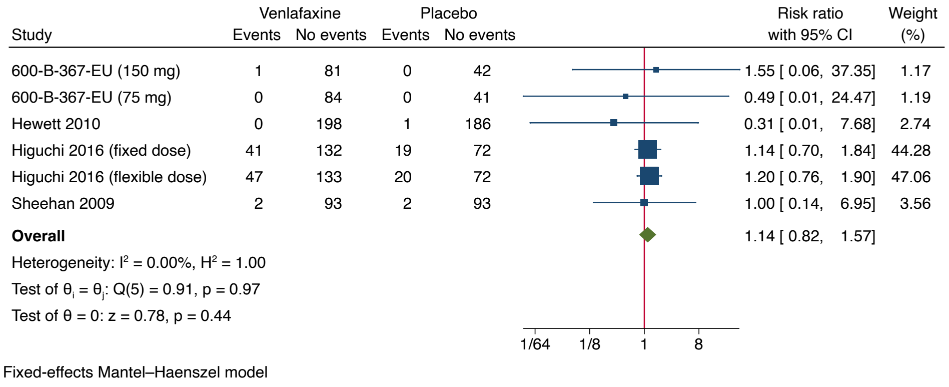

Supplementary Figure 131: Meta-analysis of venlafaxine versus placebo on MADRS (sensitivity analysis)

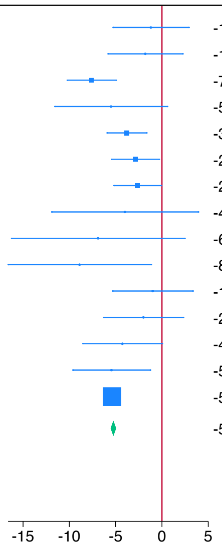

Study	Venlafaxine N	Venlafaxine Mean	Venlafaxine SD	Placebo N	Placebo Mean	Placebo SD	Mean diff. with 95% CI	Weight (%)
600-B-367-EU (75 mg)	53	8.2	9.5	27	9.4	7.3	-1.20 [-5.29, 2.89]	0.54
600-B-367-EU (150 mg)	48	7.6	9	26	9.4	7.3	-1.80 [-5.83, 2.23]	0.55
Alvarez 2012	95	-24.2	8.8	88	-16.6	9.4	-7.60 [-10.24, -4.96]	1.29
Guelfi 1995	36	13.6	10.5	23	19.1	13.2	-5.50 [-11.58, 0.58]	0.24
Hewett 2010	193	-17	10.56	186	-13.2	10.64	-3.80 [-5.93, -1.67]	1.97
Higuchi 2016 (fixed dose)	172	-15.3	10.1	91	-12.41	10.12	-2.89 [-5.46, -0.32]	1.36
Higuchi 2016 (flexible dose)	176	-15.05	10.08	91	-12.41	10.12	-2.64 [-5.19, -0.09]	1.37
Khan 1991 (75 mg)	23	21.3	9.7	9	25.3	11.7	-4.00 [-11.92, 3.92]	0.14
Khan 1991 (225 mg)	22	18.4	11.5	8	25.3	11.7	-6.90 [-16.25, 2.45]	0.10
Khan 1991 (375 mg)	22	16.4	9.2	9	25.3	11.7	-8.90 [-16.62, -1.18]	0.15
Mendels 1993 (25 mg)	78	-11.53	9.82	25	-10.53	8.98	-1.00 [-5.34, 3.34]	0.48
Mendels 1993 (50 - 75 mg)	72	-12.53	9.6	25	-10.53	8.98	-2.00 [-6.30, 2.30]	0.48
Mendels 1993 (150 - 200 mg)	77	-14.8	9.64	25	-10.53	8.98	-4.27 [-8.55, 0.01]	0.49
Sheehan 2009	53	13.53	10.31	55	18.98	11.78	-5.45 [-9.63, -1.27]	0.51
Thase 1997	91	15.2	1.14	100	20.6	1.08	-5.40 [-5.71, -5.09]	90.33
**Overall**							-5.24 [-5.54, -4.95]	

Heterogeneity: I^2 = 47.57%, H^2 = 1.91

Test of $\theta_i = \theta_j$: Q(14) = 26.70, p = 0.02

Test of θ = 0: z = -34.35, p = 0.00

Fixed-effects inverse-variance model

Supplementary Table 1: PRISMA 2020 Checklist

Section and Topic	Item #	Checklist item	Location where item is reported
**TITLE**			
Title	1	Identify the report as a systematic review.	Title
**ABSTRACT**			
Abstract	2	See the PRISMA 2020 for Abstracts checklist.	Abstract
**INTRODUCTION**			
Rationale	3	Describe the rationale for the review in the context of existing knowledge.	Introduction
Objectives	4	Provide an explicit statement of the objective(s) or question(s) the review addresses.	Introduction
**METHODS**			
Eligibility criteria	5	Specify the inclusion and exclusion criteria for the review and how studies were grouped for the syntheses.	Selection criteria
Information sources	6	Specify all databases, registers, websites, organisations, reference lists and other sources searched or consulted to identify studies. Specify the date when each source was last searched or consulted.	Search strategy
Search strategy	7	Present the full search strategies for all databases, registers and websites, including any filters and limits used.	Search strategy & Supplementary
Selection process	8	Specify the methods used to decide whether a study met the inclusion criteria of the review, including how many reviewers screened each record and each report retrieved, whether they worked independently, and if applicable, details of automation tools used in the process.	Data extraction and risk of bias assessment
Data collection process	9	Specify the methods used to collect data from reports, including how many reviewers collected data from each report, whether they worked independently, any processes for obtaining or confirming data from study investigators, and if applicable, details of automation tools used in the process.	Data extraction and risk of bias assessment
Data items	10a	List and define all outcomes for which data were sought. Specify whether all results that were compatible with each outcome domain in each study were sought (e.g. for all measures, time points, analyses), and if not, the methods used to decide which results to collect.	Outcomes and Subgroup analysis
	10b	List and define all other variables for which data were sought (e.g. participant and intervention characteristics, funding sources). Describe any assumptions made about any missing or unclear information.	Protocol
Study risk of bias assessment	11	Specify the methods used to assess risk of bias in the included studies, including details of the tool(s) used, how many reviewers assessed each study and whether they worked independently, and if applicable, details of automation tools used in the process.	Data extraction and risk of bias assessment
Effect measures	12	Specify for each outcome the effect measure(s) (e.g. risk ratio, mean difference) used in the synthesis or presentation of results.	Protocol
Synthesis methods	13a	Describe the processes used to decide which studies were eligible for each synthesis (e.g. tabulating the study intervention characteristics and comparing against the planned groups for each synthesis (item #5)).	Protocol
	13b	Describe any methods required to prepare the data for presentation or synthesis, such as handling of missing summary statistics, or data conversions.	Protocol
	13c	Describe any methods used to tabulate or visually display results of individual studies and syntheses.	Assessment of statistical and clinical significance

Supplementary Table 1: PRISMA 2020 Checklist

Section and Topic	Item #	Checklist item	Location where item is reported
	13d	Describe any methods used to synthesize results and provide a rationale for the choice(s). If meta-analysis was performed, describe the model(s), method(s) to identify the presence and extent of statistical heterogeneity, and software package(s) used.	Assessment of statistical and clinical significance
	13e	Describe any methods used to explore possible causes of heterogeneity among study results (e.g. subgroup analysis, meta-regression).	Protocol
	13f	Describe any sensitivity analyses conducted to assess robustness of the synthesized results.	Protocol & Results
Reporting bias assessment	14	Describe any methods used to assess risk of bias due to missing results in a synthesis (arising from reporting biases).	Protocol
Certainty assessment	15	Describe any methods used to assess certainty (or confidence) in the body of evidence for an outcome.	Protocol
**RESULTS**			
Study selection	16a	Describe the results of the search and selection process, from the number of records identified in the search to the number of studies included in the review, ideally using a flow diagram.	Figure 1
	16b	Cite studies that might appear to meet the inclusion criteria, but which were excluded, and explain why they were excluded.	Results
Study characteristics	17	Cite each included study and present its characteristics.	Table 1
Risk of bias in studies	18	Present assessments of risk of bias for each included study.	Results
Results of individual studies	19	For all outcomes, present, for each study: (a) summary statistics for each group (where appropriate) and (b) an effect estimate and its precision (e.g. confidence/credible interval), ideally using structured tables or plots.	Results
Results of syntheses	20a	For each synthesis, briefly summarise the characteristics and risk of bias among contributing studies.	Results
	20b	Present results of all statistical syntheses conducted. If meta-analysis was done, present for each the summary estimate and its precision (e.g. confidence/credible interval) and measures of statistical heterogeneity. If comparing groups, describe the direction of the effect.	Results
	20c	Present results of all investigations of possible causes of heterogeneity among study results.	Results
	20d	Present results of all sensitivity analyses conducted to assess the robustness of the synthesized results.	Results & Supplementary
Reporting biases	21	Present assessments of risk of bias due to missing results (arising from reporting biases) for each synthesis assessed.	Results
Certainty of evidence	22	Present assessments of certainty (or confidence) in the body of evidence for each outcome assessed.	Results
**DISCUSSION**			
Discussion	23a	Provide a general interpretation of the results in the context of other evidence.	Discussion
	23b	Discuss any limitations of the evidence included in the review.	Discussion
	23c	Discuss any limitations of the review processes used.	Discussion
	23d	Discuss implications of the results for practice, policy, and future research.	Discussion
**OTHER INFORMATION**			
Registration and	24a	Provide registration information for the review, including register name and registration number, or state that the review was not registered.	Methods

Supplementary Table 1: PRISMA 2020 Checklist

Section and Topic	Item #	Checklist item	Location where item is reported
protocol	24b	Indicate where the review protocol can be accessed, or state that a protocol was not prepared.	Methods
	24c	Describe and explain any amendments to information provided at registration or in the protocol.	Differences between the protocol and the review
Support	25	Describe sources of financial or non-financial support for the review, and the role of the funders or sponsors in the review.	Financial Support & Competing Interests
Competing interests	26	Declare any competing interests of review authors.	Competing Interests
Availability of data, code and other materials	27	Report which of the following are publicly available and where they can be found: template data collection forms; data extracted from included studies; data used for all analyses; analytic code; any other materials used in the review.	Data used for all analyses are available in the results.

From: Page MJ, McKenzie JE, Bossuyt PM, Boutron I, Hoffmann TC, Mulrow CD, et al. The PRISMA 2020 statement: an updated guideline for reporting systematic reviews. BMJ 2021;372:n71. doi: 10.1136/bmj.n71

For more information, visit: http://www.prisma-statement.org/

Supplementary Table 2: Search strategies

Search strategies for
'Venlafaxine or Mirtazapine for major depressive disorder'
(C Kamp)
Updated searches performed 7 March 2024

**Total number of records identified:**	**10691 records**
**Number of duplicates excluded:**	**2770 records**
**Number of records in final list:**	**7921 records**
**Number of new records sent to authors:**	**763 records**

Cochrane Central Register of Controlled Trials (2024, Issue 2) in the Cochrane Library (813 hits)
#1 MeSH descriptor: [Venlafaxine Hydrochloride] explode all trees
#2 MeSH descriptor: [Mirtazapine] explode all trees
#3 (venlafaxin* or ef*exor* or mirtazapin* or org*3770 or remeron*)
#4 #1 or #2 #3
#5 MeSH descriptor: [Depressive Disorder, Major] explode all trees
#6 MeSH descriptor: [Depressive Disorder] this term only
#7 MeSH descriptor: [Seasonal Affective Disorder] explode all trees
#8 MeSH descriptor: [Dysthymic Disorder] explode all trees
#9 MeSH descriptor: [Depression] explode all trees
#10 MeSH descriptor: [Affective Symptoms] this term only
#11 ((depress* or affective or dysthym*) and (disorder* or disease* or symptom*))
#12 #5 or #6 or #7 or #8 or #9 or #10 or #11
#13 #4 and #12

MEDLINE Ovid (1946 to 7 March 2024) (2963 hits)
1. exp Venlafaxine Hydrochloride/
2. exp Mirtazapine/
3. (venlafaxin* or ef*exor* or mirtazapin* or org*3770 or remeron*).mp. [mp=title, abstract, original title, name of substance word, subject heading word, floating sub-heading word, keyword heading word, organism supplementary concept word, protocol supplementary concept word, rare disease supplementary concept word, unique identifier, synonyms]
4. 1 or 2 or 3
5. exp Depressive Disorder, Major/
6. Depressive Disorder/
7. exp Seasonal Affective Disorder/
8. exp Dysthymic Disorder/
9. exp Depression/
10. Affective Symptoms/
11. ((depress* or affective or dysthym*) and (disorder* or disease* or symptom*)).mp. [mp=title, abstract, original title, name of substance word, subject heading word, floating sub-heading word, keyword heading word, organism supplementary concept word, protocol supplementary concept word, rare disease supplementary concept word, unique identifier, synonyms]
12. 5 or 6 or 7 or 8 or 9 or 10 or 11
13. 4 and 12
14. (randomized controlled trial or controlled clinical trial).pt. or clinical trials as topic.sh. or trial.ti.

15. (random* or blind* or placebo* or meta-analys*).mp. [mp=title, abstract, original title, name of substance word, subject heading word, floating sub-heading word, keyword heading word, organism supplementary concept word, protocol supplementary concept word, rare disease supplementary concept word, unique identifier, synonyms]
16. 13 and (14 or 15)
17. limit 16 to ("adolescent (13 to 18 years)" or "young adult (19 to 24 years)" or "adult (19 to 44 years)" or "young adult and adult (19-24 and 19-44)" or "middle age (45 to 64 years)" or "middle aged (45 plus years)" or "all aged (65 and over)" or "aged (80 and over)")

Embase Ovid (1974 to 7 March 2024) (5650 hits)

1. exp venlafaxine/
2. exp mirtazapine/
3. (venlafaxin* or ef*exor* or mirtazapin* or org*3770 or remeron*).mp. [mp=title, abstract, heading word, drug trade name, original title, device manufacturer, drug manufacturer, device trade name, keyword heading word, floating subheading word, candidate term word]
4. 1 or 2 or 3
5. exp major depression/
6. depression/
7. exp seasonal affective disorder/
8. exp dysthymia/
9. emotional disorder/
10. ((depress* or affective or dysthym*) and (disorder* or disease* or symptom*)).mp. [mp=title, abstract, heading word, drug trade name, original title, device manufacturer, drug manufacturer, device trade name, keyword heading word, floating subheading word, candidate term word]
11. 5 or 6 or 7 or 8 or 9 or 10
12. 4 and 11
13. Randomized controlled trial/ or Controlled clinical trial/ or trial.ti.
14. (random* or blind* or placebo* or meta-analys*).mp. [mp=title, abstract, heading word, drug trade name, original title, device manufacturer, drug manufacturer, device trade name, keyword heading word, floating subheading word, candidate term word]
15. 12 and (13 or 14)
16. limit 15 to (adult <18 to 64 years> or aged <65+ years>)

LILACS (Bireme; 1982 to 7 March 2024) (50 hits)

((mh:(venlafaxine hydrochloride OR d02.033.415.510.500.901 OR d02.092.471.683.948 OR d02.455.426.392.368.367.318.750 OR d10.289.510.500.901 OR mirtazapine OR d03.633.300.240.588)) OR ((venlafaxin* OR ef*exor* OR mirtazapin* OR org*3770 OR remeron*))) AND ((mh:(depressive disorder, major OR f03.600.300.375 OR depressive disorder OR f03.600.300 OR seasonal affective disorder OR f03.600.300.775 OR dysthymic disorder OR f03.600.300.400 OR depression OR f01.145.126.350 OR f01.470.282 OR affective symptoms OR f01.145.126.100)) OR (((depress* OR affective OR dysthym*) AND (disorder* OR disease* OR symptom*)))) AND (db:("LILACS"))

PsycINFO (EBSCO host; 1806 to 7 March 2024) (562 hits)

S17 S15 AND S16
S16 TI adult* or Elder* or older or Geriatri* or Senil* or Old Age* or Late Life or Aged OR AB adult* or Elder* or older or Geriatri* or Senil* or Old Age* or Late Life or Aged
S15 S13 AND S14
S14 TX ((random* or blind* or placebo* or meta-analys*)) OR TI trial*
S13 S4 AND S12
S12 S5 OR S6 OR S7 OR S8 OR S9 OR S10 OR S11
S11 TX ((depress* or affective or dysthym*) and (disorder* or disease* or symptom*))
S10 MA Affective Symptoms
S9 MA Depression

S8 MA Dysthymic Disorder<br>S7 MA Seasonal Affective Disorder<br>S6 MA Depressive Disorder Expanders<br>S5 MA Depressive Disorder, Major<br>S4 S1 OR S2 OR S3<br>S3 TX (venlafaxin* or effexor* or efexor* or mirtazapin* or "org 3770" or org3770 or org-3770 or remeron*)<br>S2 MA mirtazapine<br>S1 MA venlafaxine
**Science Citation Index Expanded (Web of Science; 1900 to 7 March 2024); Conference Proceedings Citation Index – Science (Web of Science; 1990 to 7 March 2024); Social Sciences Citation Index (Web of Science; 1956 to 7 March 2024), and Conference Proceedings Citation Index- Social Science & Humanities (Web of Science; 1990 to 7 March 2024) (653 hits)**<br>#7 #5 AND #6<br>#6 TS=(adult* or Elder* or older or Geriatri* or Senil* or Old Age* or Late Life or Aged)<br>#5 #3 AND #4<br>#4 TI=(random* or blind* or placebo* or meta-analys* or trial*) OR TS=(random* or blind* or placebo* or meta-analys*)<br>#3 #2 AND #1<br>#2 TS=((depress* or affective or dysthym*) and (disorder* or disease* or symptom*))<br>#1 TS=(venlafaxin* or ef*exor* or mirtazapin* or org*3770 or remeron*)

Supplementary Table 3: Characteristics of the included trials

Trial ID	Registry/ published protocol	Risk of for-profit bias	Inclusion criteria	Exclusion criteria	Dose range (mg/day)	Control intervention	Placebo washout	Length of intervention period	No. randomised to Venlafaxine	No. randomised to control	Baseline HDRS Venlafaxine	Baseline HDRS control	Co-interventions
0600B 1-384-US/EU/CA	No	Yes	NI	NI	150 - 375 mg/day	Placebo	NI	6 weeks	180	83	NI	NI	NI
600-B-367-EU (150 mg)	No	Yes	I. Were 18 years of age and of legal age of consent or older<br>2. Were outpatients<br>3. Met DSM-III-R criteria for major depression; had a minimum screening and<br>baseline score of 20 on the HAM-D total score<br>4. Had symptoms of depression for at least 1month before entry into the study.<br>5. Signed informed consent form.	1. Had a decrease of more than 20% in the HAM-D total score between the screening and baseline visits.<br>2. Had a myocardial infarction within 6 months of the start of double-blind treatment.<br>3. Had a history or the presence of clinically significant hepatic or renal disease or other medical disease that may have compromised the study.<br>4. Had a history of seizure disorder other than a single childhood febrile seizure.<br>5. Had a history or presence of any psychotic disorder not associated with depression. ·<br>6. Had a history or presence of bipolar disorder.<br>7. Had a history or presence of organic mental disorder.<br>8. Was acutely suicidal to such a degree that precautions against suicide were required to be employed.<br>9. Was a lactating woman or a woman of childbearing potential with a positive beta-HCG test result <luring the prestudy evaluation.<br>Used any investigational drug, antipsychotic drug, electroconvulsive therapy (ECT) within 30 days, fluoxetine within 21 days; or used any MAO inhibitor. paroxetine, or sertraline within 14 days; or used any other antidepressant, anxiolytic, sedative-hypnotic drug (except chloral hydrate) or any other psychotropic drug or substance within 7 days of the start of the double-blind treatment period.<br>Used any nonpsychopharmacologic drug with psychotropic effects within 7 days of the start of the double-blind treatment period unless a stable dose of the drug has been maintained for at least 1month before the start of the double-blind treatment period.<br>Had a history of drug or alcohol dependence within 1year as defined by DSM-III-R criteria. Had clinically significant abnormalities on the prestudy physical examination, ECG laboratory tests or urine drug screen.	Mean: 150 mg/day	Placebo	Yes	8 weeks	82	41	27.1	26.6	No
600-B-367-EU (75 mg)	No	Yes	I. Were 18 years of age and of legal age of consent or older<br>2. Were outpatients<br>3. Met DSM-III-R criteria for major depression; had a minimum screening and<br>baseline score of 20 on the HAM-D total score<br>4. Had symptoms of depression for at least 1 month before entry into the study.<br>5. Signed informed consent form.	1. Had a decrease of more than 20% in the HAM-D total score between the screening and baseline visits.<br>2. Had a myocardial infarction within 6 months of the start of double-blind treatment.<br>3. Had a history or the presence of clinically significant hepatic or renal disease or other medical disease that may have compromised the study.<br>4. Had a history of seizure disorder other than a single childhood febrile seizure.<br>5. Had a history or presence of any psychotic disorder not associated with depression.<br>6. Had a history or presence of bipolar disorder.<br>7. Had a history or presence of organic mental disorder.<br>8. Was acutely suicidal to such a degree that precautions against suicide were required to be employed.<br>9. Was a lactating woman or a woman of childbearing potential with a positive beta-HCG test result <luring the prestudy evaluation.<br>Used any investigational drug, antipsychotic drug, electroconvulsive therapy (ECT) within 30 days, fluoxetine within 21 days; or used any MAO inhibitor. paroxetine, or sertraline within 14 days; or used any other antidepressant, anxiolytic, sedative-hypnotic drug (except chloral hydrate) or any other psychotropic drug or substance within 7 days of the start of the double-blind treatment period.<br>Used any nonpsychopharmacologic drug with psychotropic effects within 7 days of the start of the double-blind treatment period unless a stable dose of the drug has been maintained for at least 1month before the start of the double-blind treatment period.<br>Had a history of drug or alcohol dependence within 1 year as defined by DSM-III-R criteria. Had clinically significant abnormalities on the prestudy physical examination, ECG laboratory tests or urine drug screen.	Mean: 75 mg/day	Placebo	Yes	8 weeks	83	42	26.5	4.7	No
Alvarez 2012	Yes	Yes	Patients with MDD presenting with a current major depressive episode according to DSM-IV-TR criteria (APA, 1994) were included in the study if they were an outpatient of either sex, aged from 18 yr to 65 yr, with a Montgomery–Asberg Depression Rating Scale (MADRS) (Montgomery & Åsberg, 1979) total score of 30 at the baseline visit.	Patients were excluded if they had any current psychiatric disorder other than MDD as defined in DSM-IV-TR [assessed using the Mini International Neuropsychiatric Interview (MINI ; Sheehan et al. 1998)], or if they had a current or past history of manic or hypomanic episode, schizophrenia or any other psychotic disorder, including major depression with psychotic features, mental retardation, organic mental disorders, or mental disorders due to a general medical condition, any substance abuse disorder within the previous 6 months, presence or history of a clinically significant neurological disorder (including epilepsy), any neurodegenerative disorder, or any Axis II disorder that might compromise the study.<br>Patients at serious risk of suicide, based on the investigator's clinical judgement, or who had a score of of 5 on item 10 of the MADRS scale (suicidal thoughts) were also excluded, as were those receiving formal behaviour therapy or systematic psychotherapy, or were pregnant or breastfeeding, had a known hypersensitivity or were non-response to venlafaxine, or whose current depressive symptoms were considered by the investigator to have been resistant to two adequate antidepressant treatments of at least 6 wk duration, or had previously been exposed to Lu AA21004. Patients were also excluded if they were taking the following psychotropic drugs within 2 wk prior to baseline or during the study: Reversible or irreversible monoamine oxidase inhibitors, SSRIs (fluoxetine within 5 wk), SNRIs, tricyclic antidepressants, psychoactive herbal remedies, any drug used for augmentation of antidepressant action or any other antidepressant drugs, oral antipsychotic and anti-manic drugs, or dopamine antagonists, any anxiolytics (including benzodiazepines) ; and any anticonvulsant drug, serotonergic agonists, narcotic analgesics or cough agents, anti-arrhythmics, oral anticoagulants, proton pump inhibitors, steroids, cisapride, macrolide antibiotics, antifungal agents, antihypertensives, all anti-inflammatory agents, anti-migraine agents, pseudoephedrine, hypolipidaemics, and episodic use of insulin. Occasional use of zolpidem, zopiclone and zaleplon for insomnia was allowed.	75-225 mg/day	Placebo	No	6 weeks	114	105	29.4	29.7	No
Claghorn 1990	No	Yes	NI	NI	75-225 mg/d	Placebo	Yes	6 weeks	83	82	23.6	24.6	No
Cunningham 1994	No	No	Patients whose HAM-D score dropped 20% or more between screen and were excluded from the double-blind phase. Patients were also excluded if they had unstable medical conditions, significant laboratory or EKG abnormalities, history of seizure disorder, any psychotic disorder not associated with depression, or a history of drug or alcohol dependence within 2 years of entering the study. Patients could not present a serious suicide risk or receive formal psychotherapy during the study period. Other exclusion criteria included the use of any investigational or antipsychotic drug within 30 days, any investigational or antipsychotic drug within 30 days, any monoamine oxidase inhibitor or electroconvulsive therapy within 14 days, or any other antidepressant, anxiolytic, or hypnotic drug within 7 days before the start of or during the double-blind treatment period.	Patients included women or men 18 years of age or older. All women of child-bearing potential had to have negative β-human chorionic gonadotropin pregnancy test before receiving double-blind medication and agree to use effective contraception until the completion of the protocols. All patients met DSM-lll-R criteria for major depression, either single or recurrent episode, except that they must have had symptoms for a minimum of 1 month before the initial visit. Diagnosis was made by a psychiatrist on the basis of an unstructured clinical interview. A minimum 21-item Hamilton Rating Scale for Depression (HAM-D) score of 20 was required at both the initial screening visit and at the baseline visit before randomization.	75-200 mg/day	Placebo	Yes	6 weeks	Unclear	Unclear	25.02	24.41	No
Cunningham 1997 (IR)	No	Yes	Outpatients aged 18 years or older who met DSM-III-R criteria for a major depressive episode; had a minimum baseline score of 20 on the 21-item Hamilton Depression Rating Scale (HAM-D)(12), with not more than a 20% decrease in score between screening and baseline; and had symptoms of depression for at least one month before study entry were eligible.	Patients were excluded if they had previously been treated with venlafaxine. Women who were lactating or of childbearing potential with a positive β- human chorionic gonadotropin (HCG) pregnancy test were not included. In addition, patients with a history of clinically significant medical disease or clinically significant abnormalities on a screening physical examination, electrocardiogram (ECG), or laboratory tests; acute suicidal tendencies; a history of a seizure disorder; presence of an organic mental disorder; bipolar disorder; or a history of any psychotic disorder not associated with depression were excluded. Other reasons for exclusion were use of any investigational drug, antipsychotic drug, or electroconvulsive therapy within 30 days, fluoxetine within 21 days, or monoamine oxidase inhibitor, paroxetine, or sertraline within 14 days, or use of any other antidepressant, anxiolytic, sedative-hypnotic drug, or psychotropic drug or substance within 7 days of the start of double-blind treatment; use of any nonpsychotropic drug with psychotropic effects (e.g., β-adrenergic blockers), unless the dosage had been stable for a minimum of one month prior to double-blind treatment; or a history of drug or alcohol abuse within 1 year of the start of the study.	Max: 150 mg/day	Placebo	Yes	12 weeks	Unclear	Unclear	24.0	24.9	No
Cunningham 1997 (XR)	No	Yes	Outpatients aged 18 years or older who met DSM-III-R criteria for a major depressive episode; had a minimum baseline score of 20 on the 21-item Hamilton Depression Rating Scale (HAM-D)(12), with not more than a 20% decrease in score between screening and baseline; and had symptoms of depres- sion for at least one month before study entry were eligible.	Patients were excluded if they had previously been treated with venlafaxine. Women who were lactating or of childbearing potential with a positive P- human chorionic gonadotropin (HCG) pregnancy test were not included. In addition, patients with a history of clinically significant medical disease or clinically significant abnormalities on a screening physical examination, electrocardiogram (ECG), or laboratory tests; acute suicidal tendencies; a history of a seizure disorder; presence of an organic mental disorder; bipolar disorder; or a history of any psychotic disorder not associated with depression were excluded. Other reasons for exclusion were use of any investigational drug, antipsychotic drug, or electroconvulsive therapy within 30 days, fluoxetine within 21 days, or monoamine oxidase inhibitor, paroxetine, or sertraline within 14 days, or use of any other antidepressant, anxiolytic, sedative-hypnotic drug, or psychotropic drug or substance within 7 days of the start of double-blind treatment; use of any nonpsychotropic drug with psychotropic effects (e.g., p-adrenergic blockers), unless the dosage had been stable for a minimum of one month prior to double-blind treatment; or a history of drug or alcohol abuse within 1 year of the start of the study.	Max: 150 mg/day	Placebo	Yes	12 weeks	Unclear	Unclear	24.5	24.9	No
EudraCT 2004-000562-13	Yes	Yes	Men and women 18 to 75 years of age, inclusive; outpatients; subjects must have had a primary diagnosis of MDD based on the criteria in the Diagnostic and Statistical Manual of Mental Disorders, fourth edition, single or recurrent episode, without psychotic features. If other allowable psychiatric diagnoses were present, MDD must have been the predominant psychiatric disorder present. At the Screening and Baseline visits, subjects were required to have depressive symptoms for at least 30 days, a score of at least 22 on the Hamilton Psychiatric Rating Scale for Depression, 17-items (HAM-D17), a score of at least 2 on item 1 (depressed mood) of the HAM-D17, and a score of at least 4 on the Clinical Global Impressions Scale-Severity of Illness (CGI-S). Sexually active subjects had to use a medically acceptable form of contraception during the study and for at least 15 days after the last dose of study drug.	Subjects treated with DVS SR at any time in the past, treated with venlafaxine (immediate release [IR] or ER) within 90 days of study Day 1, and subjects with know hypersensitivity to venlafaxine (IR or ER) were excluded from the study.	Max: 150 mg/day	Placebo	No	8 weeks	128	123	25.8	26.0	No
EudraCT 2007-007025-51	Yes	Yes	In- and outpatients with a primary diagnosis of Major Depressive Episode (MDE) according to DSM-IV-TRTM criteria, who:<br>• had an MDE of ≥3 months duration at screening<br>• had a Montgomery-Asberg Depression Rating Scale (MADRS) total score ≥26 at screening and at baseline<br>• were ≥18 and ≤75 years of age		Max: 225 mg/day	Placebo	No	8 weeks	7	7	Unclear	Unclear	No
Guelfi 1995	No	Yes	Inpatients with a primary diagnosis of depression, aged 18 or older, were enrolled if they met the DSM-lll-R criteria for major depression and melancholia based on a structured inventory. The patient were required to have a minimum prestudy and initial study day score of 25 or more on the Montgomery-Asberg Depression Scale (MADRS) and symptoms of depression for at least 1 month before they could enter the study.	Patients were ineligible for enrollment if they had significant physical or mental illnesses (other than depression), a myocardial infarction within 6 months of the study, a history of seizure disorder or any psychotic disorder not associated with depression, or a history of drug or alcohold dependence. In addition, patients who had a score of 4 or more on the suicidal thought item of the MADRS were excluded from entry. Investigational drugs, antipsychotics, other antidepressants, anxiolytics, and sedative-hypnotics were disallowed. Electroconvulsive therapy and formal psychotherapy (defined as regularly scheduled sessions) were prohibited.	150-375 mg/day	Placebo	Yes	4 weeks	46	47	28.2	28.6	No
Hewett 2009	No	Yes	Patients aged 18–64 years with a DSM-IV diagnosis of MDD for a minimum of 8 weeks were eligible for inclusion. Eligible patients required an Interactive Voice Response System (IVRS) Hamilton Depression Rating Scale (HAMD) 17-Item total score of ≥18 at both screening and baseline visits (Kobak, et al., 1999), which must not have decreased or increased by more than 25% between visits. A score of ≥4 on the Clinical Global Impressions–Severity of Illness (CGI-S) scale at both screening and baseline was also required. Patients with co-morbidities were allowed to enrol if their condition had been stable for at least 3 month	Patients were excluded if they had been homicidal at any time or suicidal within the past 6 months. Those with anorexia nervosa or bulimia (within the past 12 months), psychotic disorders, myo- cardial infarction within the past year, a seizure disorder or blood pressure ≥150/95 mmHg were also excluded. Patients were not eligible to participate if they had taken bupropion or venlafaxine within the past 6 months, or had experienced a significant adverse response to either antidepressant in the past. Patients who had failed to respond to adequate treatment from two previous anti- depressants of different classes were also excluded. To be eligible, during the 2 weeks prior to the study, patients should not have used the following: any psychotherapy or psychotropic drugs; other medications with potential pharmacokinetic interactions or any medication that might lower the seizure threshold. Study participants were required to test negative in a urine drug screen, and to have shown no evidence of alcohol or substance abuse/ dependence within the past 12 months.	75-150 mg/day	Placebo	No	8 weeks	Unclear	Unclear	Unclear	Unclear	No

Trial ID	Registry/ published protocol	Risk of for-profit bias	Inclusion criteria	Exclusion criteria	Dose range (mg/day)	Control intervention	Placebo washout	Length of intervention period	No. randomised to Venlafaxine	No. randomised to control	Baseline HDRS Venlafaxine	Baseline HDRS control	Co-interventions
Hewett 2010	No	Yes	Patients aged 18–64 years with a DSM-IV diagnosis of MDD for a minimum of eight weeks duration were eligible for inclusion. Eligible patients required an Interactive Voice Response System (IVRS) Hamilton Depression Rating Scale (HAMD) 17-item total score of ≥18 at both screening and baseline visits (Kobak et al., 1999), which must not have decreased or increased by more than 25% between visits. A score of ≥4 on the Clinical Global Impressions–Severity of Illness (CGI-S) scale at both screening and baseline was also required.	Patients were excluded if they had a history of manic episodes, past or current psychotic disorder or a current Axis II diagnosis that suggested non-responsiveness or non-compliance with therapy. Also excluded were patients that had been homicidal at any time in their lives or suicidal within the past 6 months, those with anorexia nervosa or bulimia within the past year, myocardial infarction within the past year, any history of seizure disorder or brain injury, blood pressure 150/95 mmHg, or unstable medical disorder. Patients were not eligible to participate if they had taken bupropion or venlafaxine within the past six months, or had experienced a significant adverse response to either antidepressant in the past. Patients who had failed to respond to adequate treatment from two previous antidepressants of different classes were also excluded. Had any psychotherapy or taken any psychotropic drugs, other medications with potential pharmacokinetic interactions, or any medication that might lower the seizure threshold in the two weeks prior to screening. Study participants were required to provide a negative urine drug screen, a blood alcohol level of <0.015% at screening, and to have shown no evidence of alcohol or substance abuse/dependence within the past 12 months.	75-150 mg/day	Placebo	No	8 weeks	Unclear	Unclear	30.1	30.6	No
Higuchi 2016 (fixed dose)	Yes	Yes	In the double-blinded study, outpatients aged at least 20 years with a primary diagnosis of MDD on the basis of the DSM-IV criteria, who experienced single or recurrent episodes without psychotic features, were eligible for the study. In addition, patients should have experienced depressive symptoms for at least 90 days in a single episode and for at least 28 days in a recurrent episode before the screening visit and have a Montgomery– Åsberg Depression Rating Scale (MADRS) total score of at least 26 at the screening and baseline visits with a change in MADRS total scores at baseline not beyond 25% from the screening visit, a 16-item Quick Inventory of Depressive Symptomatology self-report version (QIDS16-SR-J) total score of at least 16 at the screening and baseline visits (Rush et al., 2003), and a score of at least 4 on the Clinical Global Impressions Scale-Severity (CGI-S) at the screening and baseline visits. Moreover, they should have provided a personally signed and dated informed consent document indicating that they had been informed of all pertinent aspects of the study and were willing and able to comply with scheduled visits, treatment plan, laboratory tests, and other study procedures. All female and male patients who were biologically capable of having children had to agree and commit to the use of a reliable method of birth control during the study period and for 28 days after the last dose of study medication.	Patients who had received treatment with venlafaxine or desvenlafaxine in the past; a history of personality disorder or mental retardation, substance abuse, psychotic disorders, dementia, obsessive compulsive disorder, post-traumatic stress disorder, bipolar disorder, anxiety disorder, or active suicidal tendency; other clinically important medical conditions as determined by the investigators; or any other unstable medical condition such as cardiovascular disease were excluded. Patients who had been nonresponsive to two antidepressant treatments in the past, had a history of chronic treatment with benzodiazepines for longer than 6 months before the screening visit, or had depression associated with the presence of an organic mental disorder because of a general medical condition or a neurologic disorder were also excluded.	Mean: 75 mg/day	Placebo	No	8 weeks	Unclear	Unclear	22.6	22.4	No
Higuchi 2016 (flexible dose)	Yes	Yes	In the double-blinded study, outpatients aged at least 20 years with a primary diagnosis of MDD on the basis of the DSM-IV criteria, who experienced single or recurrent episodes without psychotic features, were eligible for the study. In addition, patients should have experienced depressive symptoms for at least 90 days in a single episode and for at least 28 days in a recurrent episode before the screening visit and have a Montgomery-Asberg Depression Rating Scale (MADRS) total score of at least 26 at the screening and baseline visits with a change in MADRS total scores at baseline not beyond 25% from the screening visit, a 16-item Quick Inventory of Depressive Symptomatology self-report version (QIDS16-SR-J) total score of at least 16 at the screening and baseline visits (Rush et al., 2003), and a score of at least 4 on the Clinical Global Impressions Scale-Severity (CGI-S) at the screening and baseline visits. Moreover, they should have provided a personally signed and dated informed consent document indicating that they had been informed of all pertinent aspects of the study and were willing and able to comply with scheduled visits, treatment plan, laboratory tests, and other study procedures. All female and male patients who were biologically capable of having children had to agree and commit to the use of a reliable method of birth control during the study period and for 28 days after the last dose of study medication.	Patients who had received treatment with venlafaxine or desvenlafaxine in the past; a history of personality disorder or mental retardation, substance abuse, psychotic disorders, dementia, obsessive compulsive disorder, post-traumatic stress disorder, bipolar disorder, anxiety disorder, or active suicidal tendency; other clinically important medical conditions as determined by the investigators; or any other unstable medical condition such as cardiovascular disease were excluded. Patients who had been nonresponsive to two antidepressant treatments in the past, had a history of chronic treatment with benzodiazepines for longer than 6 months before the screening visit, or had depression associated with the presence of an organic mental disorder because of a general medical condition or a neurologic disorder were also excluded.	75-225 mg/day	Placebo	No	8 weeks	Unclear	Unclear	22.4	22.4	No
Hopkins 2013	Yes	Yes	The duration of the current episode must be at least 1 month but not longer than 12 months.<br>Subjects must have a primary diagnosis of Major Depressive Disorder.<br>Subjects must have had at least one previous, diagnosed episode of MDD in the past 5 years.<br>MDD must be the condition that was chiefly responsible for motivating the subject to seek treatment.<br>Subject is in general good health.	Subject is participating in, has participated in, or plans to participate in any investigational drug study.<br>Subject who has donated blood within the last 30 days or plans to donate blood during and 30 days following participation.<br>Known failure to respond (in the past 5 years) to two adequate (dose and duration) antidepressant medications with distance mechanisms of action including tricyclics.<br>Subjects who have undergone Electroconvulsive Therapy treatment.<br>Treatment with fluoxetine, in the 6 weeks before baseline.<br>Subject with psychotic disorders, anorexia nervosa, bulimia or post-traumatic stress disorder.<br>Subject with a history or presence of bipolar disorder (i.e., current or past history of manic episode).<br>Subjects with Obsessive Compulsive Disorder.<br>Subjects with a lifetime diagnosis of Panic Disorder.<br>Subject received treatment with antidepressants within 2 weeks.<br>Subject with lifetime history of suicidal attempts, alcohol dependence or abuse, drug(s) dependence or abuse (excluding nicotine and caffeine) or has a positive urine drug screen.<br>Subject has a history of significant risk of suicide or homicide.<br>Bereavement - Defined as death of a loved one within 3 months.<br>Subject has a documented history of HIV, hepatitis B or hepatitis C.	Mean: 150 mg/day	Placebo	No	8 weeks	Unclear	Unclear	Unclear	Unclear	No
Hunter 2010 (study 2)	No	Yes	Subjects were excluded if they previously had failed to benefit from treatment with the antidepressant being studied, if they had a history of suicide attempt, or if they suffered from any medical illness or received any medication known to significantly affect brain function.	Subjects were excluded if they previously had failed to benefit from treatment with the antidepressant being studied, if they had a history of suicide attempt, or if they suffered from any medical illness or received any medication known to significantly affect brain function.	Mean: 150 mg/day	Placebo	Yes	8 weeks	Unclear	Unclear	Unclear	Unclear	No
Hunter 2010 (study 3)	No	Yes	Subjects were recruited through outpatient clinics and community advertisement and met MDD diagnostic criteria using a structured interview for DSM-IV (First et al., 1995), with a 17-item Hamilton Depression Rating Scale (Ham-D17; (Hamilton, 1960)) score ≥16. Subjects were excluded if they previously had failed to benefit from treatment with the antidepressant being studied, if they had a history of suicide attempt, or if they suffered from any medical illness or received any medication known to significantly affect brain function.	Subjects were excluded if they previously had failed to benefit from treatment with the antidepressant being studied, if they had a history of suicide attempt, or if they suffered from any medical illness or received any medication known to significantly affect brain function.	Mean: 150 mg/day	Placebo	Yes	8 weeks	Unclear	Unclear	Unclear	Unclear	No
Kahn 1998 (150 mg)	No	Yes	To be included, patients had to have had demonstrated symptoms of depression for at least 1 month before study entry and to have minimum scores of 20 on the 21-item Hamilton Rating Scale for Depression (HAM-D) [15] both prestudy and on study day 1 (baseline).		Mean: 150 mg/day	Placebo	Yes	12 weeks	Unclear	Unclear	24.5	25.1	No
Kahn 1998 (200 mg)	No	Yes	To be included, patients had to have had demonstrated symptoms of depression for at least 1 month before study entry and to have minimum scores of 20 on the 21-item Hamilton Rating Scale for Depression (HAM-D) [15] both prestudy and on study day 1 (baseline).		Mean: 200 mg/day	Placebo	Yes	12 weeks	Unclear	Unclear	24.8	25.1	No
Kahn 1998 (75 mg)	No	Yes	To be included, patients had to have had demonstrated symptoms of depression for at least 1 month before study entry and to have minimum scores of 20 on the 21-item Hamilton Rating Scale for Depression (HAMD) [15] both prestudy and on study day 1 (baseline).		Mean: 75 mg/day	Placebo	Yes	12 weeks	Unclear	Unclear	24.3	25.1	No
Learned 2012 (study 1)	Yes	Yes	In both studies, male and female patients (18–64 years old) were required to have a diagnosis of MDE with MDD (single or recur- rent episodes according to the Diagnostic and Statistical Manual of Mental Disorders, 4th Edition, Text Revision [DSM-IV-TR]), with current episode duration of at least two weeks but less than two years. Patients were required to have a Clinical Global Impression-Severity of Illness (CGI-S) score ≥ 4 at the randomi- zation visit and an Inventory of Depressive Symptomatology-Self Reported (IDS-SR) score ≥ 40 (Study 1) or ≥ 25 (Study 2) at the screening and randomization visits, with a change of no more than 25% in IDS-SR between these two visits.	Patients were excluded if the symptoms of a presenting illness were better accounted for by another diagnosis or if the patient had a current DSM-IV-TR diagnosis of any of the following: panic disorder, antisocial or border line personality disorder, bipolar disorder, schizophrenia, or other psychotic disorders. Patients were excluded if they had previously failed an adequate therapeu- tic course of two or more antidepressants, had started psycho-therapy within three months of screening, or had received electroconvulsive therapy or transcranial magnetic stimulation within six months of screening. Patients considered by the investigator to be at risk for suicide or who had any previous suicide attempt or a family history of suicide attempt were excluded. Patients with a positive urine drug screen or a positive blood alco- hol at screening were excluded.	Max: 225 mg/day	Placebo	No	10 weeks	134	126	Unclear	Unclear	No
Lieberman 2008 (225 mg)	Yes	Yes	Men and women, outpatients 18–75 years of age with a primary diagnosis of MDD, based on a psychiatric interview using the 'Diagnostic and Statistical Manual of Mental Disorders, Fourth Edition' (American Psychiatric Association, 1994) criteria, single or recurrent episode, without psychotic features, were eligible for study participation. At baseline and screening, patients were also required to have a minimum HAM-D17 score of 22 and score at least 2 on item one (depressed mood) of HAM-D17, a Clinical Global Impressions-Severity (CGI-S) Scale (Guy, 1976) score of at least 4, and a Raskin Depression Scale (Raskin et al., 1969) score greater than the Covi Anxiety Scale (Lipman, 1982) score.	Patients with comorbid substance use disorders were excluded; however, patients with comorbid generalized anxiety disorder, panic disorder, or social anxiety disorder were allowed to participate as long as MDD was the primary diagnosis. Patients at high risk for suicidal behaviors were excluded.	Max: 225 mg/day	Placebo	No	8 weeks	121	127	25.1	Unclear	No
Luthringer 1996	No	Yes	18 years or older with DSM-III criteria of clinical depression for a minimum of 4 weeks. They had to have a 21-item HAM-D score of at least 20 at both the initial screening and pre-treatment baseline.	Patients were excluded if their affective illness was bipolar or primarily psychotic or if they reported marked suicidal ideation, recent alcohol or drug dependence or abuse, any acute or unstable medical problem, or a history of seizures . Women of childbearing age were required to use a medically approved form of birth control and were admitted to the study only of a beta human chorionic gonadotropin test was negative. Concomitant psychotropic medication was excluded during the study and for at least 7 days before double-blind treatment began (14 days for MAOIs and 30 days for neuroleptics and fluoxetine).	75–225 mg/day	Placebo	Yes	29 days	12	12	28.2	27.1	No
Mendels 1993 - 150 - 200 mg	No	Yes	Outpatients aged 18 to 75 years with a diagnosis of major depression without psychotic features (DSM-III-R) criteria) were screened at 15 centers. Patients were required to have a total score of at least 20 on the 17-item Hamilton Rating Scale for Depression (HAM-D), a score of at least 9 on the Raskin Depression Scale, a score on the Covi Anxiety Scale 10 less than the Raskin score, and a moderate or greater severity of illness on the Clinical Global Impressions (CGI) scale.	Exclusion criteria included active suicidal ideation or suicide attempts in the last 12 months; schizophrenia, organic mental syndromes, or seizure disorders; failure to respond to an adequate course of antidepressant therapy; recent history of alcohol or drug abuse; electroconvulsive therapy within 30 days of the study; monoamine oxidase inhibitors or neuroleptics within 14 days of active drug treatment; and use of other antidepressants or anxiolytics within 7 days of baseline.	150-200mg/day	Placebo	Yes	6 weeks	Unclear	Unclear	25.57	25.39	No
Mendels 1993 - 25 mg	No	Yes	Outpatients between the ages of 18 and 65 years were eligable to participate if they met DSM-III-R criteria for major depression (American Psychiatric Association 1987) and had a minimum score of 20 on the 21-item Hamilton Rating Scale of Depression (HAM-D; Hamilton 1960).	Patients were ineligable for enrollment if they had significant physical illness or mental illnesses other than depression or a history of drug or alcohol dependence within 2 years of the study. Patients were not enrollen if they were suicidal to a degree that precautions against suicide had to be taken. Patients with a history of bipolar disorder or psychosis were excluded from the study.	25mg/day	Placebo	Yes	6 weeks	Unclear	Unclear	25.92	25.39	No
Mendels 1993 - 50 - 75 mg	No	Yes	Outpatients between the ages of 18 and 65 years were eligable to participate if they met DSM-III-R criteria for major depression (American Psychiatric Association 1987) and had a minimum score of 20 on the 21-item Hamilton Rating Scale of Depression (HAM-D; Hamilton 1960).	Patients were ineligable for enrollment if they had significant physical illness or mental illnesses other than depression or a history of drug or alcohol dependence within 2 years of the study. Patients were not enrollen if they were suicidal to a degree that precautions against suicide had to be taken. Patients with a history of bipolar disorder or psychosis were excluded from the study.	50-75mg/day	Placebo	Yes	6 weeks	Unclear	Unclear	25.43	25.39	No
Nemeroff 2007	No	Yes	Participants were outpatients 18 years or older and met Diagnostic and Statistical Manual of Mental Disor- ders, Fourth Edition (DSM-IV) criteria for major depres- sive disorder (American Psychiatric Association, 1994). All patients had symptoms present for at least 1 month before study entry and scored at least 20 on the 21-item Hamilton Rating Scale for Depression (HAM-D21).	Patients were excluded if they had a history or pres- ence of bipolar disorder or any psychotic disorder. Patients with a history of alcohol or substance abuse within the past year were excluded from the study, as were those who had any clinically significant medical disorders or abnormalities detected during the prestudy physical screening that might compromise study participation. Additionally, patients were excluded if they were acutely suicidal to the degree that precautions against suicide were needed. Another cause for exclusion was a history of nonresponse to venlafaxine or fluoxe- tine. Further, any patient who had received either study drug within 6 months prior to starting the double-blind treatment period was excluded. Patients were excluded if they had received any of the following treatments before entering the trial: electroconvulsive therapy within 3 months; any investigational drug or antipsychotic drug within 30 days; astemizole, cisapride, sumatriptan, ter- fenadine, any monoamine oxidase inhibitor, paroxetine, or sertraline within 14 days; any other antidepressant, anxiolytic, sedative-hypnotic drug (except chloral hy- drate), or any other psychotropic drug within 7 days of the start of double-blind treatment; or any other drug with psychotropic effects within 7 days of the start of the double-blind treatment period unless a stable dose of the drug had been maintained for at least 1month (3 months for thyroid or hormonal medications) before study day 1. Pregnant or lactating women were excluded from the study, as were women capable of childbearing who were unwilling to use a medically acceptable form of contraception.	75–225 mg/day	Placebo	Yes	6 weeks	102	102	23.5	23.7	No

Trial ID	Registry/ published protocol	Risk of for-profit bias	Inclusion criteria	Exclusion criteria	Dose range (mg/day)	Control intervention	Placebo washout	Length of intervention period	No. randomised to Venlafaxine	No. randomised to control	Baseline HDRS Venlafaxine	Baseline HDRS control	Co-interventions
Rudolph 1998 (225 mg) + Khan 1991, Schweizer 1991	No	Yes	The study population consisted of psychiatric outpatients between the ages of 18 and 65 who met Diagnostic and Statistical Manual of Mental Disorders (DSM)-lll criteria for major depression. In addition, symptoms of depression had to have been present for at least 1 month before study entry, and the patients had to have minimum and baseline (after washout) scores of 20 on the 21-item Hamilton Rating Scale for Depression (HAM-D 21)	Women of childbearing age were not recruited, not were subjects with bipolar mood disorder (or bipolar ll), schizofrenia, and other psychotic disorders.	Mean: 225 mg/day	Placebo	Yes	6 weeks	Unclear	Unclear	25	25	No
Rudolph 1998 (375 mg) + Khan 1991, Schweizer 1991	No	Yes	The study population consisted of psychiatric outpatients between the ages of 18 and 65 who met Diagnostic and Statistical Manual of Mental Disorders (DSM)-lll criteria for major depression. In addition, symptoms of depression had to have been present for at least 1 month before study entry, and the patients had to have minimum and baseline (after washout) scores of 20 on the 21-item Hamilton Rating Scale for Depression (HAM-D 21)	Women of childbearing age were not recruited, not were subjects with bipolar mood disorder (or bipolar ll), schizofrenia, and other psychotic disorders.	Mean: 375 mg/day	Placebo	Yes	6 weeks	Unclear	Unclear	25	25	No
Rudolph 1998 (75 mg) + Khan 1991, Schweizer 1991	No	Yes	The study population consisted of psychiatric outpatients between the ages of 18 and 65 who met Diagnostic and Statistical Manual of Mental Disorders (DSM)-lll criteria for major depression. In addition, symptoms of depression had to have been present for at least 1 month before study entry, and the patients had to have minimum and baseline (after washout) scores of 20 on the 21-item Hamilton Rating Scale for Depression (HAM-D 21)	Women of childbearing age were not recruited, not were subjects with bipolar mood disorder (or bipolar ll), schizofrenia, and other psychotic disorders.	Mean: 75 mg/day	Placebo	Yes	6 weeks	Unclear	Unclear	26	25	No
Rudolph 1999	No	Yes	The study population consisted of outpatients age 18 years and older who met Diagnostic and Statisti- cal Manual of Mental Disorders (DSM)-IV criteria for major depressive disorder (Task Force on DSM- IV, 1994). The diagnosis of major depressive disor- der was based on a clinical interview of the patient and subsequent completion by the investigator of a worksheet containing the DSM IV criteria. In addi- tion, patients had to have symptoms of depression for at least one month before study entry, and they had to have minimum prestudy and baseline (after washout) scores of 20 on the 21-item Hamilton Rating Scale for Depression (HAM-D21) (Hamilton, 1960).	Patients were excluded from study participa- tion if they had recent (within six months) treatment with or a known hypersensitivity to either of the active study drugs, certain specified medical con- ditions, bipolar mood disorder, a psychotic disorder not associated with depression, or a history of drug or alcohol dependence within a year of study entry. Acutely suicidal patients and pregnant or lactating women were also excluded. Patients whose HAM- D 2 1 score decreased by more than 20% from the prestudy evaluation to the baseline evaluation were excluded from randomization. Each prospective study patient gave written informed consent.	75–225 mg/day	Placebo	Yes	8 weeks	100	98	25	25	No
Schatzberg 2006	No	Yes	Male or female subjects aged 65 years or older and not living in a residential setting were eligible for this study. In addition, eligible par- ticipants met Diagnostic and Statistical Manual for Mental Disorders, Fourth Edition criteria for unipolar depression (single or recurrent, nonpsychotic), with a current episode of at least four weeks in duration; had a 21-item HAM-D (HAM-D21) score ≥20 at the initial visit; and were willing and able to provide informed consent.	Subjects with bipolar disorder, a psychotic disorder not related to depression, current substance abuse or substance dependence within the past year (other than nicotine), current suicidal intent, Mini-Mental Status Examination score ≤18, and patients who had received treatment with fluoxetine or ven-lafaxine in the past six months, electroconvulsive therapy within the prior three months, or any inves-tigational drug or antipsychotic medication within the prior 30 days were excluded from the study. Also excluded were subjects who used astemizole, cisa- pride, sumatriptan, terfenadine, paroxetine, sertra- line, or any monoamine oxidase inhibitor within 14 days, used any other antidepressant, anxiolytic, or sedative–hypnotic drug (except chloral hydrate), or any other psychotropic drug or substance within seven days of the start of the double-blind treatment period. Patients with a known hypersensitivity to venlafaxine or fluoxetine, those with clinically significant hepatic or renal disease, seizure disorder, or myocardial infarction within the prior 6 months, and patients with a severe, acute, or unstable medical illness were not allowed to participate in the study.	Max: 225 mg/day	Placebo	Yes	8 weeks	104	96	24	23	No
Schweizer 1994	No	Yes	Patients aged 18 years or older were recruited who met DSM-III-R criteria for major depression for a mini- mum of 4 weeks. The 21-item Hamilton Rating Scale for Depression (HAM-D) total score had to be at least 20 at both the initial screen evaluation and the pretreatment baseline. The score should not have decreased by more than 20% during the screening period.	Patients were excluded if their affective illness was bipolar, required hospitalization, or was primarily psychotic. Patients also were excluded if they reported marked suicidal ideation recent (in the past 2 years) alcohol or drug dependence or abuse, any acute or unstable medical problem, or a history of seizures. Women capable of becoming pregnant were required to use a medically approved form of birth control and were admitted to the study only if a -human chorionic gonadotropin test was negative.	Max 182 mg/day	Placebo	Yes	6 weeks	Unclear	Unclear	25.5	24.6	No
Sheehan 2009	No	Yes	Participants were selected from patients who were hospitalized before screening. Inpatients aged ≥18 years, who fulfilled criteria of the Diagnostic and Statistical Manual of Mental Disorders, fourth edition (American Psychiatric Association, 1994) for the melancholic sub-type of MDD of at least 1 month duration, were eligible for study enrolment if they scored at least 24 on the 21- item Hamilton Rating Scale for Depression (HAM-D21)(Hamilton, 1960).	Medical illnesses, known hypersensitivity to either study drug, treatment with either study drug within 3 months, or myocardial infarction within 6 months before the start of double-blind therapy. In addition, patients with clinically significant abnormalities on the physical examination, electrocardiogram, laboratory tests, or urine drug test at the screening visit were excluded. Women who were pregnant (positive serum β-human chorionic gonadotropin) or lactating were excluded, and women of childbearing potential were required to use a medically acceptable form of contraception. Furthermore, patients with active suicidal ideation; a history of seizures; the presence or history of an organic mental disorder, mania, or hypomania; or psychotic disorder were excluded. Other reasons for exclusion included electroconvulsive therapy within 3 months, any investigational or antipsychotic drug within 30 days, or astemizole, cisapride, sumatriptan, terfenadine, or any monoamine oxidase inhibitor within 14 days of the start of double-blind therapy. Patients could not have taken any other antidepressant, anxiolytic, sedative-hypnotic, or other psychotropic drug within 2 days before the start of double-blind treatment. Patients also could not have taken any nonpsychopharmacologic drug with psychotropic effects within 2 days before the start of double-blind treatment, unless the dosage had been stable for at least 1 month (3 months for thyroid or other hormones). Patients with a history of alcohol or drug dependence or abuse within 1 year before double- blind treatment were also excluded.	Max: 225 mg/day	Placebo	No	6 weeks	95	95	29.9	29.4	No
Silverstone 1999	No	Yes	Outpatients aged 18 years or older who met DSM-lV criteria for major depressive disorder were eligible if they had a minimum baseline score of 20 on the first 17 items of the 21-item Hamilton Rating Scale for Depression (HAM-D) with not more than a 20% decrese in score between screening and baseline. They also had a minimum score of 8 on the Covi scale and symptoms of depression for at least 1 month before study entry.	Women who were pregnant, lactating, or of childbearing potentialand had a positive beta-human chorionic gonadotropin (β-hCG) pregnancy test result were not included. Also excluded were patients who had a history of clinically significant medical disease or clinically significant abnormalities on a screening physical examination, electrocardiogram (ECG), or laboratory tests. Those who had suicidal tendencies, a history of a seizure disorder, bipolar disorder, or history of mania or any psychotic disorder not associated with depression were also excluded. Other reasons for exclusion were use of any investigational drug or electroconvulsive therapy within 30 days, fluoxetine within 28 days, or a monoamine oxidase inhibitor or paroxetine within 14 days of double-blind treatment. Patients could not have taken any other antidepressant, antopsychotic, anxiolytic, sedative-hypnotic drug, or psychotropic drug or substance within 7 days of the start of double-blind treatment; any non-psychopharmacologic drug with psychotropic effects (e.g., β-adrenergic blockers) within 7 days of baseline unless the dosage had been stable for a minimum of 1 month before double-blind treatment. Patients with a history of drug or alcohol dependence within 2 years or a history of drug abuse within 6 months of the start of double-blind treatment were excluded.	Max: 225 mg/day	Placebo	Yes	12 weeks	128	119	27.6	27.1	No
Thase 1997	No	Yes	Eligible patients (1) were outpatients, (2) aged 18 years or older, (3) satisfied DSM-lV criteria for major depressive disorder for at least 1 month, and (4) had a minimum baseline score of 20 on the 21-item Hamilton Rating Scale for Depression (HAM-D), with not more than a 20% decrease in score between screening and baseline.	Patients were excluded if if they had previously been treated with venlafaxine. Women who were lactating or pregnant (e.g., a positive β-subunit of human chorionic gonadotropin test) were not included. Patients were also excluded if they had a history of clinically significant abnormalities on a screening physical examination, an electrocardiogram (ECG), or laboratory tests. Additional exclusion criteria included acute suicidal tendencies, a history of seizure disorder, a history or presence of a mental disorder due to a general medical condition, bipolar disorder, drug or alcohol abuse or dependence within the past year, or a history of any psychotic disorder not associated with depression. Patients could not have received an investigational drug, an antipsychotic drug, or electroconvulsive therapy within 30 days, fluoxetine within 21 days, or a monoamine oxidase inhibitor within 14 days. Patients could not take any antidepressant, anxiolytic, sedative-hypnotic, or other psychotropic drug or substance within 7 days of the start of double-blind treatment. Use of nonpsychotropic drugs with psychotropic effects (e.g., β-adrenergic blockers) was permitted if the dosage was stable for a minimum of 1 month before double-blind treatment.	Max: 225 mg/day	Placebo	Yes	8 weeks	102	95	25	24	No

Supplementary Table 4: Summary of Findings

Venlafaxine compared to control for adults with major depressive disorder

Patient or population: adults with major depressive disorder

Setting:

Intervention: venlafaxine

Comparison: control

Outcomes	Anticipated absolute effects* (95% CI)		Relative effect (95% CI)	№ of participants (studies)	Certainty of the evidence (GRADE)	Comments
	Risk with control	Risk with venlafaxine				
Suicides or suicide attempts follow-up: range 6 weeks to 8 weeks	8 per 1.000	**5 per 1.000** (2 to 13)	**OR 0.65** (0.25 to 1.71)	1907 (7 RCTs)	⨁◯◯◯ Very low[a,b]	
Serious adverse events follow-up: range 4 weeks to 12 weeks	25 per 1.000	**65 per 1.000** (41 to 104)	**RR 2.66** (1.67 to 4.25)	5526 (22 RCTs)	⨁◯◯◯ Very low[a,b,c]	
Non-serious adverse events follow-up: range 4 weeks to 13 weeks	472 per 1.000	**674 per 1.000** (571 to 797)	**RR 1.43** (1.21 to 1.69)	5483 (24 RCTs)	⨁◯◯◯ Very low[a,b,c]	

***The risk in the intervention group** (and its 95% confidence interval) is based on the assumed risk in the comparison group and the **relative effect** of the intervention (and its 95% CI).

CI: confidence interval; **OR:** odds ratio; **RR:** risk ratio

GRADE Working Group grades of evidence
High certainty: we are very confident that the true effect lies close to that of the estimate of the effect.
Moderate certainty: we are moderately confident in the effect estimate: the true effect is likely to be close to the estimate of the effect, but there is a possibility that it is substantially different.
Low certainty: our confidence in the effect estimate is limited: the true effect may be substantially different from the estimate of the effect.
Very low certainty: we have very little confidence in the effect estimate: the true effect is likely to be substantially different from the estimate of effect.

Explanations

a. Downgraded 2 levels for high risk of bias in included studies.

b. Downgraded 2 levels for imprecision due to Trial Sequential Analysis showing that there was not enough information to confirm or reject a relative risk reduction (RRR) of 20% and the accrued number of participants is below 50% of the diversity-adjusted required information size (DARIS).

c. Downgraded 1 level for indirectness due to differences in measurement of outcome.

Supplementary Table 5: RoB2 table with explanations

	Domain 1	Domain 2	Domain 3	Domain 4	Domain 5
0600B 1-384-US/EU/CA	No information	No information	No information	No information	No information
600-B-367-EU	No information on concealment.	Described as double-blind, but no further details. Not proper ITT.	Unclear or more than 5% missing.	No/inadequate description of outcome measurement. Described as double-blind, but no further details. Unclear if lack of blinding can affect the outcomes.	Protocol/registration /statistical analysis plan not sufficiently detailed.
Alvarez 2012	Random sequence, concealed.	Described as double-blind, but no further details. Not proper ITT.	Unclear or more than 5% missing.	No/inadequate description of outcome measurement. Described as double-blind, but no further details. Unclear if lack of blinding can affect the outcomes.	Protocol/registration /statistical analysis plan not sufficiently detailed.
Claghorn 1990	Random sequence, concealed.	Blinding unclear. Not proper ITT.	Unclear or more than 5% missing.	Adequate description of outcome measurement. Blinding unclear. Unclear if lack of blinding can affect the outcomes.	Protocol/registration /statistical analysis plan not available.
Cunningham 1994	Random sequence, concealed.	Blinding unclear. Not proper ITT.	Unclear or more than 5% missing.	Adequate description of outcome measurement. Blinding unclear. Unclear if lack of blinding can affect the outcomes.	Protocol/registration /statistical analysis plan not available.
Cunningham 1997	No information on sequence/concealment .	Described as double-blind, but no further details. Not proper ITT.	Unclear or more than 5% missing.	No/inadequate description of outcome measurement. Described as double-blind, but no further details. Unclear if lack of blinding can affect the outcomes.	Protocol/registration /statistical analysis plan not available.
EudraCT 2004-000562-13	No information on sequence/concealment .	Described as double-blind, but no further details. Not proper ITT.	Unclear or more than 5% missing.	No/inadequate description of outcome measurement. Described as double-blind, but no further details. Unclear if lack of blinding can affect the outcomes.	Protocol/registration /statistical analysis plan not sufficiently detailed.

	Domain 1	Domain 2	Domain 3	Domain 4	Domain 5
EudraCT 2007-007025-51	No information on sequence/concealment.	Described as double-blind, but no further details. Analysis inadequately described.	Unclear or more than 5% missing.	No/inadequate description of outcome measurement. Described as double-blind, but no further details. Unclear if lack of blinding can affect the outcomes.	Protocol/registration/statistical analysis plan not available.
Guelfi 1995	No information on sequence/concealment.	Described as double-blind, but no further details. Not proper ITT.	Unclear or more than 5% missing.	No/inadequate description of outcome measurement. Described as double-blind, but no further details. Unclear if lack of blinding can affect the outcomes.	Protocol/registration/statistical analysis plan not available.
Hewett 2009	Random sequence, concealed.	Described as double-blind, but no further details. Not proper ITT.	Unclear or more than 5% missing.	No/inadequate description of outcome measurement. Described as double-blind, but no further details. Unclear if lack of blinding can affect the outcomes.	Protocol/registration/statistical analysis plan not sufficiently detailed.
Hewett 2010	Random sequence, concealed.	Described as double-blind, but no further details. Not proper ITT.	Unclear or more than 5% missing.	No/inadequate description of outcome measurement. Described as double-blind, but no further details. Unclear if lack of blinding can affect the outcomes.	Protocol/registration/statistical analysis plan not available.
Higuchi 2016	No information on sequence/concealment.	Described as double-blind, but no further details. Not proper ITT.	Unclear or more than 5% missing.	No/inadequate description of outcome measurement. Described as double-blind, but no further details. Unclear if lack of blinding can affect the outcomes.	Protocol/registration/statistical analysis plan not sufficiently detailed.
Khan 1998	No information on sequence/concealment.	Described as double-blind, but no further details. Not proper ITT.	Unclear or more than 5% missing.	No/inadequate description of outcome measurement. Described as double-blind, but no further details. Unclear if lack of blinding can affect the outcomes.	Protocol/registration/statistical analysis plan not available.

	Domain 1	Domain 2	Domain 3	Domain 4	Domain 5
Learned 2012	No information on sequence/concealment.	Described as double-blind, but no further details. Not proper ITT.	Unclear or more than 5% missing.	No/inadequate description of outcome measurement. Described as double-blind, but no further details. Unclear if lack of blinding can affect the outcomes.	Protocol/registration /statistical analysis plan not sufficiently detailed.
Lieberman 2008	No information on sequence/concealment.	Described as double-blind, but no further details. Not proper ITT.	Unclear or more than 5% missing.	No/inadequate description of outcome measurement. Described as double-blind, but no further details. Unclear if lack of blinding can affect the outcomes.	Protocol/registration /statistical analysis plan not sufficiently detailed.
Luthringer 1996	No information on sequence/concealment.	Described as double-blind, but no further details. Not proper ITT.	Unclear or more than 5% missing.	No/inadequate description of outcome measurement. Described as double-blind, but no further details. Unclear if lack of blinding can affect the outcomes.	Protocol/registration /statistical analysis plan not available.
Mendels 1993	No information on sequence/concealment.	Described as double-blind, but no further details. Not proper ITT.	Unclear or more than 5% missing.	No/inadequate description of outcome measurement. Described as double-blind, but no further details. Unclear if lack of blinding can affect the outcomes.	Protocol/registration /statistical analysis plan not available.
Nemeroff 2007	No information on sequence/concealment.	Described as double-blind, but no further details. Not proper ITT.	Unclear or more than 5% missing.	No/inadequate description of outcome measurement. Described as double-blind, but no further details. Unclear if lack of blinding can affect the outcomes.	Protocol/registration /statistical analysis plan not available.
Rudolph 1998	No information on concealment.	Described as double-blind, but no further details. Not proper ITT.	Unclear or more than 5% missing.	No/inadequate description of outcome measurement. Described as double-blind, but no further details. Unclear if lack of blinding can affect the outcomes.	Protocol/registration /statistical analysis plan not available.

	Domain 1	Domain 2	Domain 3	Domain 4	Domain 5
Rudolph 1999	No information on concealment.	Described as double-blind, but no further details. Not proper ITT.	Unclear or more than 5% missing.	No/inadequate description of outcome measurement. Described as double-blind, but no further details. Unclear if lack of blinding can affect the outcomes.	Protocol/registration /statistical analysis plan not available.
Schatzberg 2006	No information on concealment.	Described as double-blind, but no further details. Not proper ITT.	Unclear or more than 5% missing.	No/inadequate description of outcome measurement. Described as double-blind, but no further details. Unclear if lack of blinding can affect the outcomes.	Protocol/registration /statistical analysis plan not available.
Schweizer 1994	Random sequence, concealed.	Blinding unclear. Not proper ITT.	Unclear or more than 5% missing.	Adequate description of outcome measurement. Blinding unclear. Unclear if lack of blinding can affect the outcomes.	Protocol/registration /statistical analysis plan not available.
Sheehan 2009	No information on concealment.	Described as double-blind, but no further details. Not proper ITT.	Unclear or more than 5% missing.	No/inadequate description of outcome measurement. Described as double-blind, but no further details. Unclear if lack of blinding can affect the outcomes.	Protocol/registration /statistical analysis plan not available.
Silverstone 1999	Random sequence, concealed.	Blinded participants and caregivers. Not proper ITT.	Unclear or more than 5% missing.	No/inadequate description of outcome measurement. Unclear binding of outcome assessors. Unclear if lack of blinding can affect the outcomes.	Protocol/registration /statistical analysis plan not available.
Thase 1997	Random sequence, concealed.	Described as double-blind, but no further details. Not proper ITT.	Unclear or more than 5% missing.	No/inadequate description of outcome measurement. Described as double-blind, but no further details. Unclear if lack of blinding can affect the outcomes.	Protocol/registration /statistical analysis plan not available.

Supplementary Table 6: Individual serious adverse events

Events	Number of trials reporting the event	Venlafaxine events	Venlafaxine analysed	Control events	Control analysed	Relative risk (95% CI)	P-value	Number needed to harm
**Sexual dysfunction**	8	62	677	5	472	6.49 (3.02,13.93)	< 0.01	12
**Anorexia**	9	128	1389	24	1024	3.23 (1.75,5.97)	< 0.01	14
**Anxiety**	9	58	1210	27	923	1.40 (0.57,3.44)	0.47	
**Discontinuation symptoms**	2	1	73	0	78	3.12 (0.33,29.66)	0.32	
**Fall**	2	2	211	0	130	2.16 (0.23,20.60)	0.50	
**Hypertension**	3	10	283	3	206	1.82 (0.44,7.48)	0.41	
**Hypotension**	2	8	519	1	266	2.78 (0.47,16.30)	0.26	
**Intentional overdose**	3	1	332	1	254	0.90 (0.16,5.09)	0.90	
**QTc**	2	1	270	1	263	1.00 (0.08,12.26)	1.00	
**Syncope**	2	2	352	2	280	0.74 (0.11,5.02)	0.75	
**Worsening of depression**	5	6	717	7	638	0.65 (0.16,2.73)	0.56	

Supplementary Table 7: Serious adverse events in the included trials

Trial ID	Venlafaxine group		Control group	
	Numbers and types of serious adverse events	Proportion of participants with a serious adverse event	Numbers and types of serious adverse events	Proportion of participants with a serious adverse event
0600B 1-384-US/EU/CA	1 suicide attempt	1 out of 180	3 suicide attempts	3 out of 68
600A-:302-US, CA/302	None mentioned	0 out of 72	1 suicide attempt	1 out of 76
600-B-367-EU	6 hypertension, 2 anorexia, 2 anxiety, 2 abnormal ejaculation (men), 2 worsening of depression, 1 coma, 1 psychotic depression, 1 hospitalisation for depression, 1 libido decreased, 1 hypotension, 1 syncope, 1 tinnitus, 1 suicide, 1 hospitalisation for anxiety, 1 humerus fracture, 1 accidental injury, 1 fall, 1 urinary retention, 1 trismus	* out of 165	3 hypertension, 2 anorexia, 2 anxiety, 2 hospitalisation for anxiety, 1 hospitalisation for depression, 1 syncope, 1 arthritis, 1 amnesia, 1 suicide attempt, 1 loss of consciusness related to high blood alcohol levels	* out of 83
Alvarez 2012	7 anorgasmia, 4 ejaculation delayed, 4 erectile dysfunction, 1 brain tumor	* out of 113	2 sexual dysfunction	* out of 105
Claghorn 1990	1 pregnancy, 1 discontinuation symptoms	2 out of 79	None mentioned	0 out of 80
Cunningham 1994	3 hypertension, 1 albuminuria	4 out of 72	1 hospitalisation for depression, 1 QT-prolongation, 1 intentional overdose, 1 infection (mononucleosis)	4 out of 76
Cunningham 1997	16 anorexia, 12 abnormal ejaculation,	* out of 193	4 anorexia	4 out of 100
EudraCT 2004-000562-13	6 anorexia, 2 impotence, 1 extrauterine pregnancy, 1 cervix carcinoma	* out of 127	2 anorexia, 1 panic attack	* out of 120
Guelfi 1995	1 rash with mucosal lesions, 1 grand mal seizure, 1 hypertension, 1 fall	4 out of 46	None mentioned	0 out of 47
Hewett 2009	6 anxiety, 1 syncope, 1 suicide attemtp	* out of 187	9 anxiety, 3 depression, 1 seizure, 1 syncope, 1 convulsion, 1 blood TSH increase	* out of 197
Hewett 2010	9 anorexia, 1 worsening of depression, 1 pyelonephritis, 1 QTc-prologation, 1 gastritis	* out of 198	2 anorexia, 1 suicidal depression	* out of 187
Higuchi 2016	7 hypotension, 1 suicide, 1 méniéres disease	* out of 354	1 hypotension, 1 suicide, 1 anemia	* out of 183
Learned 2012	3 anxiety	3 out of 133	5 anxiety, 1 ovarian cyst	* out of 126
Lieberman 2008 (225 mg)	18 anorexia, 4 impotence	* out of 117	1 anorexia, 1 impotence	* out of 123
Mendels 1993	13 anxiety, 11 sexual dysfunction	* out of 234	1 anxiety	1 out of 78
Nemeroff 2007	10 anxiety	1 out of 100	1 anxiety	1 out of 102
Rudolph 1998	40 anorexia, 10 anxiety	* out of 266	2 anorexia	2 out of 92
Rudolph 1999	9 anorexia	9 out of 100	4 anorexia	4 out of 98
Schatzberg 2006	9 libido decreased, 2 anxiety	* out of 100	4 anxiety, 1 libido decreased	* out of 96
Sheehan 2009	12 anxiety, 2 suicidal ideation, 1 worsening of depression, 1 intentional overdose	*out of 95	4 anxiety, 2 suicidal ideation, 2 worsening of depression, 1 allergic reaction, 1 nose bleed	* out of 95
Schweizer 1994	1 suicide attempt, 1 discontinuation symptoms, 1 maculopapular rash	3 out of 73	1 leukopenia	1 out of 78
Silverstone 1999	13 anorexia	13 out of 128	3 anorexia	3 out of 119
Thase 1997	15 anorexia, 8 abnormal ejaculation/orgasm (men), 5 impotence, 4 anorgasmia (women), 2 abnormal ejaculation/orgasm (women)	* out of 95	4 anorexia, 1 anorgasmia (women), 1 abnormal ejaculation/orgasm (men)	* out of 102

* The overall proportion of serious adverse events was unclear.

Supplementary Table 8: Individual non-serious adverse events

Events	Number of trials reporting the event	Venlafaxine events	Venlafaxine analysed	Control events	Control analysed	Relative risk (95% CI)	P-value	Number needed to harm
Nausea	23	981	3270	275	2394	2.72 (2.26,3.28)	< 0.01	5
Dry mouth	21	481	2884	165	2198	2.16 (1.71,2.74)	< 0.01	10
Dizziness	20	454	3051	129	2160	2.49 (1.90,3.26)	< 0.01	11
Somnolence	18	415	2768	125	1888	2.23 (1.78,2.78)	< 0.01	11
Sweating	20	314	2660	60	2118	3.99 (2.88,5.54)	< 0.01	11
Constipation	18	310	2595	94	1892	2.24 (1.64,3.04)	< 0.01	14
Nervousness	11	157	1360	47	949	2.20 (1.43,3.40)	< 0.01	15
Insomnia	19	340	2853	140	2064	1.73 (1.37,2.19)	< 0.01	19
Asthenia	16	173	2132	75	1696	1.78 (1.30,2.43)	< 0.01	27
Tremor	11	69	1287	23	1156	2.30 (1.22,4.32)	0.01	29
Appetite decreased	3	21	589	6	405	2.52 (1.04,6.09)	0.04	47
Abdominal pain	4	24	844	29	573	0.58 (0.22,1.57)	0.28	
Abnormal dreams	2	21	358	1	183	2.97 (0.51,17.25)	0.23	
Abnormal vision	7	33	765	14	671	1.95 (0.85,4.47)	0.11	
Abnormality of accommod	3	11	283	3	206	2.39 (0.72,7.99)	0.16	
Agitation	5	15	518	4	428	2.24 (0.55,9.03)	0.26	
Back pain	3	7	394	14	299	0.44 (0.18,1.07)	0.07	
Blood pressure increased	2	14	456	7	279	1.36 (0.50,3.70)	0.55	
Bronchitis	2	3	211	0	130	1.84 (0.26,13.00)	0.54	
Coughing	2	4	267	5	179	0.59 (0.15,2.23)	0.43	
Diarrhoea	14	126	1838	105	1449	1.00 (0.78,1.28)	0.99	
Dyspepsia	6	40	700	51	602	0.72 (0.44,1.16)	0.17	
Flatulence	2	0	172	1	90	0.39 (0.05,3.22)	0.38	
Headache	17	502	2384	384	1776	1.01 (0.69,1.17)	0.93	
Hypercholesterolemia	2	1	211	2	130	0.51 (0.08,3.07)	0.46	
Hypochromic anaemia	2	2	211	0	130	1.50 (0.21,10.91)	0.69	
Increased salivation	2	2	211	0	130	1.50 (0.21,10.91)	0.69	
Infection	6	49	771	37	533	0.93 (0.61,1.43)	0.75	
Influenza	5	16	611	17	511	0.80 (0.30,2.14)	0.66	
Malaise	2	20	519	6	266	1.31 (0.37,4.69)	0.67	
Nasopharyngitis	4	88	798	66	601	0.83 (0.57,1.19)	0.31	
Neck pain	2	2	211	1	130	1.02 (0.19,5.46)	0.98	
Pain	2	6	211	2	130	1.49 (0.28,7.93)	0.64	
